# Can Machine Learning Predict the Phase Behavior of Surfactants?

**DOI:** 10.1021/acs.jpcb.2c08232

**Published:** 2023-04-12

**Authors:** Joseph C. R. Thacker, David J. Bray, Patrick B. Warren, Richard L. Anderson

**Affiliations:** † The Hartree Centre, STFC Daresbury Laboratory, Warrington, WA4 4AD, United Kingdom; ‡ Department of Chemistry, University of Liverpool, Crown Street, Liverpool, L69 7ZD, United Kingdom

## Abstract

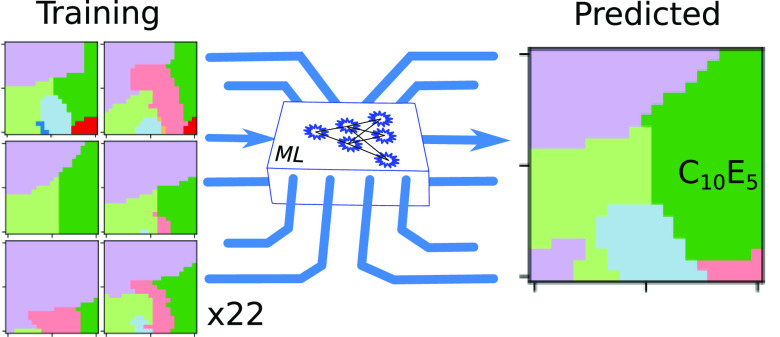

We explore the prediction of surfactant phase behavior using state-of-the-art machine learning methods, using a data set for twenty-three nonionic surfactants. Most machine learning classifiers we tested are capable of filling in missing data in a partially complete data set. However, strong data bias and a lack of chemical space information generally lead to poorer results for entire *de novo* phase diagram prediction. Although some machine learning classifiers perform better than others, these observations are largely robust to the particular choice of algorithm. Finally, we explore how *de novo* phase diagram prediction can be improved by the inclusion of observations from state points sampled by an analogy to commonly used experimental protocols. Our results indicate what factors should be considered when preparing for machine learning prediction of surfactant phase behavior in future studies.

## 1 Introduction

The phase behavior of surfactants is of vital importance in formulated product design and development. Highly viscous intermediate phases can clog up processing equipment, cause inhomogeneous mixing, and adversely impact dispersion and dissolution of concentrated products. These undesired effects can be hugely costly in terms of the manufacture and quality control of formulated products. As such, the prediction of surfactant phase behavior has long been a critical challenge in industry. This is increasingly the case with the trend toward decarbonization of the surfactants market, which is rapidly moving away from petrochemical and traditional plant-based feedstocks (e.g. palm oil), toward more sustainably sourced raw materials.^1,2^ Now, more than ever, organizations are using high-throughput experimentation aided by computer-aided formulation and design tools to expedite the screening of new candidate surfactant formulations: the environment cannot afford the same development time scales (many years) that were used for the previous generation of formulated products.

Over the last two and a half decades, a significant amount of progress has been made in modeling surfactant behavior through a variety of methods, including traditional molecular dynamics type approaches^3^ and coarse-grained schemes such as dissipative particle dynamics.^4^ Simulations of model surfactants are continually improving and are now able to more accurately capture properties such as, *inter alia*, critical micelle concentrations,^5−8^ micelle morphology,^9^ and aggregation number distributions,^10^ and phase behavior, both for single component systems and more recently for mixtures.^11^ Nevertheless, multiple challenges remain when using these approaches, such as the availability of appropriate force fields, the accessibility of relevant time scales, and the computational cost to achieve a positive result.^12,13^

Machine learning (ML) approaches offer a potentially exciting route to rapid prediction of surfactant properties based on models built from pre-existing information, that potentially avoid some of the challenges held by simulation approaches. Indeed, ML has been used extensively to solve chemical problems in everything from developing interatomic potentials for molecular simulation,^14−17^ the prediction of quantum chemical properties using SMILES strings,^18^ the optimization of catalyst mixtures for hydrogen evolution using a “robotic chemist”,^19^ the discovery of new antibiotics,^20^ and many more.

ML has also been applied to phase diagram prediction.^21−24^ For example, a ML force field has been used to compute the phase diagram for uranium for temperatures and pressures up to 1600 K and 800 GPa, respectively, with relatively good accuracy at lower temperatures but diverging from density functional theory predictions at higher temperatures and pressures.^25^ That work differs from that presented here, in that we are looking to compute the phase diagram directly from a data set of experimentally determined phase diagrams, as opposed to predicting the phase indirectly using a ML force field. This has also been achieved by Aghaaminiha and co-workers who have presented a ML approach to estimate the phase diagrams for three-component lipid mixtures with good success.^23^

For nonionic surfactants, Bell^26^ explored the use of ML to fill in the gaps of phase diagrams in the temperature–composition plane. Using a ML method known as Recursive Partitioning (Decision Trees), trained on the surfactant’s critical packing parameter (CPP) values, and the phase behavior discretized in terms of temperature and weight fraction, he was able to demonstrate a classifier that could predict the phase at a given point on a phase diagram.

In the present study, we build upon Bell’s study (modified as described below) to evaluate the capabilities of eight ML approaches to predict the phase behavior, in the context of two key challenges: *gap filling* – filling in missing data in a partially complete data set of surfactant phase behavior; *de novo* prediction – generating a complete phase diagram for a new surfactant from knowledge of other surfactant phase behavior, akin to an expert drawing a phase diagram from scratch, based on intuition and prior experience with other surfactants. The second of these is undoubtedly much more challenging for ML than the first, as no information on the new surfactant’s behavior is permitted to be used during training. We build upon the latter challenge by considering laboratory sampling strategies that might be deployed to improve these predictions.

We arrange the article as follows. We first summarize the aspects of surfactant phase behavior that are relevant for the present work. Under Methods, we outline our ML approaches and the differences in the data set used in this work versus that of Bell. In Results and Discussion we present the outcomes of the gap filling and *de novo* challenges and comment on the performance of the various ML classifiers we tested, exploring the root causes of the same. Then, refining the *de novo* prediction challenge, we explore different sampling strategies, which might be in line with those used in a laboratory setting that can aid prediction. Finally, we present our conclusions where we discuss the benefits and key challenges of the adopted methods.

## 2 Surfactant Phase Behavior

**Figure 1:**
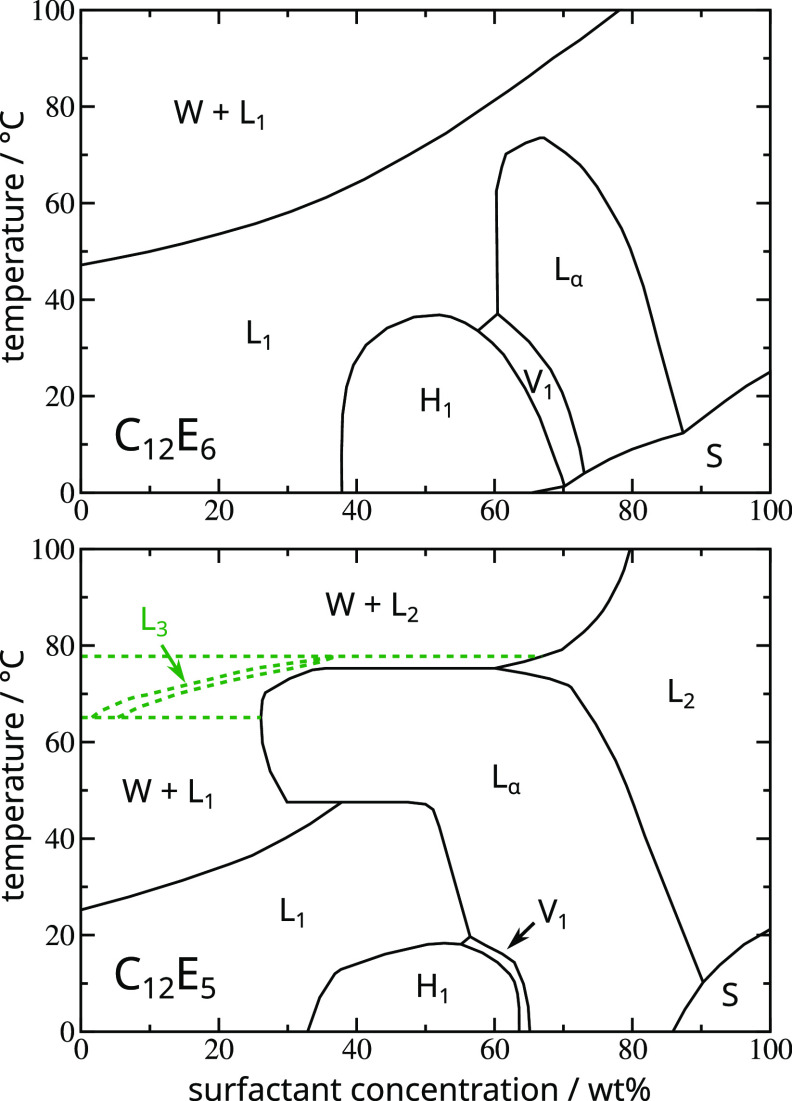
Surfactant phase diagrams in the temperature–composition plane for C_12_E_6_ and C_12_E_5_. In the latter system the complex of phase boundaries around the L_3_ sponge phase (green dotted lines) is ignored for the present purposes. Phase diagrams reproduced with permission from ref (28). Copyright 1983 Royal Society of Chemistry.

In surfactant science, phase diagrams map out the liquid crystalline order of surfactant mixtures and solutions as a function of temperature, pressure, and composition. In the present work we shall be concerned with the phase behavior of binary surfactant/water mixtures (i.e., aqueous solutions) in the temperature–composition plane at ambient pressure. In general terms, the observed phases and their coincidences must satisfy certain strict conditions, such as the Gibbs’ phase rule, which ultimately derive from thermodynamic considerations. This means that surfactant phase diagrams are rather tightly constrained, and tend to look similar in many respects. Two representative examples are shown in Figure 1.

If we consider, for example, increasing surfactant concentration along the 25 °C isotherm in the alkyl ethoxylate C_12_E_6_–water system (Figure 1, upper), we first encounter an isotropic liquid (L_1_) phase that comprises (above the critical micelle concentration) micelles with a liquid-like structural disorder. We note that micelles can elongate as the concentration increases (i.e., above a sphere-to-rod transition) and even form long worm-like structures, but such morphological changes are not phase boundaries in the equilibrium phase diagram, although they may profoundly affect the rheology. Eventually, packing considerations typically force the micelles to order, resulting in a new phase: in our example this happens at around 40 wt % where an hexagonal phase (H_1_) appears (columnar or rod-like aggregates, packed with long-range hexagonal order). A little more specific to this system, but often quite common, there next stabilizes a bicontinuous cubic phase (V_1_) (three-dimensional long-range order and a gyroid structure). At higher concentrations still, a lamellar (L_α_) phase appears (stacked fluid sheets or bilayers). Eventually, as the water content diminishes, the lamellar phase no longer persists as the most stable phase and instead the system re-enters an isotropic liquid phase, in this case, formed by inverse micelles. Finally, although this depends on the melting point of the pure surfactant, a two-phase region (S) may be encountered where the solution coexists with solid surfactant below the so-called Krafft boundary (i.e., the solubility limit of the solid surfactant in water).^27^ Note that the lyotropic liquid crystal phase transitions (L_1_–H_1_, etc.) are usually weakly first-order, and *sensu stricto*, the corresponding phase boundaries should be drawn as narrow two-phase coexistence regions. For the purposes of this work though, we do not resolve such fine details.

The lyotropic liquid crystal phases often “melt” on increasing temperature, so that these regions appear as “domes” in the phase diagram. Bearing in mind that the sides of the domes are narrow two-phase regions, the top of such a dome is actually an *azeotrope*, where the liquid crystal melts directly into an isotropic liquid of the same composition. A consequential feature, seen for example in the C_12_E_6_–water system, is that the isotropic liquid phase is contiguous across the top of the liquid crystal domes: it comprises a single phase (L_1_).

At elevated temperatures, it is quite common to see a “cloud curve” in these nonionic surfactant solutions. This reflects the appearance of a two-phase coexistence region in which a surfactant solution (often as micellar rods or worms) coexists with essentially pure water (strictly, a very dilute surfactant solution). This closely resembles the phase behavior of some water-soluble polymers such as poly ethoxylates, which as is well-known exhibit in aqueous solution a lower critical solution temperature and a liquid–liquid demixing transition on increasing temperature.^28^

In some systems, this cloud curve “collides” with the lyotropic liquid crystal domes. An example is the C_12_E_5_–water system (Figure 1, lower). In this case, two isotropic liquid phases (L_1_ and L_2_) should be formally distinguished in the phase diagram, although they share the same symmetry and lack of structural order. Handled naïvely, this can introduce an element of data bias, which we shall discuss further below. Another common feature in this class of phase diagram is the occasional appearance of exotic but often just marginally stable dilute phases at low concentrations, such as (here) the L_3_ sponge phase. For the present purposes we follow Bell in subsuming these into a generic two-phase “W + L” cloud region.^26^

We note briefly the term “isotropic liquid phase” as used here refers to the lack of optical anisotropy and relative fluidity of the L_1_ and L_2_ phases. In contrast, the hexagonal and lamellar phases are not only typically much more viscous or even paste-like, but are also optically anisotropic and can be recognized by their striking “textures” in polarizing light microscopy.^29^ On the other hand, intermediate phases such as the bicontinuous cubic phase, while being optically isotropic because of their high symmetry, are usually easily distinguished from the L_1_ and L_2_ phases by their extreme gel-like rigidity.

The situation for these surfactants is quite typical, but in other systems, still further phases may be observed such as a cubic micellar phase (I_1_), typically found in between the L_1_ and H_1_ phases, and an inverse bicontinuous phase with long-range structural order (V_2_).

The experimental determination of the phases in these systems can follow “quick-and-dirty” methods such as “flooding” or contact penetration scans, which can rapidly establish the appearance and sequence of phases,^27^ but give no information on the positions of the phase boundaries unless augmented by additional information such as refractive index measurements (i.e., a “quantitative” penetration scan).^30^ More traditional methods include exhaustively preparing samples at varied compositions and examining the phase behavior on a hot-stage microscope; these studies are often supplemented by crude rheological characterization of the samples. Finally, to identify fully the structural units in difficult-to-classify intermediate phases, one may have to resort to small-angle X-ray scattering.^31^ The laborious and repetitive nature of much of this work is what renders the experimental determination of surfactant phase diagrams a costly exercise, and inspires attempts to complement these traditional approaches by methods that employ computer simulations or utilize ML approaches.

We note that since the concentration and temperature gradients could in principle be arranged orthogonally, it is conceivable that one could develop a “one-shot” experimental method to determine the complete phase diagram in a single experiment. While such an approach has been successfully applied to polymer blends,^32^ to our knowledge no such equivalent has been published for surfactant solutions. In part this may be because such an experiment would be more difficult in the surfactant case, since a surfactant concentration gradient relaxes by diffusion (collective *D* ∼ 10^–10^ m^2^ s^–1^) much faster than the corresponding composition gradient in a polymer blend (collective *D* ≲ 10^–12^ m^2^ s^–1^).

## 3 Methods

### 3.1 Machine Learning Methods

In our study, we test eight ML classifiers in the prediction of surfactant phase behavior. These are K Nearest Neighbors (Nearest Neighbors hereafter);^33^ Linear Support Vector (Linear SVM);^34^ Radial Basis Function Support Vector (RBF SVM);^35^ Decision Tree (Decision Trees);^36−38^ Neural Network (Neural Network);^39^ Random Forest (Random Forest);^40^ AdaBoost (AdaBoost);^41^ and Naive Bayes^42^ (Naive Bayes). Details of these methods are given in the provided references, and a recent review by Sarker discusses ML algorithms and their use in real-world applications.^43^ These algorithms were deployed using Sci-Kit Learn,^44^ a ML python package.

The above choices allow us to explore multiple methods, to understand their relative performance, and to identify the limitations of the current data set. We hope that this sets a standard for extending this approach to a wider range of surfactants at a later date.

### 3.2 Data Set

Our starting point is analogous to the data set collected by Bell on 23 nonionic surfactant phase diagrams.^26^ This data covers binary phase diagrams in the temperature–composition plane, for aqueous surfactant solutions, where the surfactant (*S*_*f*_) is from alkyl ethoxylate families (formulas given in Table 1 using alkyl C, ethoxylate E, methyl Me and glyceryl G unit notation). Following Bell, each phase diagram is digitized on a grid of 21 × 21 = 441 sample points (**x**(*S*_*f*_;*w*,*T*)) with axes of surfactant weight fraction (*w*) and temperature (*T*), where *w* ∈ [0, 100% w/w] with 5% w/w increments and *T* ∈ [0, 100 °C] with 5 °C increments. There is thus a grand total of 23 × 441 = 10143 data points. The labeled point annotation *y*(**x**) gives the phase at the given state point, for which there are nine options corresponding to the surfactant phase state (*C*). The surfactants *S*_*f*_ and observed phases *C* (with numbers of state points) are listed in Table 1. The numbers in the L_1_ and L_2_ columns indicate the “before” and “after” values (the latter being in brackets), on making the L_1_ → L_2_ replacements described below. We extend the original Bell data set as follows:

- For C_8_E_4_, there was originally a distinction between points labeled “L_1_” (*T* ≥ 30 °C) and points labeled “M” for “miscible” (*T* < 30 °C). This designation derives from Figure 2 in Mitchell et al.,^45^ which only displays the phase behavior in the range 30–100 °C, whereas in the accompanying figure caption it is stated that between 0 and 30 °C the liquids are miscible. Hence, it seems to us that the “M” designation is spurious, since it is really the L_1_ phase, and therefore for the present work we relabeled the “M” points in Bell’s original data set as “L_1_” (thus, there are no longer any data points labeled “M”).
- A more subtle issue already mentioned concerns the labeling of the isotropic liquid phases. Recall from the above that in some cases there is a single contiguous region labeled L_1_, and in other cases there are two isotropic liquid phases separated by an intervening liquid crystal phase, in which case they are separately labeled L_1_ and L_2_. Additionally, in four cases (i.e., C_12_E_2_, C_12_E_3_, C_16_E_4_, (C_7_)_2_GE_8_Me), there is an L_2_ phase, but no L_1_ phase.

**Figure 2:**
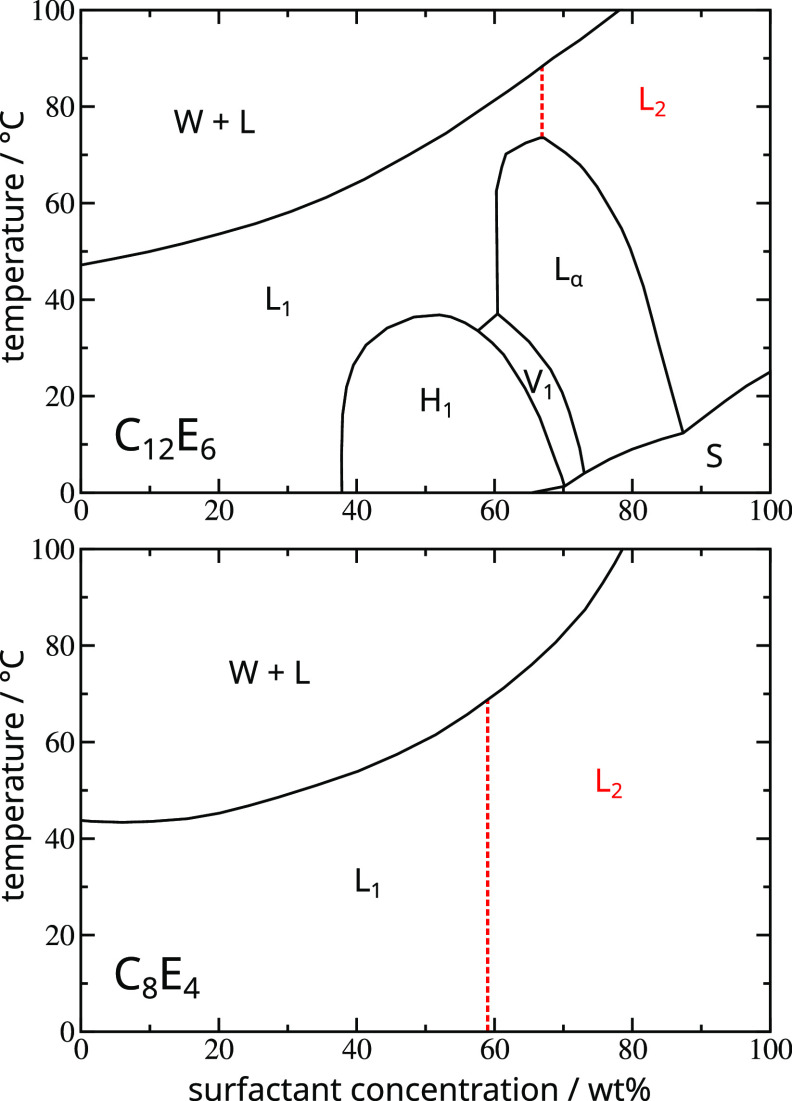
Relabeling L_1_ → L_2_ examples: in the C_12_E_6_–water system, the line (red dashed) ascending from the L_α_ azeotrope separates the designated L_2_ region; in C_8_E_4_–water system, where there are no liquid crystal phases, the corresponding line is the mean concentration of the azeotropes listed in Table 2. Phase diagrams reproduced with permission from ref (28). Copyright 1983 Royal Society of Chemistry.

**Table 1:** List of Surfactants and Their Observed Phases (Numbers of State Points) Used in the Present Study^a^

surfactant (*S*_*f*_)	ref	W + L	L_1_	L_α_	L_2_	H_1_	I_1_	V_1_	V_2_	S
C_8_E_4_	(45)	157	284 (122)		(162)					
C_8_E_6_	(46)	55	371 (172)		(199)	12				3
C_10_E_5_	(47)	143	252 (154)	13	(98)	33				
C_10_E_6_	(47)	99	295 (136)	9	(159)	38				
C_12_E_2_	(48)	354		37	45				5	
C_12_E_3_	(45)	265		81	90					5
C_12_E_4_	(45)	179	28	141	88					5
C_12_E_4_Me	(48)	346	6	59	30					
C_12_E_5_	(45)	138	76	113	82	20		3		9
C_12_E_6_	(45)	114	212 (125)	46	(87)	41		7		21
C_12_E_6_Me	(48)	228	153 (88)	24	(65)	21		4		11
C_12_E_8_	(45)	41	293 (157)		(136)	62	7	6		32
C_12_E_8_Me	(48)	157	208 (99)		(109)	51	5			20
C_16_E_4_	(45)	155		66	73					147
C_16_E_8_	(45)	62	133	68	55	54	2	10		57
C_16_E_12_	(45)	7	245 (157)		(88)	83	34	12		60
(C_6_)_2_GE_8_Me	(49)	240	187 (93)	12	(94)	2				
(C_7_)_2_GE_6_Me	(49)	338		47	56					
(C_7_)_2_GE_8_Me	(49)	270	54	52	65					
(C_7_)_2_GE_10_Me	(49)	195	81	58	95	12				
(C_8_)_2_GE_8_Me	(49)	264	32	84	61					
C_12_G(E_4_Me)_2_	(50)	200	224 (127)		(97)	1	16			
C_14_G(E_4_Me)_2_	(50)	189	206 (106)		(100)	23	23			
No. of surfactants		23	19	16	11 (23)	14	6	6	1	11
No. of state points		4196	3340 (1946)	910	740 (2134)	453	87	42	5	370

a The data set is derived from Bell,^26^ but we also provide primary references. Bracketed values are the revised numbers under L_1_ → L_2_ relabeling.

Concerning the latter point, given the somewhat arbitrary labeling of these phases, we therefore envisaged that one could remove an element of data bias by being more systematic about it, and in those cases where there is only a single contiguous isotropic liquid region, “rebranding” the L_1_ phase at high concentrations as L_2_. To do this we need some criterion. For this we fixed on the most obvious one, which is to introduce an artificial boundary by extrapolating from the highest point (i.e., the azeotrope) on the highest lyotropic liquid crystal dome, for example the L_α_ azeotrope in the C_12_E_6_–water system (Figure 2, upper). These azeotropes were picked out by hand and are listed in Table 2. To make the modified data set therefore, in those systems where there is not already a designated L_2_ phase, we relabeled all the “L_1_” points at concentrations higher than the listed azeotrope concentration as “L_2_”. For C_8_E_4_, there are no liquid crystal phases and consequently no azeotrope to extrapolate from. For this edge case we therefore somewhat arbitrarily chose the mean of the observed azeotrope concentrations and set the relabeling boundary at a weight fraction 0.59 (Figure 2, lower), although in fact the boundary can be placed anywhere in the 0.55–0.59 range without changing the results because the discretization in composition is in intervals of 5 wt %. The “before” and “after” changes to the observed phase counts on performing this L_1_ → L_2_ relabeling operation are reported in Table 1, as already mentioned. For completeness, we show in the Supporting Information, how the performance of the ML methods is degraded when this relabeling is not done, due to the superficial overweighting of the L_1_ phase. The hyperparameters used in this case are those of determined in the *de novo* challenge (described later in the text).

**Table 2:** Azeotropes Used for L_1_ → L_2_ Relabeling

surfactant	weight frac	temp/°C	phase
C_8_E_6_	0.54	13	H_1_
C_10_E_6_	0.53	37	H_1_
C_12_E_8_	0.52	57	H_1_
C_12_E_8_Me	0.51	43	H_1_
C_16_E_12_	0.54	88	H_1_
C_12_G(E_4_Me)_2_	0.64	2	H_1_
C_14_G(E_4_Me)_2_	0.57	33	H_1_
C_10_E_5_	0.74	31	L_α_
C_12_E_6_	0.68	73	L_α_
C_12_E_6_Me	0.63	42	L_α_
(C_6_)_2_GE_8_Me	0.57	18	L_α_

Further to above, in developing a data set describing the phase behavior of surfactants some pragmatic decisions need to be taken. For example, where ambiguity exists as to a specific phase, decisions must be taken on what to include in the training data (e. g., exclusion of the V_2_ phase of C_16_E_4_). If not included, then an alternative phase needs to be chosen. We note that these choices of simplification during the data cleaning stage may form sources of bias inherent to the data set, but are to some extent unavoidable.

We provide the derived data set used in this work, as Supporting Information, in machine readable format, to aid future work.

### 3.3 Molecular Descriptors

The data points from all the surfactants contained in the training set were collected together to form one data set consisting of **x**(*S*_*f*_; *w*, *T*) and *y*(**x**) . Each surfactant is described by four molecular descriptors: the tail length (*L*), tail volume (*V*), the headgroup area (*A*), and the derived critical packing parameter CPP = *AL*/*V*. For the present study we used the calculated values reported by Bell,^26^ thus each data point was described as **x**(*L*, *V*, *A*, CPP; *w*, *T*) . In this, we go beyond Bell (who used only CPP). The inclusion of more molecular descriptors reduces the dangers of degeneracy caused by surfactants sharing the same CPP value, but since the CPP descriptor is derived from the other three, it is arguably superfluous. We note though that the CPP has a long pedigree for being predictive of surfactant self-assembly and to omit it could amount to disregarding important prior knowledge.^51^ On the other hand, Nagarajan has argued that the CPP on its own is too crude and misses subtleties when it comes to packing considerations.^52^ We did therefore investigate the effect of omitting the CPP descriptor, but found that it makes no noticeable difference to the results. Thus, although we do include CPP as a fourth descriptor in the present study, there is no clear added benefit to doing so.

We chose not to include the surfactant name (such as the SMILES string) as it provides only limited chemical information, is not necessarily unique and has difficulty capturing features such as chirality. Later, however, we do explore the effect of adding a categorical “shape” descriptor (section 4.2.2 below).

### 3.4 Model Training

For each run the ML procedure was as follows: partition the data into a training set, validation set and test set; tune the hyperparameters on the validation set and then train the ML model using the training set; predict the test set using the model developed by the ML and assess the model performance of the prediction. We provide specific details on the final hyperparameters used for each of the eight ML classifiers (and for each of the surfactants in the *de novo* challenge) in the Supporting Information.

For the gap filling challenge the procedure outlined above was only run once as the data set partitioning was across the entire data set (not over individual phase diagrams). We randomly partitioned the data such that 30% of all the phase diagram points were in the test set, 14% were in the validation set and 56% were in the training set.

For the *de novo* prediction challenge the test was done for each of the 23 surfactants in turn, where for each case the test set was a single phase diagram while the validation set consisted of two phase diagrams and the training set was the remaining phase diagram data. This approach for the *de novo* surfactants is further refined in section 4.3 where the training set now encompasses additional data points from the *de novo* surfactant under investigation as if “new” experimental data had become available.

For both challenges, a Stratified Shuffle Split Algorithm (SSSA; with number of splits = 5) was used to split the validation and training sets after which an exhaustive cross-validation grid-search was used to find the set of hyperparameters that maximized the prediction accuracy on the validation set. The SSSA was chosen to try and alleviate issues involving data bias. The SSSA aims to maintain the percentage of each class (phase) within each fold, thereby ensuring classes with a very small number of samples are not missed in the random sampling process. We perform the search such that the hyperparameters are optimized for each test set used. The grid search ranges and optimal hyperparameters for each ML classifier used can be found in the Supporting Information.

### 3.5 Quality Metrics

To assess the quality of the phase diagram prediction, three metrics were used, namely,
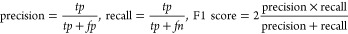
1where *tp*, *fp*, and *fn* are counts for the true positive, false positive, and false negative, respectively. For each phase state *C*, the above estimates were calculated and then averaged across *C* using two different weightings prefixed as macro or weighted. The macro-average metrics are defined such that each phase state classification is evenly represented in the average via
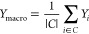
2where *Y*_*i*_ is the specific metric calculated for the given classification *i* ∈ *C*. The weighted-average metrics adjust the overall value by the fraction of each class in the test set, using
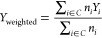
3where *n*_*i*_ is the number of examples of a given class in the test set. By considering both the macro and weighted metrics, we are able to determine how bias in a data set affects the results; if the weighted metrics outperform the macro metrics, this provides evidence that the model has higher performance on predicting the phase state for classes that correspond to more data points (“majority classes”) and has lower performance for smaller classes.

## 4 Results and Discussion

We report on the ability of our two chosen ML challenges (gap filling and *de novo*) to predict points on the phase diagram. We discuss potential issues present in the data and features used that may effect the resultant ML performance before finally discussing use of new experimental data points to improve the *de novo* diagram prediction.

### 4.1 Phase Behavior Prediction

Here we discuss the results of predicting the phase behavior of surfactants via the gap filling and *de novo* approaches as described in the Introduction.

#### 4.1.1 Gap Filling Challenge

**Figure 3:**
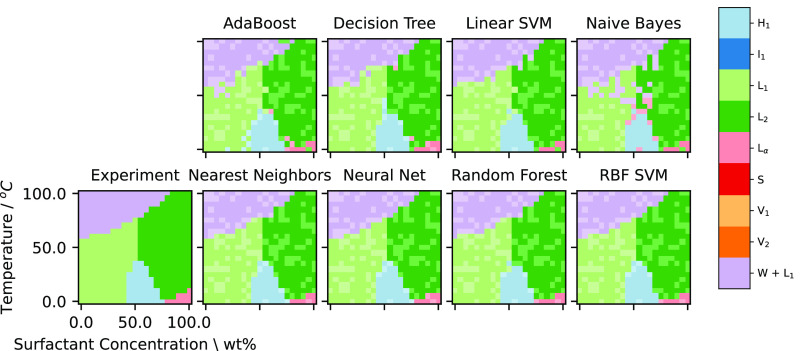
Results using different classification methodologies for the gap filling challenge for the phase diagram, for C_10_E_6_. The points included in the training data are shown using opaque colors, whereas the predictions are shown as slightly translucent.

Figure 3 gives representative examples, for C_10_E_6_ (in this case), of the predicted diagram produced by gap filling (other examples are given in the Supporting Information, and follow the same observation as given here). All the predicted phase diagrams look somewhat reasonable. This is because much of the data in the phase diagram exists in the training set and so each of the classifiers only need to interpolate the data from the rest of the known phase diagram, with knowledge of other partially complete surfactant phase diagrams.

**Figure 4:**
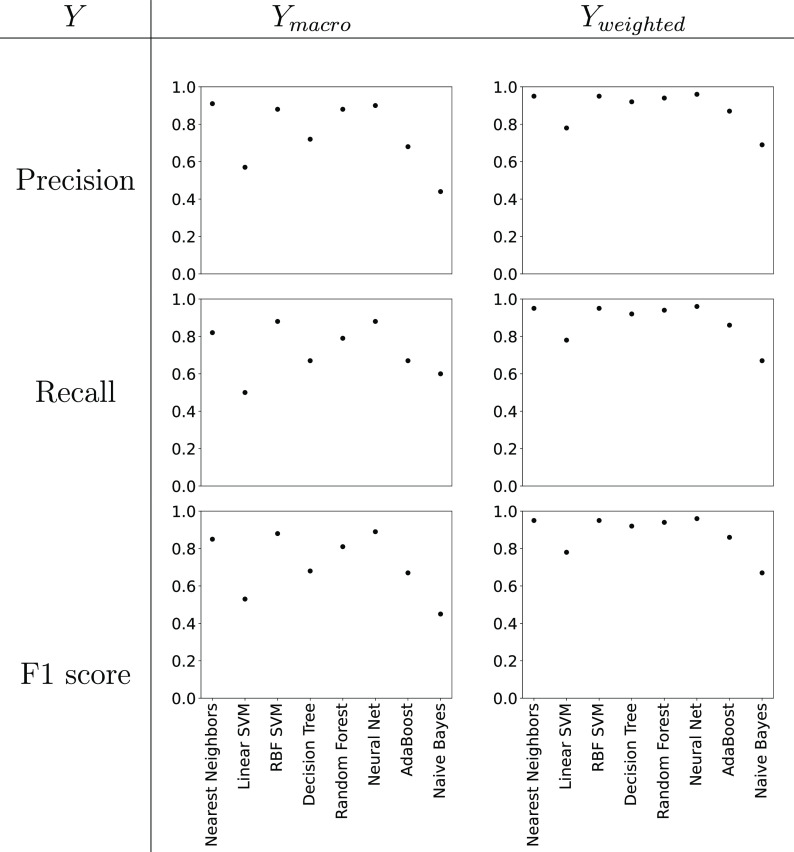
Plots giving the performance of different ML classifiers for the gap filling challenge.

For the predictions over the test set (i.e., gathered across the 23 simultaneously “filled-in” phase diagrams) we computed the *Y*_macro_ and *Y*_weighted_ for *Y* being the Precision, Recall, and F1 scores. The results for each of the classification methods are shown in Figure 4. A high value for *Y* close to 1 indicates good performance, while low value close to 0 represents poor performance.

Examples of well-performing classification methods are (listing the Precision_macro_, Recall_macro_, and F1_macro_, respectively, in brackets): Neural Networks (0.90, 0.88, 0.89), RBF SVM (0.88, 0.88, 0.88), and Nearest Neighbors (0.91, 0.82, 0.85). The ML classifier used by Bell^26^ was Decision Trees, which here gives scores of (0.72, 0.67, 0.68). In general, we find that *Y*_weighted_ is greater than the corresponding *Y*_macro_.

#### 4.1.2 *De Novo* Diagram Prediction

**Figure 5:**
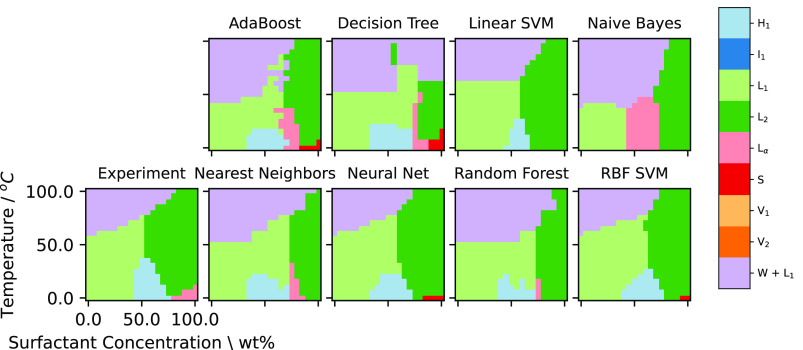
Results of using different classification methodologies for the *de novo* phase diagram challenge, for C_10_E_6_.

Figure 5 gives representative examples of *de novo* predicted diagrams (other examples are given in the Supporting Information and follow the same observation as given here). Each of these methods can capture the general motif of the phase diagram. Interestingly, the phase diagram generated by the Nearest Neighbors algorithm closely resembles the phase diagram for C_10_E_5_. This is expected as this is the nearest neighbor of the molecule C_10_E_6_ with respect to the surfactant features.

**Figure 6:**
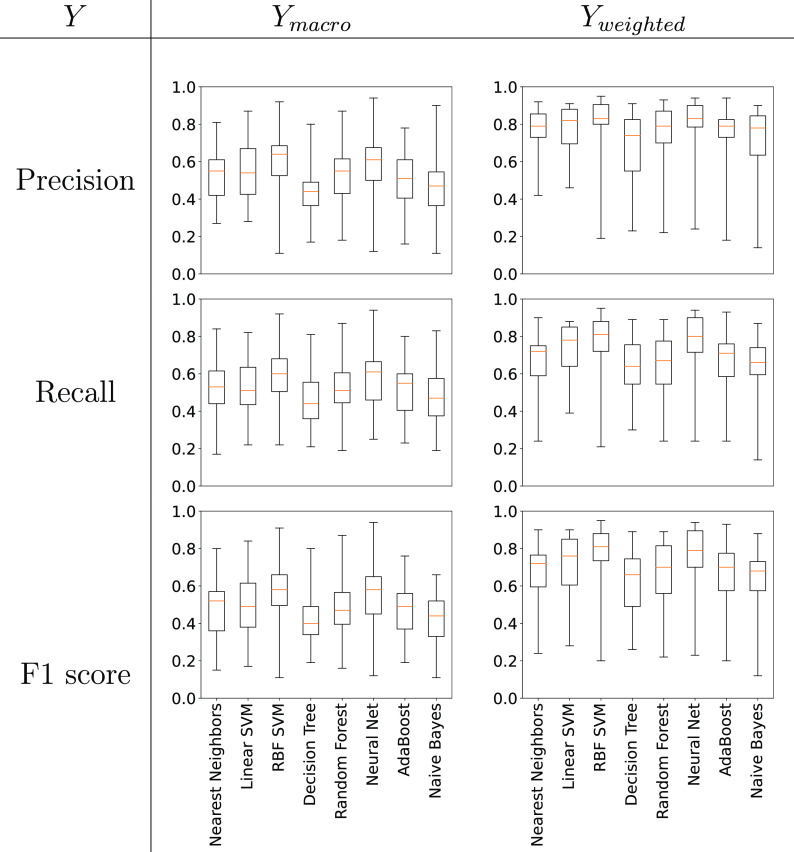
Box-whisker plots giving the performance of different ML algorithms for the *de novo* challenge (averaged across 23 phase diagrams).

Based on the predictions of the 23 test sets (i.e., each of the 23 separately predicted whole phase diagrams) we computed values of *Y*_macro_ and *Y*_weighted_ for *Y* being the Precision, Recall, and F1 scores. The distribution of results for each of the classification methods are shown in Figure 6. From the figure we see that the median value (across classification methods) for *Y*_macro_ is lower than *Y*_weighted_.

For example, we see for the Linear SVM algorithm a median macro value of 0.54, 0.51, and 0.49 vs a median weighted value of 0.82, 0.78, and 0.76 for the Precision, Recall, and F1 scores, respectively. As *Y*_weighted_ is weighted by the occurrence of each phase, a discrepancy between the *Y*_macro_ and *Y*_weighted_ values indicates that phases with fewer examples in the training set (such as V_1_ or V_2_) are less accurately predicted.

#### 4.1.3 Performance Contrast between the Two Challenges

From the above data we have found that the ML performs much better at gap filling than predicting phase diagrams *de novo*. This is seen when comparing Figure 6 to Figure 4, where we note that the average Precision, Recall, and F1 metrics all indicate better results for gap filling. The reason for this is self-evident: when predicting an entirely new phase diagram, the ML classification models must interpolate not only across the known phase diagrams, but also across chemical space (i.e., between surfactants, which is challenging due to the increased complexity of the feature space), but when predicting missing parts of a phase diagram, the models need only interpolate across the phase diagrams (a simpler task as the data is contiguous and classification regions are localized). In fact, it seems to us that a nonexpert with a basic knowledge of phase diagrams could rather easily do the interpolation (given the training data here) but would struggle to predict an entirely new phase diagram for a *de novo* surfactant.

For the gap filling challenge we see that Neural Networks are one of the best performing ML classifiers alongside RBF SVM and Nearest Neighbors (see Figure 4). For *de novo* prediction, we find that RBF SVM and Neural Networks generally perform well, however, Nearest Neighbors is no longer in the top three performing classification methods (see Figure 6). This drop in performance of Nearest Neighbors compared to the other classification algorithms in the *de novo* challenge could be indicative of the fact that predicting the entire phase diagram is much harder than making gap filling predictions, as in the former phases can no longer be inferred from nearby points on the same phase diagram. Furthermore, given that Nearest Neighbors performs worse when rank-ordering the ML classifiers for *de novo* prediction compared to gap filling (i.e., in Figure 6 versus Figure 4) indicates that different ML methods are more viable for surfactant phase diagram prediction, depending on how the data is split.

### 4.2 Issues of Data Quality

As discussed in section 4.1.2 with respect to Figure 6, it was observed that the weighted-average metrics (Precision, Recall, and F1 scores) outperformed the macro-average metrics. In this section, we will discuss data bias in the data set further and the distribution of the data set in chemical space.

#### 4.2.1 Degree of Imbalance of Phases in the Data Set

**Figure 7:**
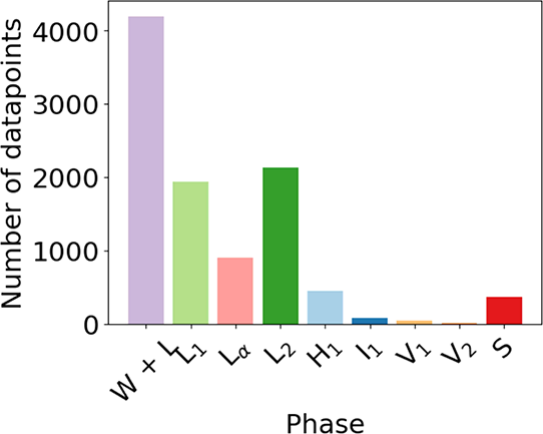
Number distribution of the phases in the data set (these correspond to the final row in Table 1).

Figure 7, shows the number of occurrences of each classification *C* in our data set *y*(**x**) . Class imbalance data bias exists in the classification data sets when the distribution of *C* is far from uniform, i.e., the number of occurrences of each classification *C* in *y*(**x**) are not close to being equal. We find that the W + L phase coexistence region, and the L_1_ and L_2_ phases are over-represented compared to other phases, whereas the V_1_, V_2_, and I_1_ phases are under-represented. Hence, it is apparent in Figure 7 that there is a degree of class imbalance to our data sets.

**Figure 8:**
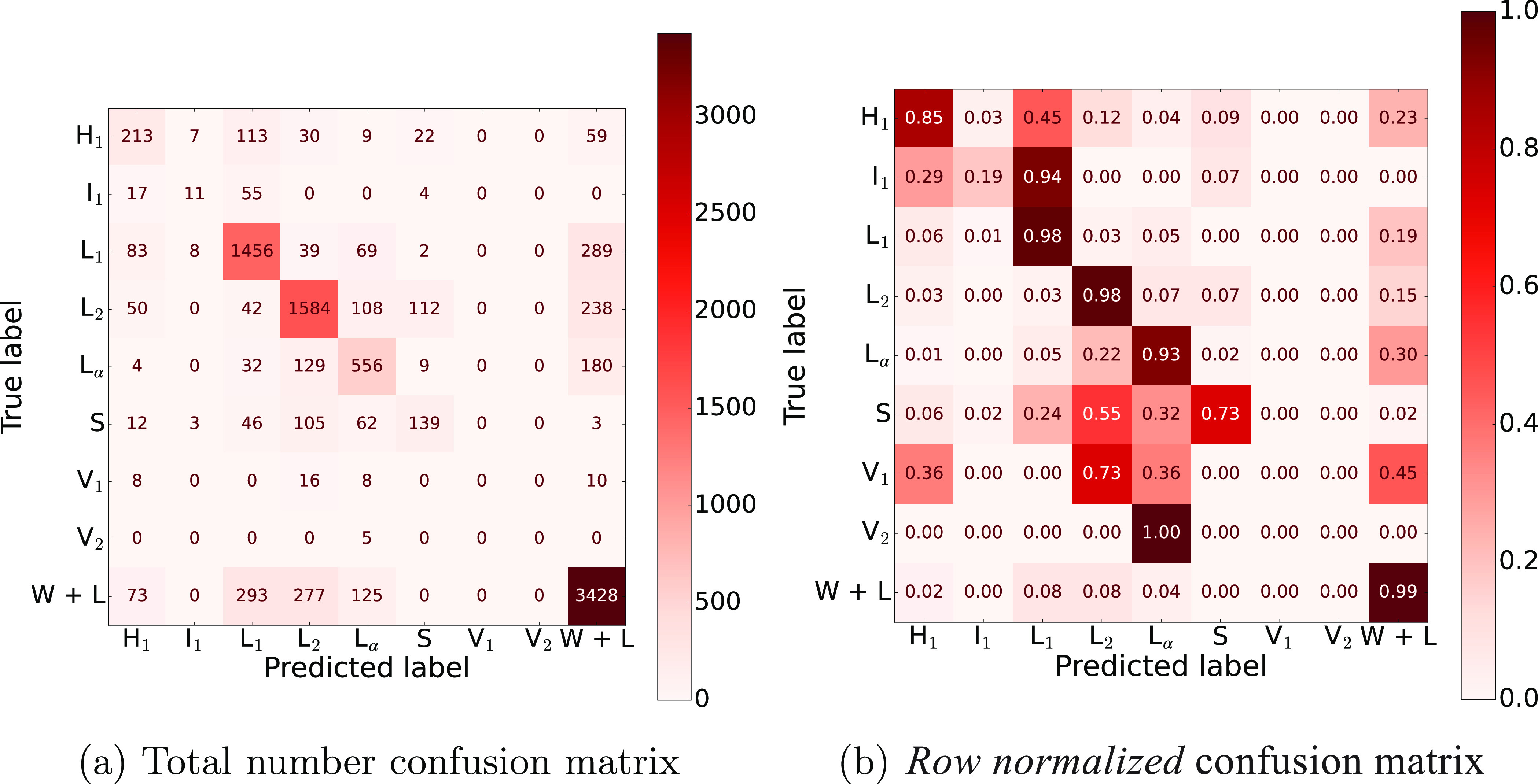
Confusion matrices for the RBF SVM, obtained from all the surfactants.

The effect on the prediction of having over-represented classes can be illustrated using *confusion matrices*. Figure 8a shows an example for the ML approach RBF SVM (see Supporting Information, for other examples). In the plot, the diagonal entries give the number of correct predictions made by the model and the off-diagonal entries the number of incorrect predictions. In Figure 8b we renormalize the data across each row so that differences in false predictions of the rare phases (i.e., V_1_, V_2_, I_1_, and S) can easier be seen.

From Figure 8 it is evident that the chance of a correct prediction being made is correlated with the (over)representation of the phase in the data set, i.e., the larger the occurrence of class *C* in *y*(**x**) the higher diagonal entry in the confusion matrix. Furthermore, false predictions are skewed to these phases, for example, a large proportion of the actual V_1_ and V_2_ phases are (mis-)identified as L_2_ and W + L, respectively.

Data bias is a common phenomenon in ML tasks. Indeed, data bias is already known to exist in chemical data sets. For example, it has been shown to exist in chemical data used in structure-based virtual screening by Sieg et al.^53^ The data bias discussed by these authors also extends to discussions of “analogue bias”,^54^ whereby data sets over-represent similar molecules with the same chemotype or chemical scaffold; “false negative bias”,^55^ where a chemical data set is enriched with nonvalidated molecules assumed to be inactive, but once validated, are actually active; and “confirmation bias”,^56^ where a human searches for data to explicitly confirm a hypothesis in the process finds fortuitous correlation. Though each of these types of data bias are important to consider, it should be noted that they are not the same as the data bias being discussed in this paper.

In the current Article we do not address class imbalance any further than through the already described method to train the hyperparameters and by relabeling some of the original data from L_1_ → L_2_. However, in extending the present work, one may consider adopting under/oversampling methods or cost sensitive learning (for example). To achieve the best results, one would need to consider the ML classifier being used (as different approaches to addressing class imbalance may apply to a specific method) and the question being asked by the ML study (i.e., a formulator may be concerned about predicting one specific phase as accurately as possible, e.g., to understand the location of a highly viscous H_1_ phase, while not being concerned about other marginal, e.g., V_2_ phase).

#### 4.2.2 Data Sparsity of Surfactants

**Figure 9:**
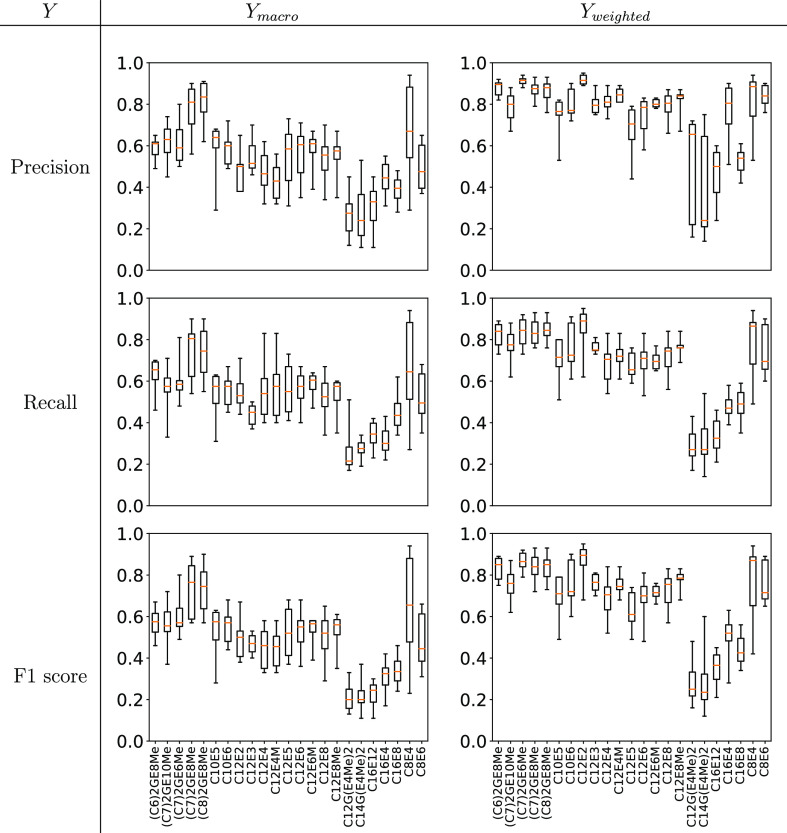
Box-whisker plots giving the performance of the *de novo* challenge for different surfactants averaged over all ML classifiers.

In Figure 9 we breakdown the overall Precision, Recall, and F1 scores (averaged over all classification methods) into the contributions for each surfactant; this allows us to analyze the performance of each surfactant individually. From Figure 9 it is evident that the worst performing surfactants are from the C_16_E_*m*_ and C_*n*_G(E_4_Me)_2_ families.

Why might this be so? Each of the surfactants are characterized by the four chosen molecular descriptors. To understand how the chemical space is represented by these descriptors in the data set we analyze how each of the surfactant types cluster using *t*-distributed neighbor embedding (t-SNE).^57^ This method allows for the visualization of high-dimensional (here, four-dimensional) data by embedding it within a two-dimensional space. In this case we initialized the t-SNE plots such using principle component analysis and set the t-SNE perplexity parameter to 5. We only applied t-SNE to the chemical space features (discussed in section 3.3).

**Figure 10:**
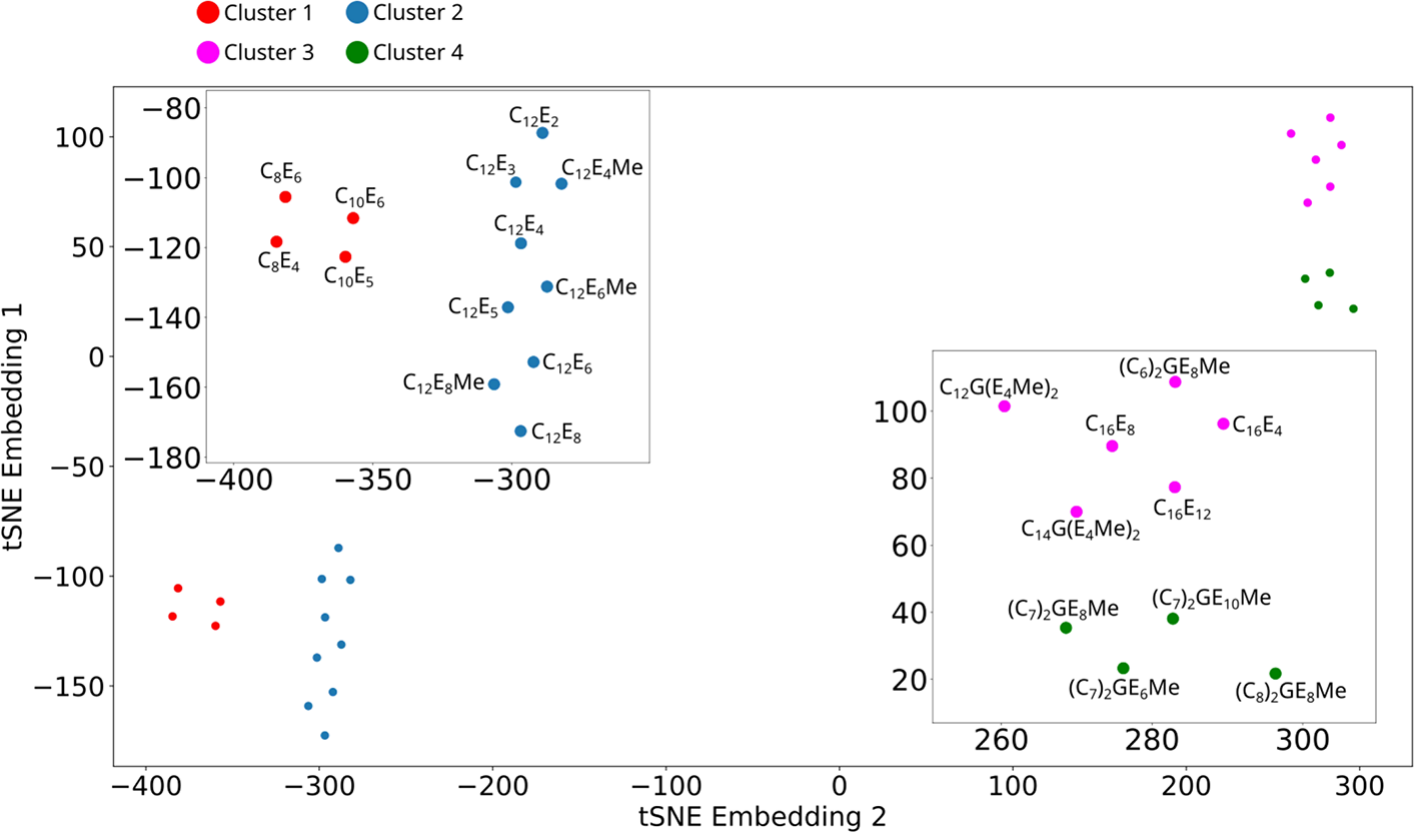
Plot showing the t-SNE of molecular features indicating the four clusters with insets giving enlarged views labeled by surfactant *S*_*f*_.

The results are shown in Figure 10 where four distinct clusters of points emerge: the first cluster contains the surfactants C_8_E_*m*_ and C_10_E_*m*_; the second contains C_12_E_*m*_ and C_12_E_*m*_Me surfactant families; the third contains the C_16_E_*m*_ and C_*n*_G(E_4_Me)_2_ surfactant families; and the fourth contains the (C_*n*_)_2_GE_*m*_Me surfactant families. We note that poorest performing surfactants are all in cluster 3 of the t-SNE plot Figure 10.

**Figure 11:**
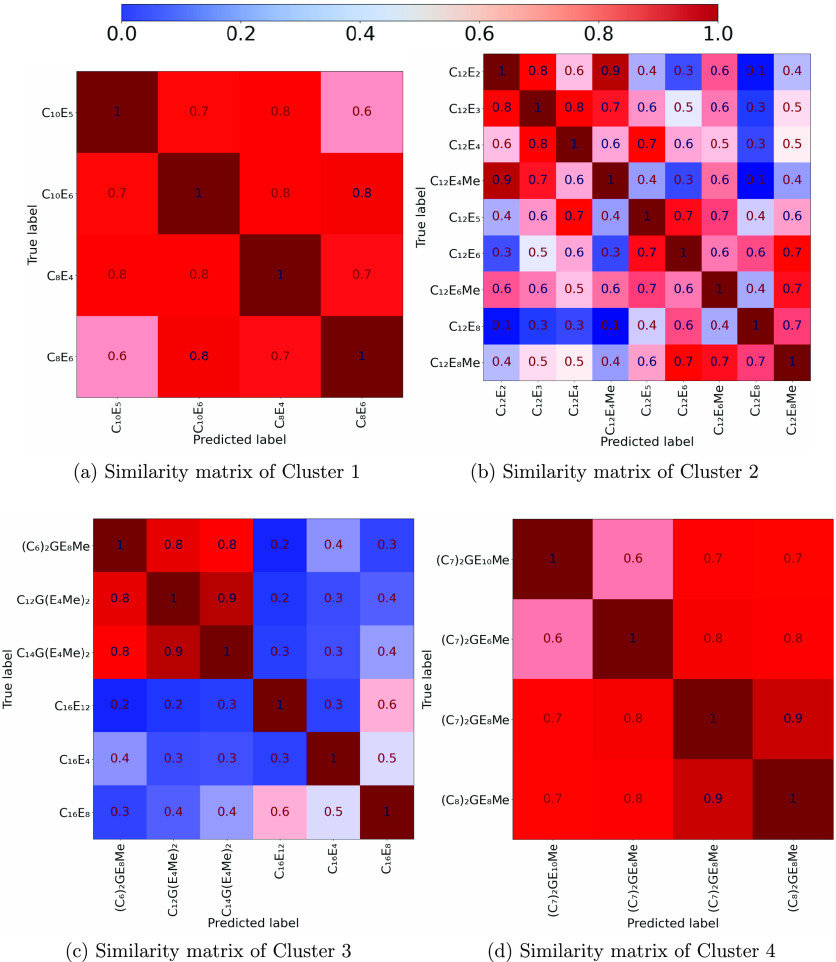
Matrices showing the similarity between each of the phase diagrams in the clusters highlighted in Figure 10.

Good predictions occur when a cluster contains similar phase diagrams (i.e., when it is reasonable for the ML to learn from the other diagrams in the cluster to make a prediction about another member). Conversely, poorer predictions will occur when the members of a cluster have dissimilar diagrams (an indication that too few features may be used). To judge whether sufficient clustering had occurred we calculated the similarity matrix for each cluster (see Figure 11), where each element of the matrix gives, for the two surfactants being compared, the fraction of state points on the phase diagram that have the same phase. Phase diagrams with high similarity score toward a maximum of 1 (that being a pair of identical diagrams) and low similarity score toward 0 (that being completely dissimilar diagrams). It can be seen that there is poor similarity between the C_16_E_*m*_ and C_*n*_G(E_4_Me)_2_ phase diagrams (see low values in Figure 11c). This indicates that despite being similar in feature space (i.e., in the same cluster), the two groups do not have similar phase diagrams. By contrast, C_10_E_*m*_ and C_8_E_*m*_ (Figure 11a) show high similarity and much better goodness scores in Figure 9.

We order the similarity matrices in Figure 11 such that surfactant molecules with similar phase diagrams are located near to each other (this was achieved using a genetic algorithm, from Hayes,^58^ to reorder the rows and columns of the matrices to minimize the difference in the value of neighboring elements). After this we can identify subclusters lurking within the poorly represented cluster (i.e., cluster 3 shows that C_*n*_G(E_4_Me)_2_ are highly similar to each other, while to a lesser extent cluster 2 could be subdivided into shorter, C_12_ ethoxylates less than E_5_, and longer length members). We can therefore postulate that the poor performance of these groups is due to the inadequacy of the chosen features to fully describe differences in the phase diagrams. Additionally, despite being similar in the feature space, none of the C_16_E_*m*_ surfactants have particularly similar phase diagrams (with a maximum similarity score of 0.6 between C_16_E_12_ and C_16_E_8_). For example C_16_E_4_ has a region classified as “S” below the Krafft boundary that is concentration independent at moderate temperatures (the only diagram to do so), C_16_E_8_ has a broad L_α_ phase spanning to high temperatures. This, again, indicates that the chemical space features are inadequate to encode the differences between phase diagrams in this group.

**Figure 12:**
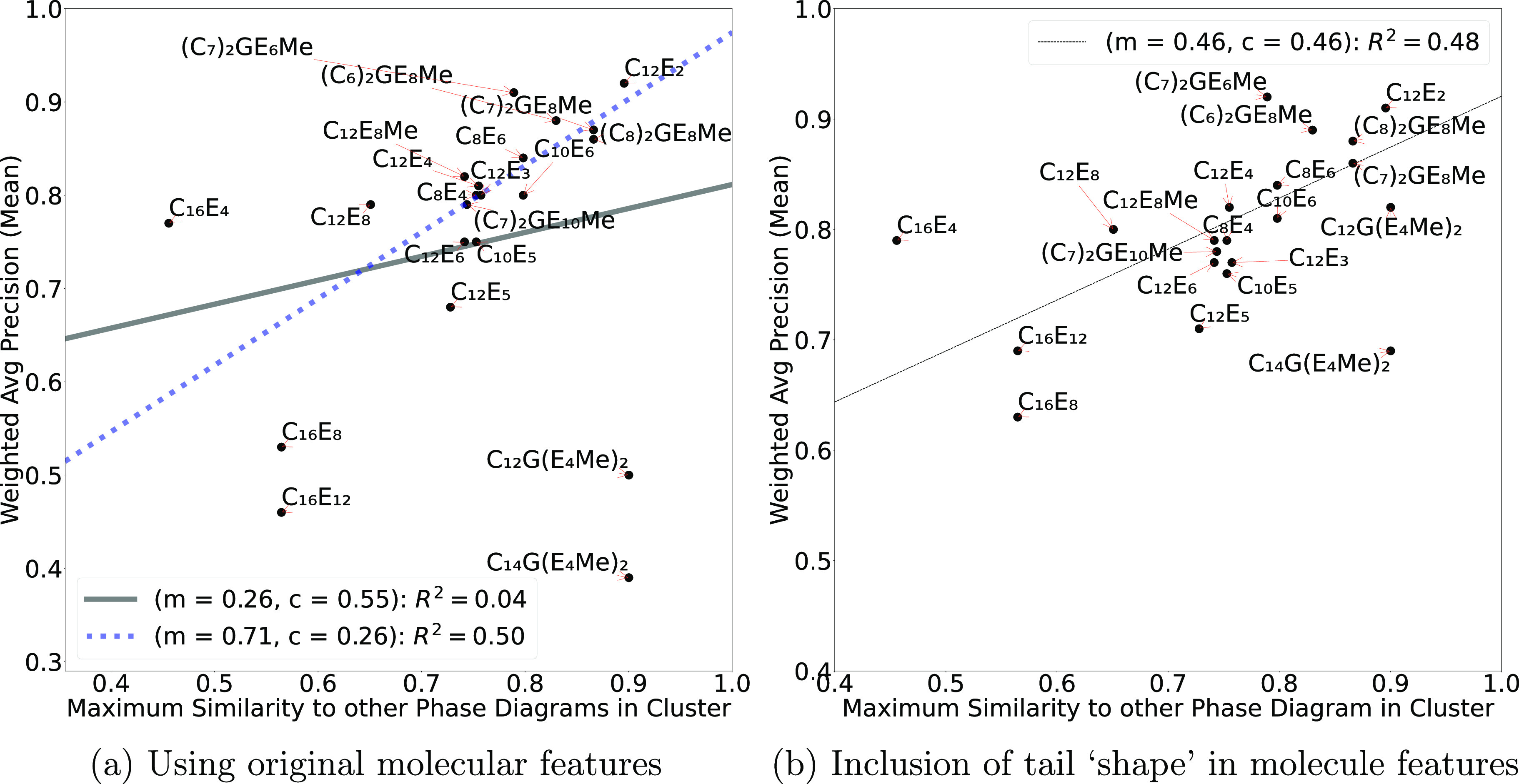
Degree of correlation between the maximum similarity to other phase diagrams in the surfactant’s cluster and the quality of prediction. The legend and line shows the least-squares linear fit (of form *y* = *mx* + *c*) and its *R*^2^ goodness value. Solid line includes all surfactants and the dotted line excludes surfactants from cluster 3 (see Figure 11).

To understand this further, for each surfactant we compared the obtained aggregated *Y*_weighted_ (Recall, Precision, and F1 scores averaged over all the ML methods) for the *de novo* prediction of its phase diagram against the maximum similarity the diagram is to another in the cluster containing the surfactant (i.e., the maximum column value of the surfactant in the relevant similarity matrix). If we have clustered the surfactants well enough then the degree to which the surfactant diagram is similar to another phase diagram in the cluster should correlate with the performance of the prediction (i.e., a high maximum in similarity should correlate to a high value for *Y*_weighted_) such that there is a linear correlation of the form *y* = *mx* + *c*, where *x* and *c* are constants. The comparison for Precision are shown in Figure 12a and for Recall and F1 scores in Supporting Information. We can see in Figure 12a that the linear fit is poor with *R*^2^ = 0.04, 0.16, and 0.17 for plots of Precision, Recall, and F1 score, respectively, where the fit is degraded by the inclusion of the outlier surfactants contained in cluster 3. Here we find the C_*n*_G(E_4_Me)_2_ family of surfactants are predicted poorly, despite having a high maximum similarity, while the C_16_E_*m*_ show low maximum similarity and have mixed predictive performance. To test this we performed a second fit where we excluded these surfactants from cluster 3 and achieved a much better *R*^2^ value of 0.50.

**Figure 13:**
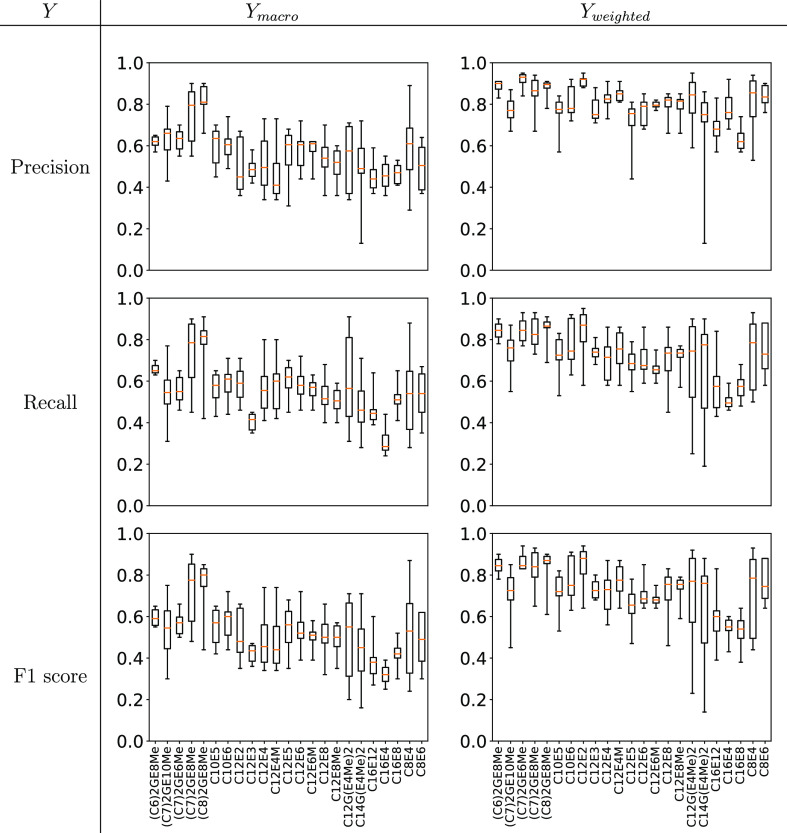
Box-whisker plots giving the performance of the *de novo* challenge for different surfactants averaged over all ML classifiers when including the tail “shape” descriptor as part of the molecular features.

So, how might we improve the predictions? We note that an intuitive feature missing from the molecular descriptors is the “shape” of the tail of the surfactant (either linear, V-shaped, or Y-shaped). Its inclusion may strongly affect cluster 3 as C_*n*_G(E_4_Me)_2_ and C_16_E_*m*_ are very different in shape, despite being similar in the molecular descriptors used so far. To verify our hypothesis that the tail shape descriptor has an impact prediction of phase diagrams, we retrained the classification algorithms using this expanded molecular feature set. Figure 12b plots the correlation between the maximum phase diagram similarity (to another within the cluster) and performance of the predicted diagram, for the Precision (Figure 12b), Recall, and F1 score, when using the expanded feature set. We can see from in Figure 12b that the linear fit is much better with *R*^2^ = 0.48, 0.87, and 0.82 for plots of Precision, Recall, and F1 score, respectively, a marked improvement over the original goodness values. Similarly, when looking directly at the box-whisker plots giving the performance of *de novo* for different surfactants averaged over all ML classifiers (Figure 13) we see that by including a description of tail shape as part of the molecular features, the phase diagram predictions of the C_*n*_G(E_4_Me)_2_ family of surfactants are improved.

### 4.3 Improving *De Novo* Prediction with Laboratory Sampling Strategies

In the *de novo* challenge presented earlier, we attempted to predict an entire phase diagram based on data from prior sampled phase diagrams. This has shown to be a challenge to the ML classifiers so far. Here we build upon the *de novo* approach to determine how laboratory efforts may be maximized to improve the predictions in an efficient manner. We consider three sampling strategies which are aligned to what might be encountered in a laboratory setting: fine-grained sampling in composition, at selected temperatures, to mimic data that might be obtained by performing quantitative penetration scan experiments (A1); fine-grained sampling in temperature, at selected compositions, to mimic data that might be obtained from hot-stage microscopy measurements (A2); grid-based sampling, to mimic data that might be generated using an experimental design (A3). Note that this sparse grid sampling can be regarded as a “special” case of other two methods, where significantly less training data is available. See the Supporting Information for a pictorial representation of these three scenarios.

For the first approach (A1), we fix the temperatures at 10, 30, 50, 70, and 90 °C and use the entirety of the available concentration data available for each of the sampled temperatures of the studied surfactant as training data. For the second (A2), we fix the concentrations at 10, 30, 50, 70, and 90 wt % and use all available temperature data at these sampled concentrations for training. In both these approaches, therefore, a total of 21 × 5 = 105 data points are used as training data, out of a total of 21^2^ = 441 data points available for the surfactant sampled. For the third approach (A3), we sample a grid of 5^2^ = 25 points corresponding to intervals of 20 units starting at 10 °C and 10 wt % going up to 90 °C and 90 wt % In all approaches the unsampled points on the selected phase diagrams are considered as test points. The phase diagrams of surfactants not being actively sampled all contribute to the training set in their entirety. Here we used the hyperparameters trained for the *de novo* prediction of the surfactant in question.

**Figure 14:**
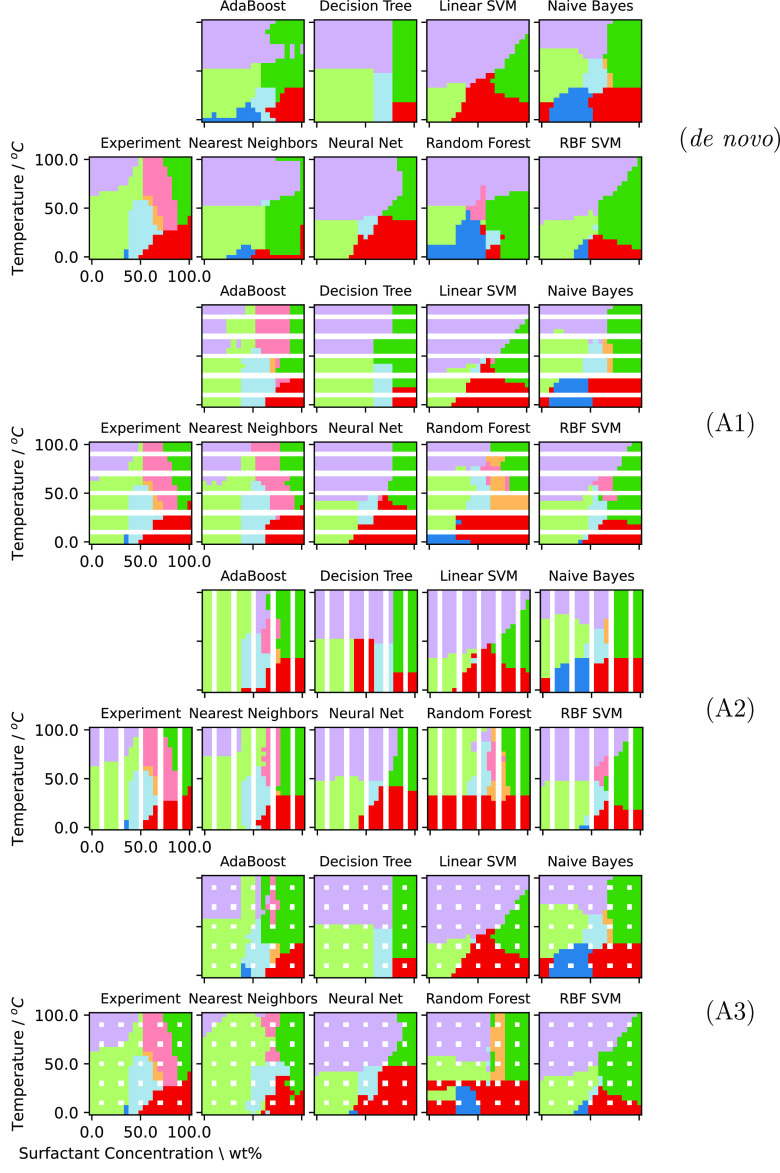
Predicted phase behavior for C_16_E_8_. Each row corresponds to a different sampling strategy: the top row is the *de novo* result; the second row is A1; the third row is A2; and the bottom row is A3, such as might be generated in an experimental design. Training data is blanked out. Colors are the same as Figures 3 and 5.

**Figure 15:**
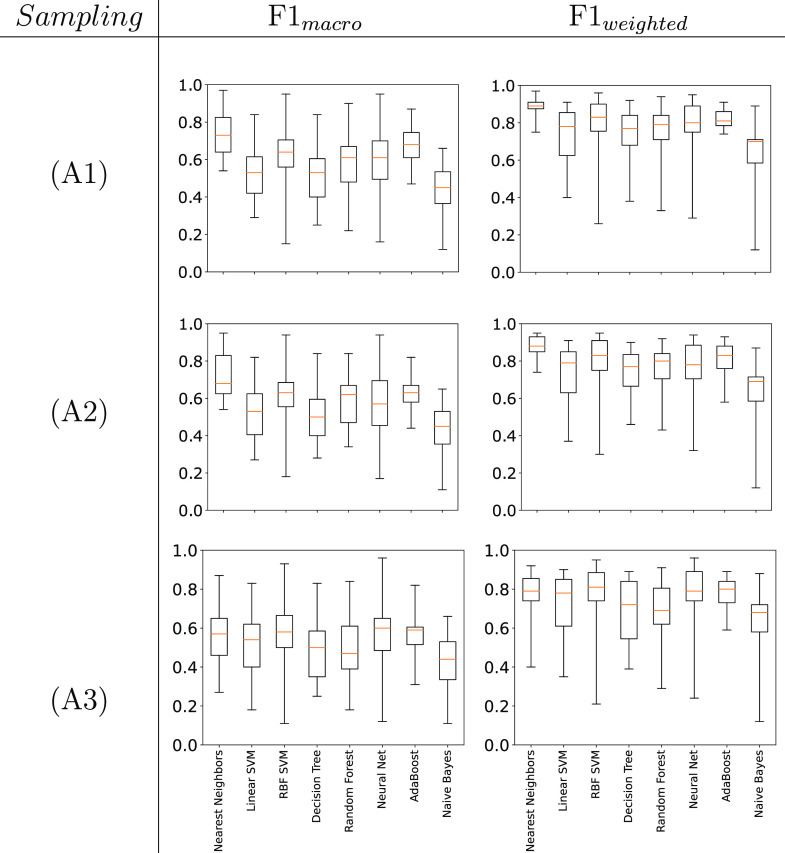
Box-whisker plots giving the performance (F1 score) of different ML algorithms for the laboratory sampling challenges averaged across all 23 surfactants.

Figure 14 shows results for the predicted phase diagram of C_16_E_8_, chosen for its rich phase behavior and to challenge the ML algorithms since otherwise this is a poorly predicted surfactant (see Figure 9). The C_12_E_5_ and (C_8_)_2_GE_8_Me surfactants are presented in the Supporting Information. Plots showing the F1 scores of the different ML classifiers against the scenarios described above are given in Figure 15 (while we show F1 scores in Figure 15 the same behaviors are consistent across Precision and Recall).

From the inspection of Figures 14 and Figure 15 and cross referencing to Figure 6, it can be seen that *de novo* prediction is improved when the additional training data provided by the “laboratory” samples is included. Here the AdaBoost and Nearest Neighbors algorithms combined with the penetration scan scenario (A1) appear, visually, to provide the best improvement for C_16_E_8_. For A1 both methods capture the major features of the phase diagram correctly. Notable exceptions are that both of these methods fail to capture the I_1_ behavior from experiment, and the Nearest Neighbors does not reproduce the V_1_ phase well. In general, the other ML methods produce phase diagrams that poorly represent the experimental situation.

For A2, Nearest Neighbors and AdaBoost again appear, visually, to outperform the other ML classifiers and the I_1_ phase is still not present. Prediction of the W + L phase coexistence region is less accurate with A2 sampling but V_1_ phase prediction appears to be slightly better than produced by A1 sampling. Once again, the other ML classifiers result in poor predictions. Finally, utilizing a sparse grid for training data (A3) unsurprisingly performs the worst of the three sampling approaches offering only a slight improvement over the original *de novo* predictions. However, the AdaBoost method combined with A3 does manage to capture the I_1_ behavior well where A1 and A2 scenarios fail for this phase.

From Figure 15 we note that the addition of more data from higher levels of sampling (i.e., in A1 and A2) results in the Nearest Neighbors method improving above other ML classifiers. This we attribute to the fact that the algorithm can now interpolate within the phase diagram being sampled rather than having to predict solely from molecular features.

## 5 Conclusion

In this article we have examined the capability of different ML classifiers to predict the liquid phase behavior of surfactants. We tested this capacity in two different contexts. The first was the ability to “gap fill” points over phase diagrams for which significant data is already known. This is similar to the study presented by Bell,^26^ and our findings are in line with this previously reported work. The second more challenging problem is the use of ML to predict the phase diagram of a *de novo* surfactant based on the known phase behavior of other surfactants in the same class.

Unsurprisingly, this latter problem is a much harder task for the ML classifiers and the quality of the predictions are poorer than for the gap filling problem, although a subset of the ML classifiers do capture the broad behaviors one would expect (i.e., we achieve approximately 50–90% Recall (macro) for gap filling and ≈50% Recall for the *de novo* approach). Through our studies, we find that the classifiers most likely to give good results are dependent upon the challenge posed. For the gap filling challenge we observe Neural Networks as one of the top performing classifiers along with RBF SVM and Nearest Neighbors (see Figure 4). For *de novo* prediction observe that RBF SVM and Neural Networks perform well in general, however Nearest Neighbors is no longer in the top three performing classifiers (see Figure 6). This apparent reduction in performance of Nearest Neighbors compared to the other classifiers in the *de novo* case may stem from the fact that predicting full phase diagrams is much more difficult than the gap filling predictions. In the *de novo* case, phases can no longer be inferred from nearby points on the same phase diagram. Our observations in section 4.3 support this idea. As we begin to add data to a previously unknown phase diagram, the Nearest Neighbors algorithm improves in predictive performance considerably.

We also explored models for different experimental sampling strategies. All strategies improved the quality of the *de novo* predictions with the benefit of performing penetration scan and hot stage microscopy type measurements being approximately equal when factoring in all surfactant molecules. Both of these sampling strategies outperform the sparse grid type sampling strategy, but this is perhaps not surprising as this strategy uses less data in training the ML model.

From our study we now have enough results to answer the titular question of the present study: *Can machine learning predict the phase behavior of surfactants?* With the data set used (which is the most comprehensive openly available one for this type of study to the best of our knowledge), the answer is “yes”, but the predictions can be improved. We have identified three areas in which data provided to the ML classifiers warrants further development, namely, improving the data bias in surfactant phases observed, building better training data with respect to the chemical space (i.e., different surfactants) and providing more comprehensive feature space (i.e., relevant chemical characteristics).

On a conceptual level the ability of the ML classifier to learn the phase diagram comes down to its ability to cluster “similar” phase diagrams into distinct groups via the commonality of the surfactant’s features. If this fails then the prediction becomes poor as it is based on knowledge gained from dissimilar diagrams. Hence, improving the performance of the ML prediction is coupled to the above three issues and could be tackled as follows.

- The data bias becomes a factor when the similarity metric used for the cluster (and loss function) becomes dominated by highly occurring phases, such that differences in less frequent phases are suppressed. This results in the ML getting the larger phases correct at the expense of the smaller phases (that are often more industrially important). Hence, a better choice in these metrics would reduce this distortion.
- With a good similarity metric, then increasing cluster similarity should improve the goodness of the prediction and can be achieved by better choice and/or additional features, beyond just those associated with the critical packing parameter (CPP), which further subdivide the clusters (e.g., by separating twin tail surfactants from single tailed). Poor similarity within the cluster results in the ML hybridizing the predictions of members of the cluster that poorly represent any.
- Finally, adding additional points to the cluster (by measuring more surfactants) would improve the quality of the prediction for the cluster by increasing the size of the training set.

If these issues can be overcome, then the outlook is optimistic that the predictions will become more reliable.

We believe that for ML to tackle the problem of surfactant phase diagram prediction in the future, the current data sets would have to be greatly expanded. Although the data set used in this work has a significant number of individual data points, it lacks data from a large variety of individual surfactants, and hence, the data set is very limited with respect to chemical space. A useful analogy here is that of digital images, where the surfactant is each individual image and the points on the associated phase diagram are the pixels. It is evident that even a small number of images could produce a large number of data points, but many of these would be related to one another (e.g., not independent samples). Another consideration is the distribution of the data points in the data set. In some methods, such as Support Vector Machines, the majority of the data points in the training data set are ignored and only those that define boundaries are used (hence, the term *support vector*). This means that even though there may be a large number of data points, the actual amount of useful information can be considered small as only points local to the *phase boundaries* are relevant and the rest is ignored.

When building new data sets, it would be important to look toward reducing data bias and efficiently gathering data for currently under-represented regions of chemical space. The generation of such surfactant phase diagram data is no trivial task. Molecular simulation methods are generally still too inaccurate for reliable data generation and manual experiments are time-consuming, repetitive and expensive. However, to minimize the effort and cost associated with experimental measurements one could turn to recent developments in efficient sampling of phase diagrams aided by machine learning methods such as those presented by Pyzer-Knapp and Anderson,^59^ Katsube et al.,^60^ Dai and Glotzer.^61^ Another hope is that the latest developments in high-throughput robots or mobile robotic chemists^19^ could potentially provide an approach to rapidly generate the required experimental data. These approaches, combined with more automated artificial intelligence and ML methods exploring automated data extraction from literature articles, have the capacity to build large data sets rapidly under the correct environments.

## Supporting Information Available

A machine readable format data set comprising the surfactants studied along with their phase behavior at specific temperature and composition (weight fraction) points. For each surfactant, the calculated tail length (Å), tail volume (Å^3^), and headgroup area (Å^2^ at 25 °C) are reported as given in Bell, along with the CPP value corresponding to these measurements.^26^ Details of the shape of the surfactant and reference containing the phase diagram are provided in the file (TXT)
Machine learning performance using unaltered phase state labels (corresponding to section 3.2); A detailed account of our approach to developing hyperparameters for the ML classifiers (corresponding to section 3.4); Gap filling predictions for the full set of surfactants (corresponding to section 4.1.1); Full phase diagram prediction for full set of surfactants (corresponding to section 4.1.2); Confusion plots for all studied surfactants (corresponding to section 4.2.1); Correlation between regression metrics and maximum similarity (corresponding to section 4.2.2); Further laboratory sampling predictions for additional surfactants (corresponding to section 4.3) (PDF)

## References

[ref1] Mohanty S.; Koul Y.; Varjani S.; Pandey A.; Ngo H. H.; Chang J.-S.; Wong J.; Bui X.-T. A Critical Review on Various Feedstocks As Sustainable Substrates for Biosurfactants Production: A Way Towards Cleaner Production. Microb. Cell Fact. 2021, 20, na 10.1186/s12934-021-01613-3. PMC8236176 34174898

[ref2] Saygin D.; Gielen D. Zero-Emission Pathway for the Global Chemical and Petrochemical Sector. Energies 2021, 14, 3772 10.3390/en14133772.

[ref3] Smit B.; Hilbers P. A. J.; Esselink K.; Rupert L. A. M.; van Os N. M.; Schlijper A. G. Computer Simulations of a Water/Oil Interface in the Presence of Micelles. Nature 1990, 348, 624–625. 10.1038/348624a0.

[ref4] Groot R. D.; Warren P. B. Dissipative Particle Dynamics: Bridging the Gap Between Atomistic and Mesoscopic Simulation. J. Chem. Phys. 1997, 107, 4423–4435. 10.1063/1.474784.

[ref5] Vishnyakov A.; Lee M.-T.; Neimark A. V. Prediction of the Critical Micelle Concentration of Nonionic Surfactants by Dissipative Particle Dynamics Simulations. J. Phys. Chem. Lett. 2013, 4, 797–802. 10.1021/jz400066k. 26281935

[ref6] Anderson R. L.; Bray D. J.; Ferrante A. S.; Noro M. G.; Stott I. P.; Warren P. B. Dissipative Particle Dynamics: Systematic Parametrization Using Water-Octanol Partition Coefficients. J. Chem. Phys. 2017, 147, 094503 10.1063/1.4992111. 28886630

[ref7] Anderson R. L.; Bray D. J.; Del Regno A.; Seaton M. A.; Ferrante A. S.; Warren P. B. Micelle Formation in Alkyl Sulfate Surfactants Using Dissipative Particle Dynamics. J. Chem. Theory Comput. 2018, 14, 2633–2643. 10.1021/acs.jctc.8b00075. 29570296

[ref8] Nivón-Ramírez D.; Reyes-García L. I.; Oviedo-Roa R.; Gómez-Balderas R.; Zuriaga-Monroy C.; Martínez-Magadán J.-M. Critical Micelle Concentration of SDS Through DPD Simulations Using COSMO-RS-Based Interaction Parameters, the Thermal Effects. Colloids Surf. A: Physicochem. and Eng. Asp. 2022, 645, 128867 10.1016/j.colsurfa.2022.128867.

[ref9] Panoukidou M.; Wand C. R.; Del Regno A.; Anderson R. L.; Carbone P. Constructing the Phase Diagram of Sodium Laurylethoxysulfate using Dissipative Particle Dynamics. J. Colloid Interface Sci. 2019, 557, 34–44. 10.1016/j.jcis.2019.08.091. 31514092

[ref10] Johnston M. A.; Duff A. I.; Anderson R. L.; Swope W. C. Model for the Simulation of the CnEm Nonionic Surfactant Family Derived from Recent Experimental Results. J. Phys. Chem. B 2020, 124, 9701–9721. 10.1021/acs.jpcb.0c06132. 32986421

[ref11] Del Regno A.; Warren P. B.; Bray D. J.; Anderson R. L. Critical Micelle Concentrations in Surfactant Mixtures and Blends by Simulation. J. Phys. Chem. B 2021, 125, 5983–5990. 10.1021/acs.jpcb.1c00893. 34043913

[ref12] Shelley J. C.; Shelley M. Y. Computer Simulation of Surfactant Solutions. Curr. Opin. Colloid Interface Sci. 2000, 5, 101–110. 10.1016/S1359-0294(00)00042-X.

[ref13] Taddese T.; Anderson R. L.; Bray D. J.; Warren P. B. Recent Advances in Particle-Based Simulation of Surfactants. Curr. Opin. Colloid Interface Sci. 2020, 48, 137–148. 10.1016/j.cocis.2020.04.001.

[ref14] Thacker J. C.; Wilson A. L.; Hughes Z. E.; Burn M. J.; Maxwell P. I.; Popelier P. L. Towards the Simulation of Biomolecules: Optimisation of Peptide-Capped Glycine Using FFLUX. Mol. Simul. 2018, 44, 881–890. 10.1080/08927022.2018.1431837.

[ref15] Hughes Z. E.; Ren E.; Thacker J. C.; Symons B. C.; Silva A. F.; Popelier P. L. A FFLUX Water Model: Flexible, Polarizable and With a Multipolar Description of Electrostatics. J. Comput. Chem. 2020, 41, 619–628. 10.1002/jcc.26111. 31747059 PMC7004022

[ref16] Bartók A. P.; Kermode J.; Bernstein N.; Csányi G. Machine Learning a General-Purpose Interatomic Potential for Silicon. Phys. Rev. X 2018, 8, 041048 10.1103/PhysRevX.8.041048.

[ref17] McDonagh J. L.; Shkurti A.; Bray D. J.; Anderson R. L.; Pyzer-Knapp E. O. Utilizing Machine Learning for Efficient Parameterization of Coarse Grained Molecular Force Fields. J. Chem. Inf. Model. 2019, 59, 4278–4288. 10.1021/acs.jcim.9b00646. 31549507

[ref18] Pinheiro G. A.; Mucelini J.; Soares M. D.; Prati R. C.; Da Silva J. L.; Quiles M. G. Machine Learning Prediction of Nine Molecular Properties Based on the SMILES Representation of the QM9 Quantum-Chemistry Dataset. J. Phys. Chem. A 2020, 124, 9854–9866. 10.1021/acs.jpca.0c05969. 33174750

[ref19] Burger B.; Maffettone P. M.; Gusev V. V.; Aitchison C. M.; Bai Y.; Wang X.; Li X.; Alston B. M.; Li B.; Clowes R.; et al. A Mobile Robotic Chemist. Nature 2020, 583, 237–241. 10.1038/s41586-020-2442-2. 32641813

[ref20] Stokes J. M.; Yang K.; Swanson K.; Jin W.; Cubillos-Ruiz A.; Donghia N. M.; Mac- Nair C. R.; French S.; Carfrae L. A.; Bloom-Ackermann Z.; et al. A Deep Learning Approach to Antibiotic Discovery. Cell 2020, 180, 688–702. 10.1016/j.cell.2020.01.021. 32084340 PMC8349178

[ref21] Agatonovic-Kustrin S.; Morton D. W.; Singh R. Hybrid Neural Networks as Tools for Predicting the Phase Behavior of Colloidal Systems. Colloids Surf. A Physicochem. Eng. Asp. 2012, 415, 59–67. 10.1016/j.colsurfa.2012.10.005.

[ref22] Liu D.; Bai G.; Gao C. Phase Diagrams Classification Based on Machine Learning and Phenomenological Investigation of Physical Properties in K_1–*x*_Na_*x*_NbO_3_ Thin Films. J. Appl. Phys. 2020, 127, 154101 10.1063/5.0004167.

[ref23] Aghaaminiha M.; Ghanadian S. A.; Ahmadi E.; Farnoud A. M. A Machine Learning Approach to Estimation of Phase Diagrams for Three-Component Lipid Mixtures. Biochim. Biophys. Acta Biomembr. 2020, 1862, 183350 10.1016/j.bbamem.2020.183350. 32407774 PMC7301216

[ref24] Peacock C. J.; Lamont C.; Sheen D. A.; Shen V. K.; Kreplak L.; Frampton J. P. Predicting the Mixing Behavior of Aqueous Solutions Using a Machine Learning Framework. ACS Appl. Mater. Interfaces 2021, 13, 11449–11460. 10.1021/acsami.0c21036. 33645207

[ref25] Kruglov I. A.; Yanilkin A.; Oganov A. R.; Korotaev P. Phase Diagram of Uranium From Ab Initio Calculations and Machine Learning. Phys. Rev. B 2019, 100, 174104 10.1103/PhysRevB.100.174104.

[ref26] Bell G. Non-Ionic Surfactant Phase Diagram Prediction by Recursive Partitioning. Philos. Trans. R. Soc. A 2016, 374, 20150137 10.1098/rsta.2015.0137. PMC4920284 27298439

[ref27] Laughlin R. G. The Aqueous Phase Behavior of Surfactants; Academic Press: London, 1994.

[ref28] Malcolm G. N.; Rowlinson J. S. The Thermodynamic Properties of Aqueous Solutions of Polyethylene Glycol, Polypropylene Glycol and Dioxane. Trans. Faraday Soc. 1957, 53, 921–931. 10.1039/tf9575300921.

[ref29] Saupe A. Textures, Deformations, and Structural Order of Liquid Crystals. J. Colloid Interface Sci. 1977, 58, 549–558. 10.1016/0021-9797(77)90164-3.

[ref30] Chen B.-H.; Miller C. A.; Walsh J. M.; Warren P. B.; Ruddock J. N.; Garrett P. R.; Argoul F.; Leger C. Dissolution Rates of Pure Nonionic Surfactants. Langmuir 2000, 16, 5276–5283. 10.1021/la9913497.

[ref31] Seddon J. M. Characterization Methods: Structural Studies of by X-ray Diffraction. Handbook of Liquid Crystals; Wiley, 1998; pp 635–679.

[ref32] Meredith J. C.; Karim A.; Amis E. J. High-Throughput Measurement of Polymer Blend Phase Behavior. Macromolecules 2000, 33, 5760–5762. 10.1021/ma0004662.

[ref33] Aha D. W.; Kibler D.; Albert M. K. Instance-Based Learning Algorithms. Mach. Learn. 1991, 6, 37–66. 10.1007/BF00153759.

[ref34] Lengua M. A. C.; Quiroz E. A. P. Systematic Literature Review on Support Vector Machines Applied to Classification. 2020 IEEE Engineering International Research Conference (EIRCON); IEEE, 2020; pp 1–4.

[ref35] Wang J.; Chen Q.; Chen Y. RBF Kernel Based Support Vector Machine With Universal Approximation and its Application. Advances in Neural Networks - ISNN 2004; Springer: Berlin, Heidelberg, 2004; pp 512–517.

[ref36] Breiman L.; Friedman J. H.; Olshen R. A.; Stone C. J. Classification and Regression Trees; Chapman & Hall: Boca Raton, FL, 1984.

[ref37] Hastie T.; Tibshirani R.; Friedman J. The Elements of Statistical Learning; Springer: New York, NY, 2009.

[ref38] Salzberg S. L. C4.5: Programs for machine learning by J. Ross Quinlan. Morgan Kaufmann Publishers, Inc., 1993. Mach. Learn. 1994, 16, 235–240. 10.1023/A:1022645310020.

[ref39] McCulloch W. S.; Pitts W. A Logical Calculus of the Ideas Immanent in Nervous Activity. Bull. Math. Biophys. 1943, 5, 115–133. 10.1007/BF02478259. 2185863

[ref40] Breiman L. Random Forests. Mach. Learn. 2001, 45, 5–32. 10.1023/A:1010933404324.

[ref41] Freund Y.; Schapire R. E. Machine Learning 1996, 148–156.

[ref42] John G. H.; Langley P. Estimating Continuous Distributions in Bayesian Classifiers. Proceedings of the Eleventh Conference on Uncertainty in Artificial Intelligence; Morgan Kaufmann Publishers, Inc., 1995; pp 338–345.

[ref43] Sarker I. H. Machine Learning: Algorithms, Real-World Applications and Research Directions. SN Comput. Sci. 2021, 2, 160 10.1007/s42979-021-00592-x. 33778771 PMC7983091

[ref44] Pedregosa F.; et al. Scikit-learn: Machine Learning in Python. J. Mach. Learn. Res. 2011, 12, 2825–2830.

[ref45] Mitchell D. J.; Tiddy G. J. T.; Waring L.; Bostock T.; McDonald M. P. Phase Behaviour of Polyoxyethylene Surfactants With Water. Mesophase Structures and Partial Miscibility (Cloud Points). J. Chem. Soc., Faraday Trans. 1 1983, 79, 975–1000. 10.1039/f19837900975.

[ref46] Clunie J. S.; Corkill J. M.; Goodman J. F.; Symons P. C.; Tate J. R. Thermodynamics of Non-Ionic Surface-Active Agent + Water Systems. Trans. Faraday Soc. 1967, 63, 2839–2845. 10.1039/tf9676302839.

[ref47] Nibu Y.; Inoue T. Phase Behavior of Aqueous Mixtures of Some Polyethylene Glycol Decyl Ethers Revealed by DSC and FT-IR Measurements. J. Colloid Interface Sci. 1998, 205, 305–315. 10.1006/jcis.1998.5621. 9735193

[ref48] Conroy J. P.; Hall C.; Leng C. A.; Rendall K.; Tiddy G. J. T.; Walsh J.; Lindblom G. Nonionic Surfactant Phase Behavior. The Effect of CH_3_ Capping of the Terminal OH. Accurate Measurements of Cloud Curves. Surfactants and Macromolecules: Self-Assembly at Interfaces and in Bulk. Progress in Colloid & Polymer Science 1990, 82, 253–262. 10.1007/BFb0118266.

[ref49] Kratzat K.; Finkelmann H. Branched Non-Ionic Oligo-Oxyethylene V-Amphiphiles Effect of Molecular Geometry on LC-Phase Behavior 2. Colloid Polym. Sci. 1994, 272, 400–408. 10.1007/BF00659451.

[ref50] Kratzat K.; Finkelmann H. Branched Non-Ionic Oligo-Oxyethylene Y-Amphiphiles Effect of Molecular Geometry on the Micellar Shape 1. Liq. Cryst. 1993, 13, 691–699. 10.1080/02678299308026341.

[ref51] Israelachvili J. N.; Mitchell D. J.; Ninham B. W. Theory of Self-Assembly of Hydrocarbon Amphiphiles Into Micelles and Bilayers. J. Chem. Soc., Faraday Trans. 2 1976, 72, 1525–1568. 10.1039/f29767201525.

[ref52] Nagarajan R. Molecular Packing Parameter and Surfactant Self-Assembly: The Neglected Role of the Surfactant Tail. Langmuir 2002, 18, 31–38. 10.1021/la010831y.

[ref53] Sieg J.; Flachsenberg F.; Rarey M. In Need of Bias Control: Evaluating Chemical Data for Machine Learning in Structure-Based Virtual Screening. J. Chem. Inf. Model. 2019, 59, 947–961. 10.1021/acs.jcim.8b00712. 30835112

[ref54] Good A. C.; Oprea T. I. Optimization of CAMD Techniques 3. Virtual Screening Enrichment Studies: A Help or Hindrance in Tool Selection?. J. Comput. Aided Mol. Des. 2008, 22, 169–178. 10.1007/s10822-007-9167-2. 18188508

[ref55] Huang N.; Shoichet B. K.; Irwin J. J. Benchmarking Sets for Molecular Docking. J. Med. Chem. 2006, 49, 6789–6801. 10.1021/jm0608356. 17154509 PMC3383317

[ref56] Jain A. N.; Cleves A. E. Does Your Model Weigh the Same as a Duck?. J. Comput. Aided Mol. Des. 2012, 26, 57–67. 10.1007/s10822-011-9530-1. 22187141 PMC3276372

[ref57] Van der Maaten L.; Hinton G. Visualizing Data Using t-SNE. J. Mach. Learn. Res. 2008, 9, 2579–2605.

[ref58] mlrose: Machine Learning, Randomized Optimization and SEarch package for Python. https://github.com/gkhayes/mlrose, 2019; Accessed: 14 February 2023.

[ref59] Pyzer-Knapp E.; Anderson R. L. Efficiently Populating a Phase Diagram for Multiple Substances. U.S. Patent Appl.US16/630,197, 2020.

[ref60] Katsube R.; Terayama K.; Tamura R.; Nose Y. Experimental Establishment of Phase Diagrams Guided by Uncertainty Sampling: An Application to the Deposition of Zn-Sn-P Films by Molecular Beam Epitaxy. ACS Mater. Lett. 2020, 2, 571–575. 10.1021/acsmaterialslett.0c00104.

[ref61] Dai C.; Glotzer S. C. Efficient Phase Diagram Sampling by Active Learning. J. Phys. Chem. B 2020, 124, 1275–1284. 10.1021/acs.jpcb.9b09202. 31964140

