# Supplementary material for: Can Machine Learning Predict the Phase Behavior of Surfactants?
Source: J Phys Chem B. 2023 Apr 12;127(16):3711–27. doi: 10.1021/acs.jpcb.2c08232 (PMC10150369; doi:10.1021/acs.jpcb.2c08232)
Supplement: Supplementary file 2 — jp2c08232_si_002.pdf [file jp2c08232_si_002.pdf]

Can machine learning predict the phase behavior of surfactants? – Supporting Information

Joseph C. R. Thacker,[†,‡,¶] David J. Bray,[*,†] Patrick B. Warren,[†] and Richard L. Anderson[*,†]

†*The Hartree Centre, STFC Daresbury Laboratory, Warrington, WA4 4AD, UK*

‡*Current address: Scientific Computing Department, STFC Daresbury Laboratory, Warrington, WA4 4AD, UK*

¶*Department of Chemistry, University of Liverpool, Crown Street, Liverpool, L69 7ZD, UK*

E-mail: david.bray@stfc.ac.uk; richard.anderson@stfc.ac.uk

A ML performance when using unaltered phase state labels of experimental diagrams

This section shows the results using the dataset corresponding to the original paper by Bell, with the only change being that state points originally labelled ‘M’ are replaced by ‘L1’.

A.1 Precision, Recall and F1 scores

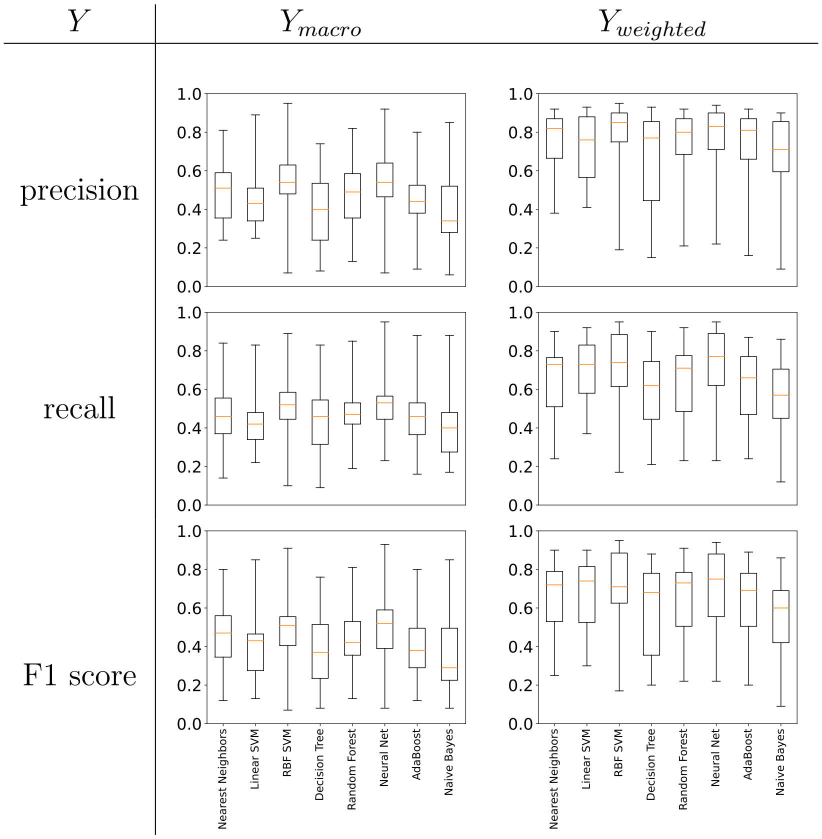

Figure S1: Plots giving the performance of different ML algorithms for the unaltered dataset across all surfactants.

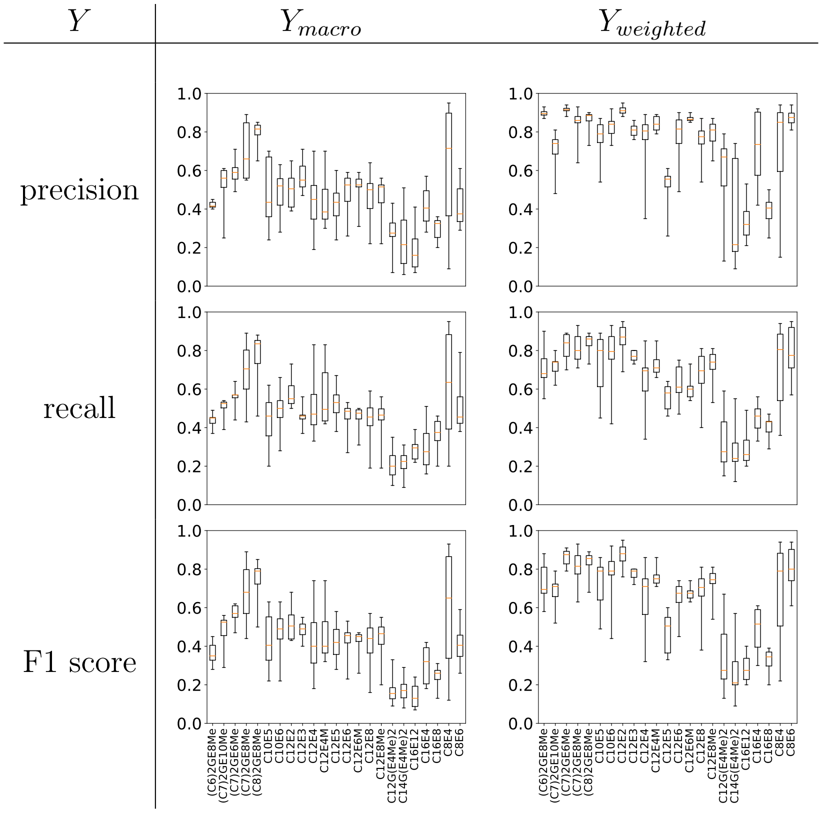

Figure S2: Plots giving the performance of different Surfactants for the unaltered dataset.

A.2 Confusion Plots: Methods

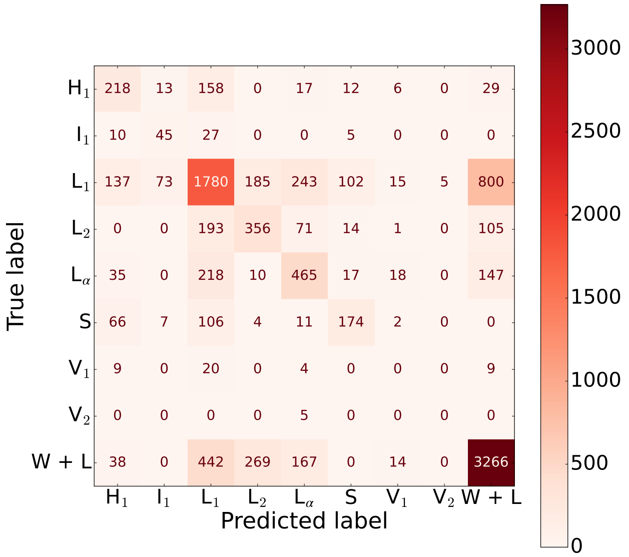

Figure S3: AdaBoost

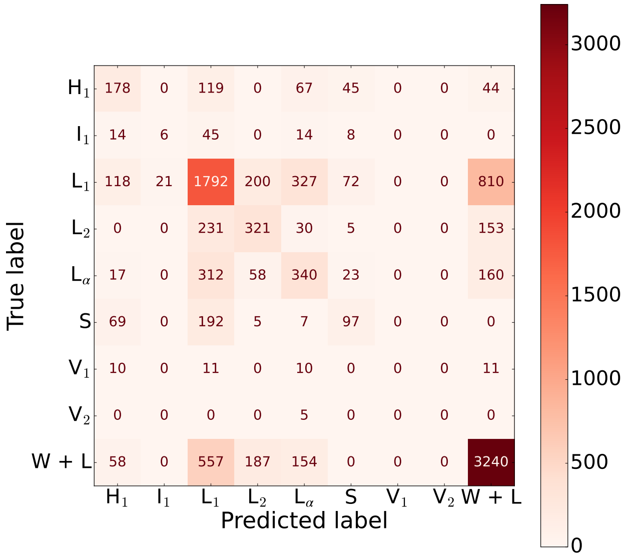

Figure S4: Decision Tree

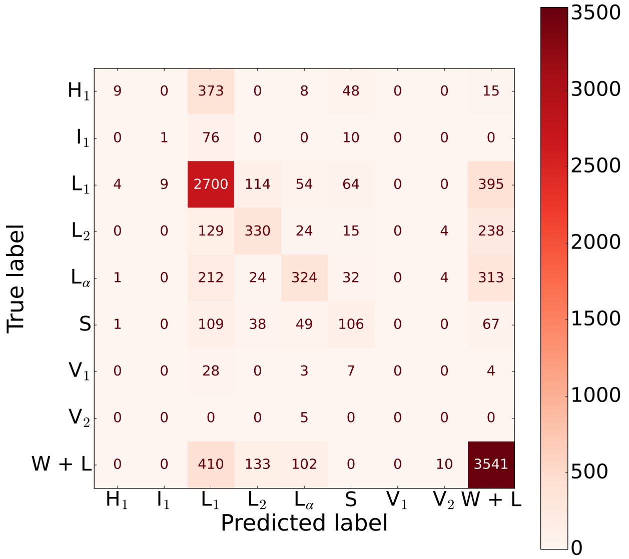

Figure S5: Linear SVM

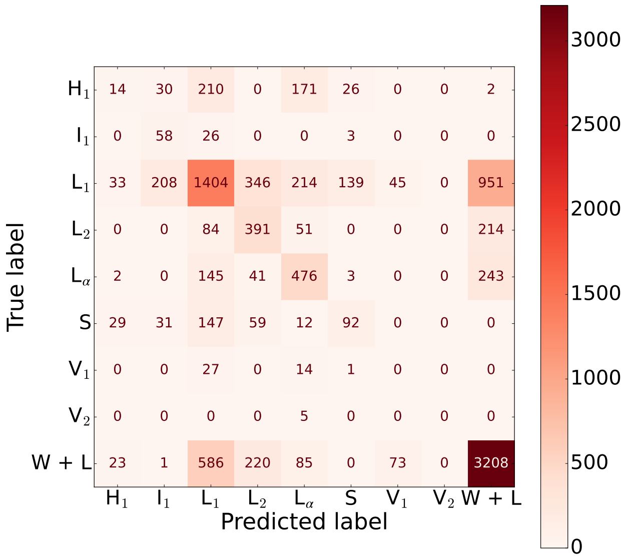

Figure S6: Naive Bayes

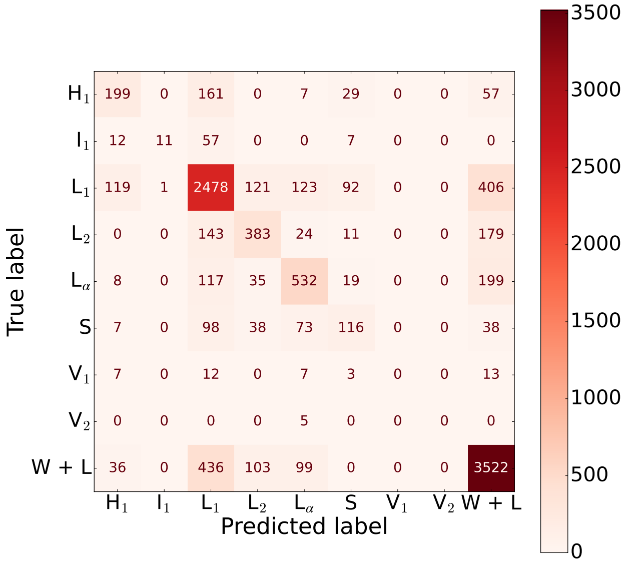

Figure S7: Neural Network

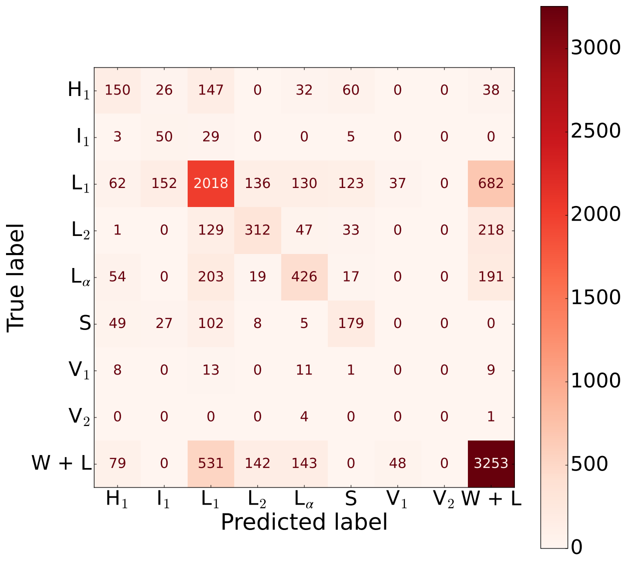

Figure S8: Random Forest

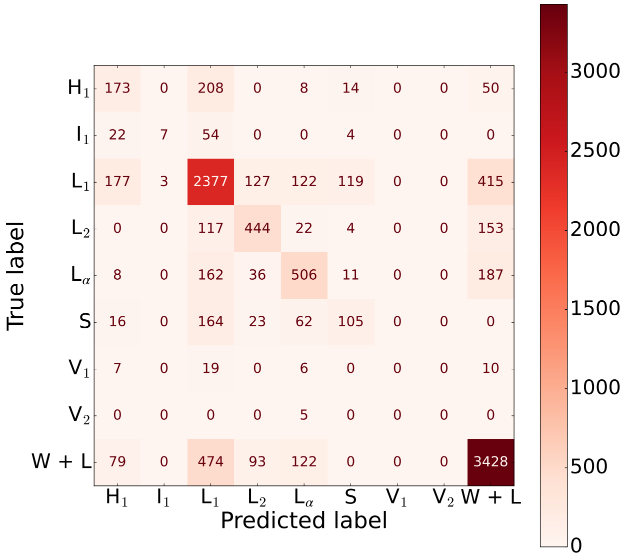

Figure S9: Radial Basis Function Support Vector Machine

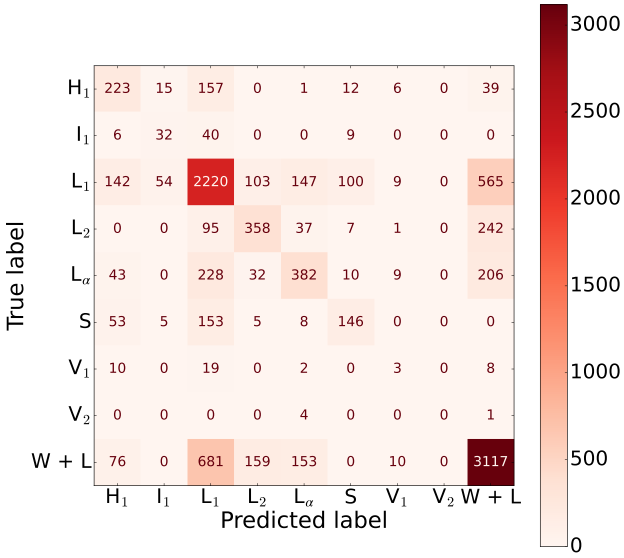

Figure S10: Nearest Neighbours

A.3 Confusion Plots: Surfactants

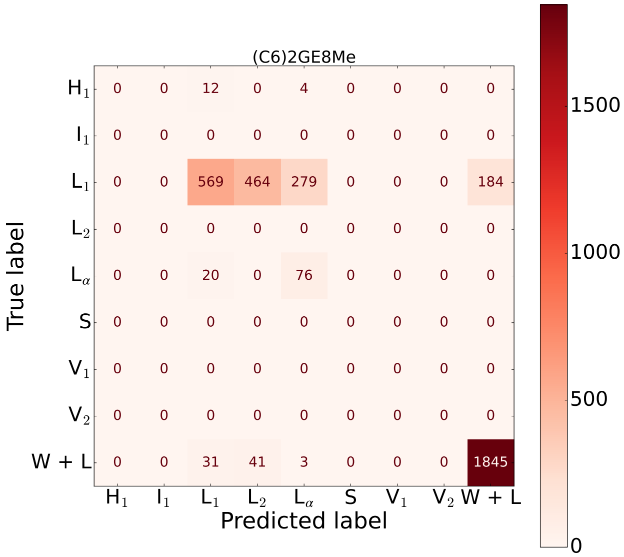

Figure S11: $(C_6)_2GE_8Me$

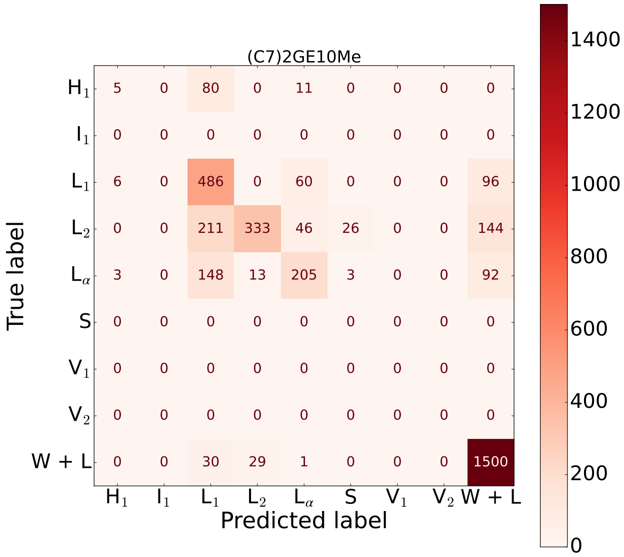

Figure S12: $(C_7)_2GE_{10}Me$

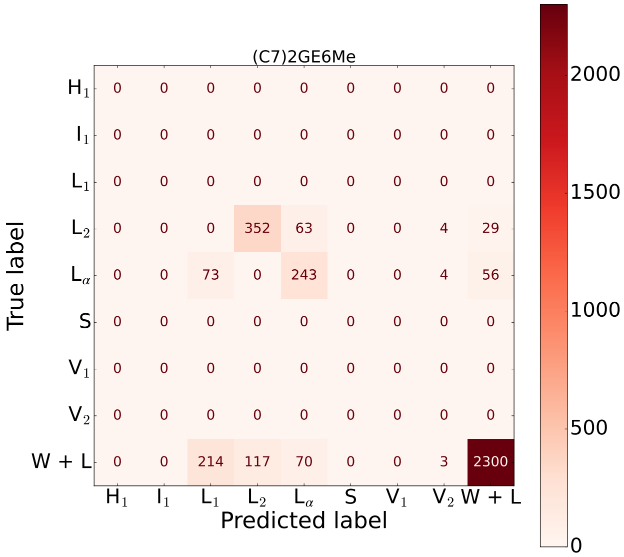

Figure S13: $(C_7)_2GE_6Me$

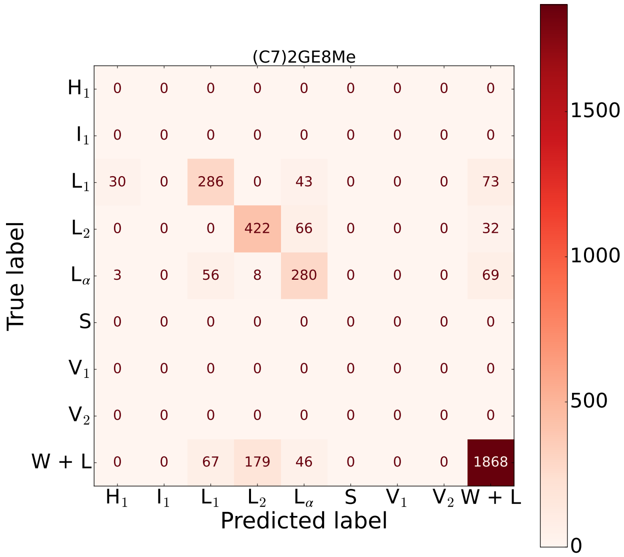

Figure S14: $(C_7)_2GE_8Me$

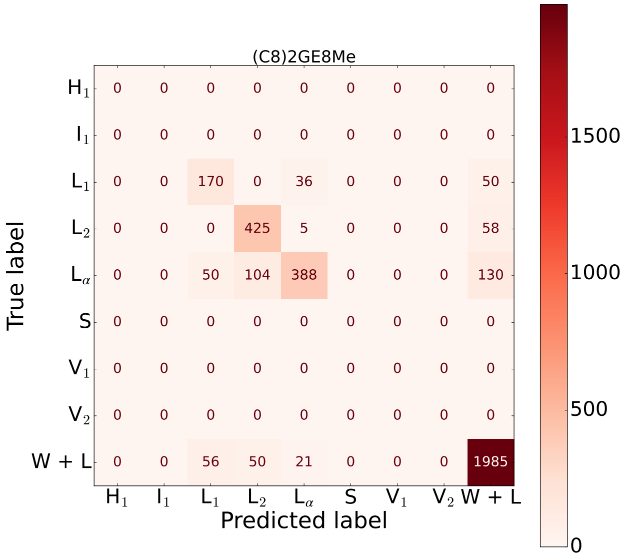

Figure S15: $(C_8)_2GE_8Me$

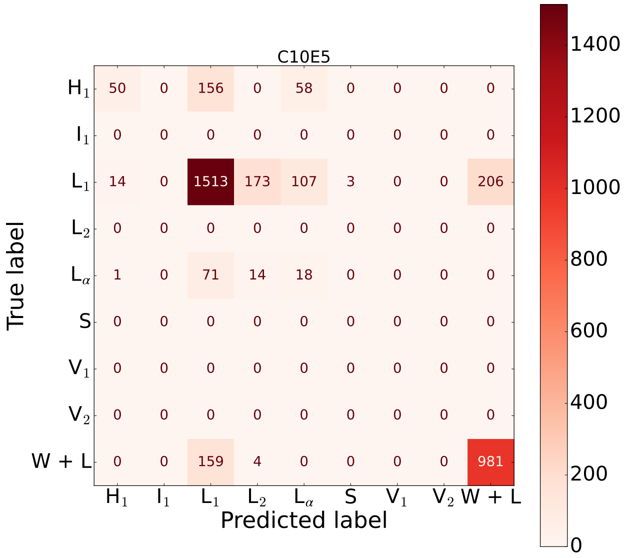

Figure S16: $C_{10}E_5$

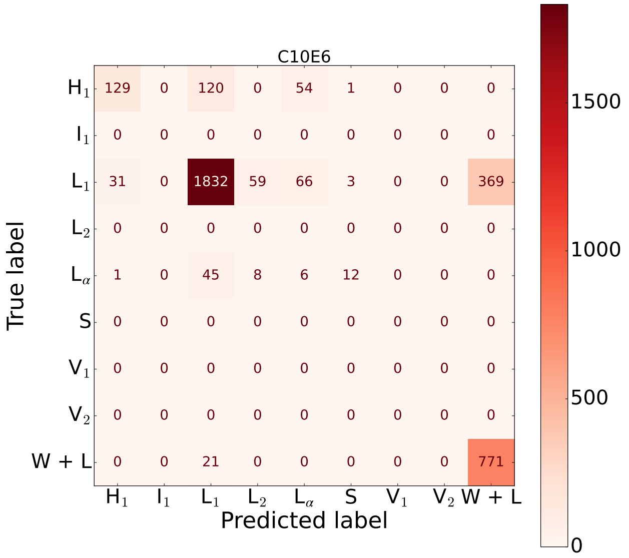

Figure S17: $C_{10}E_6$

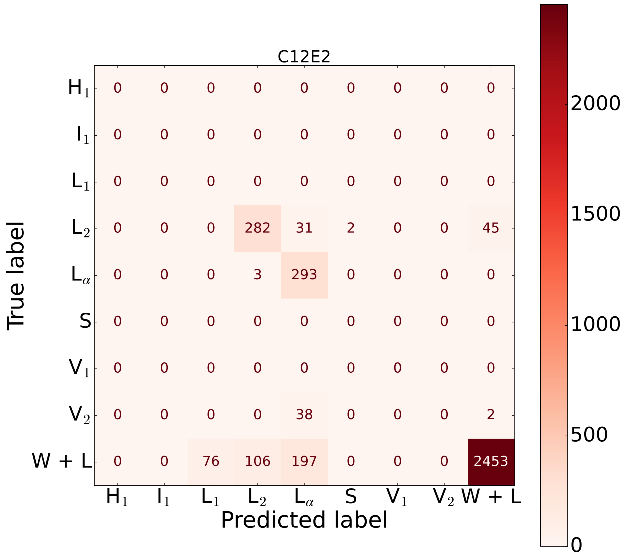

Figure S18: $C_{12}E_2$

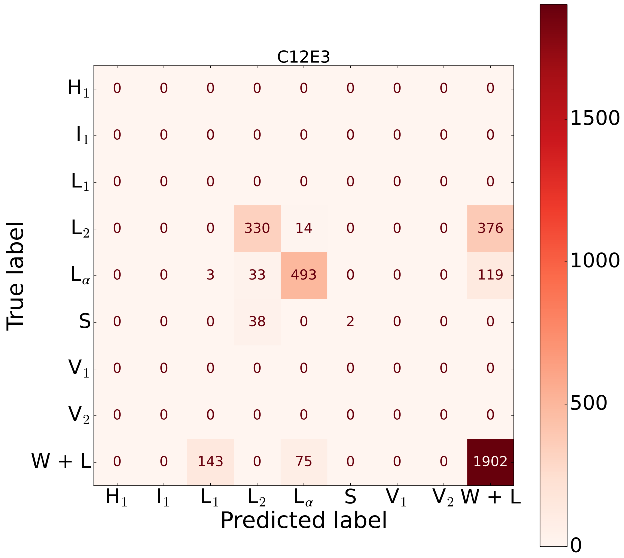

Figure S19: $C_{12}E_3$

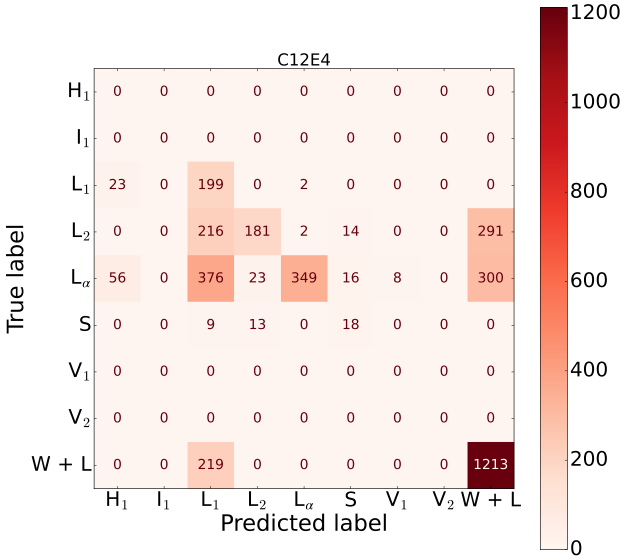

Figure S20: $C_{12}E_4$

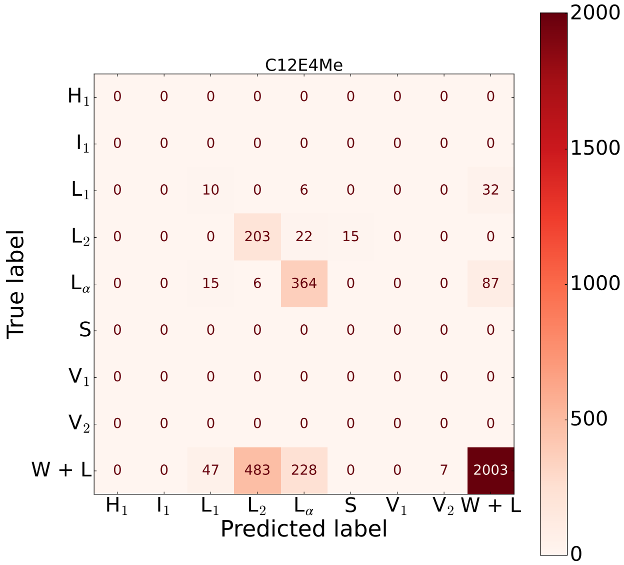

Figure S21: $C_{12}E_4Me$

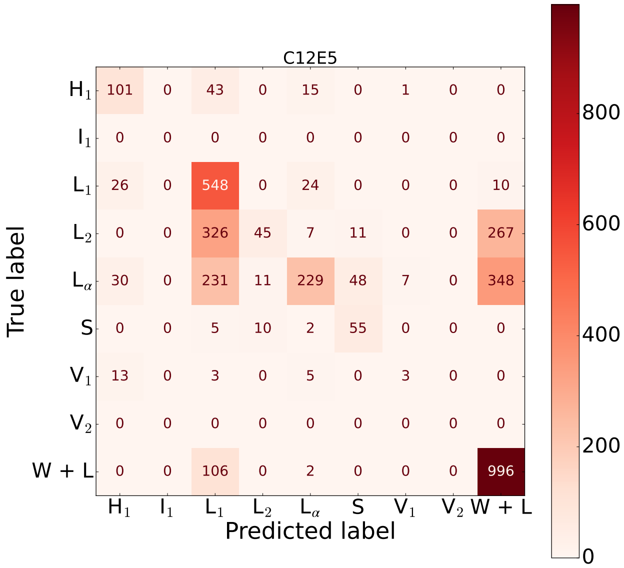

Figure S22: $C_{12}E_5$

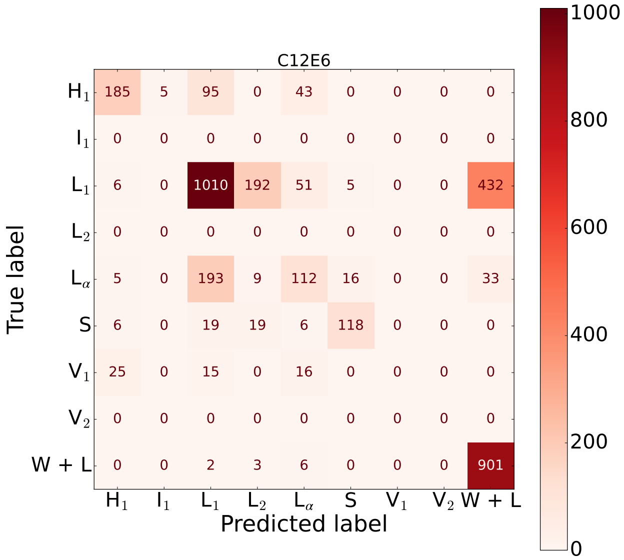

Figure S23: $C_{12}E_6$

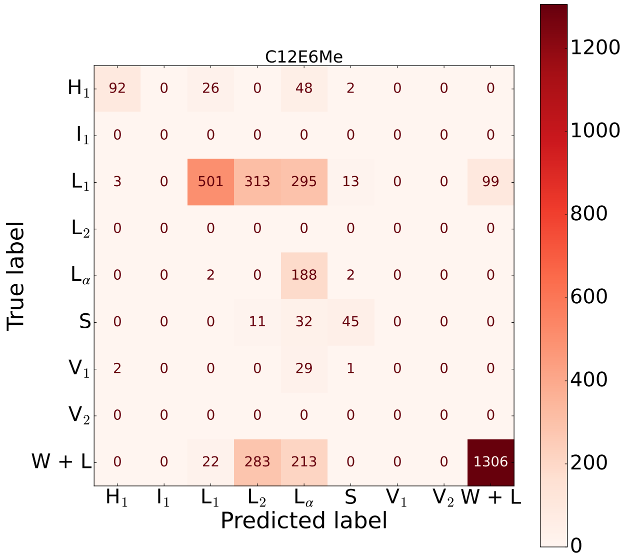

Figure S24: $C_{12}E_6Me$

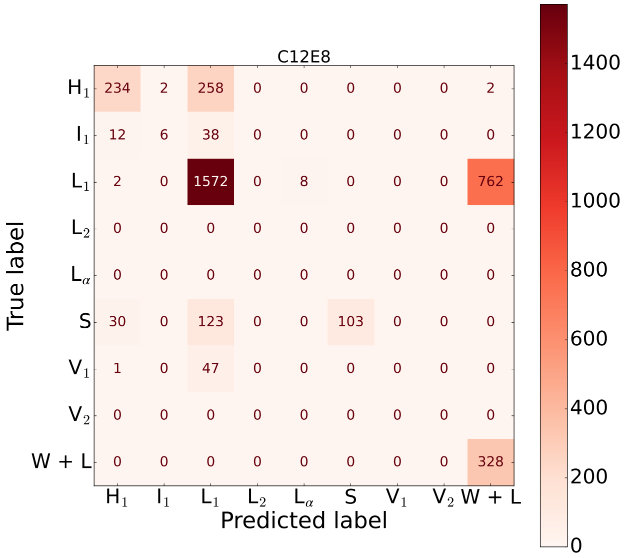

Figure S25: $C_{12}E_8$

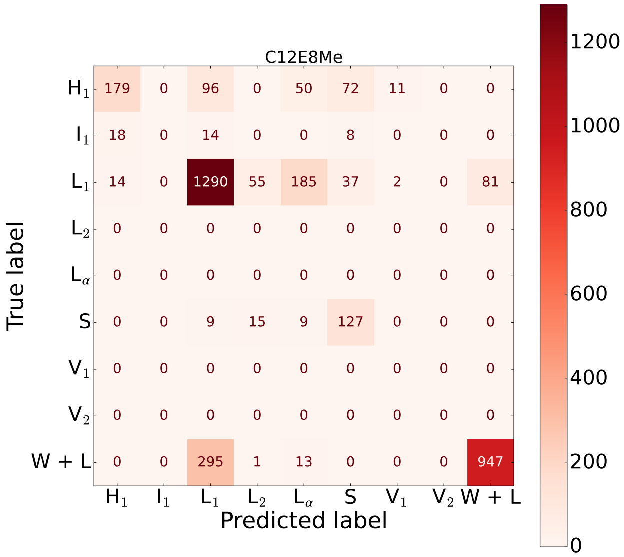

Figure S26: $C_{12}E_8Me$

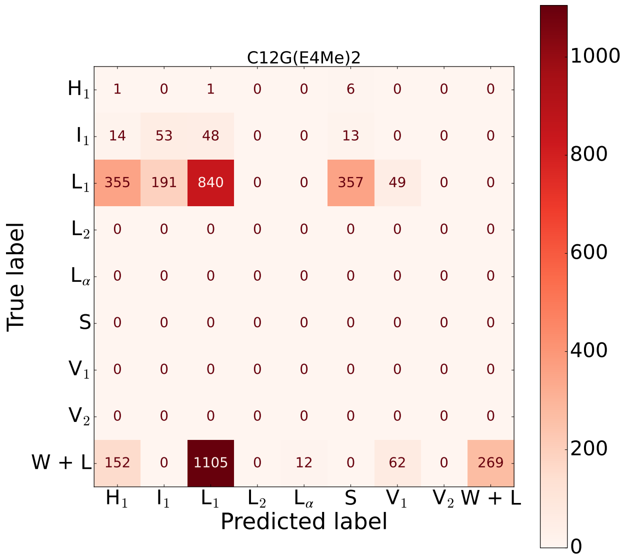

Figure S27: $C_{12}G(E_4Me)_2$

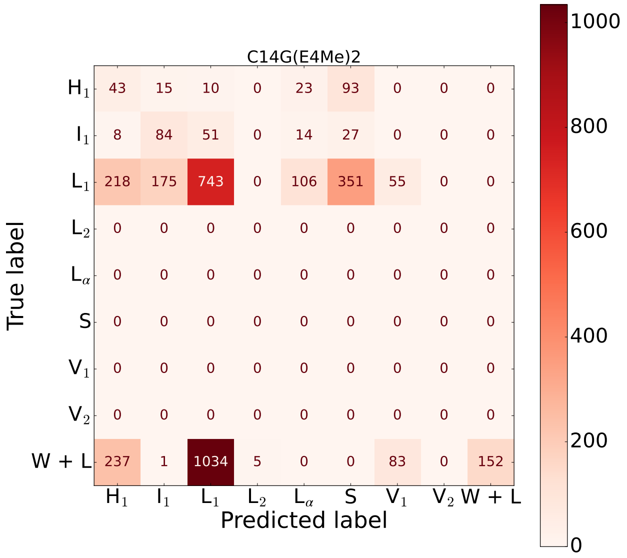

Figure S28: $C_{14}G(E_4Me)_2$

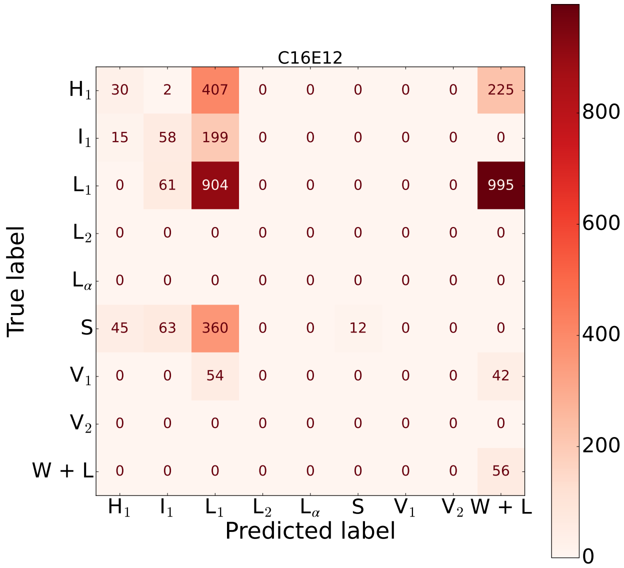

Figure S29: $C_{16}E_{12}$

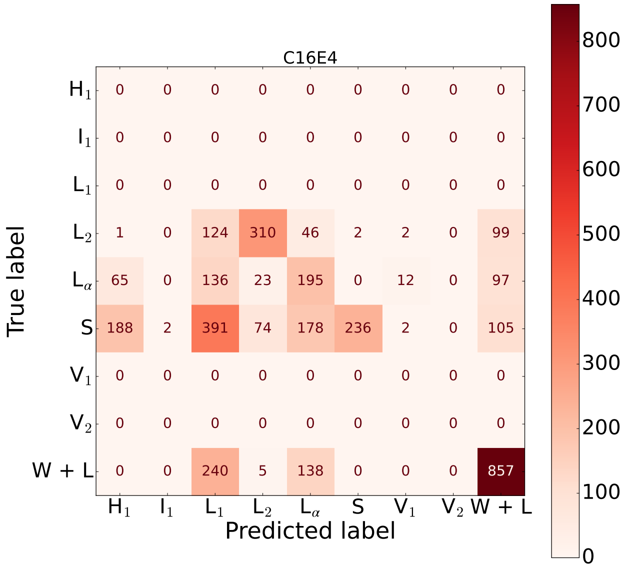

Figure S30: $C_{16}E_{4}$

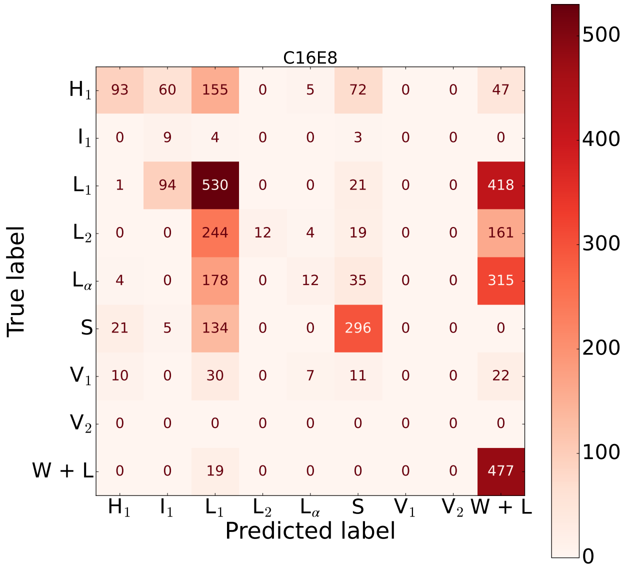

Figure S31: $C_{16}E_8$

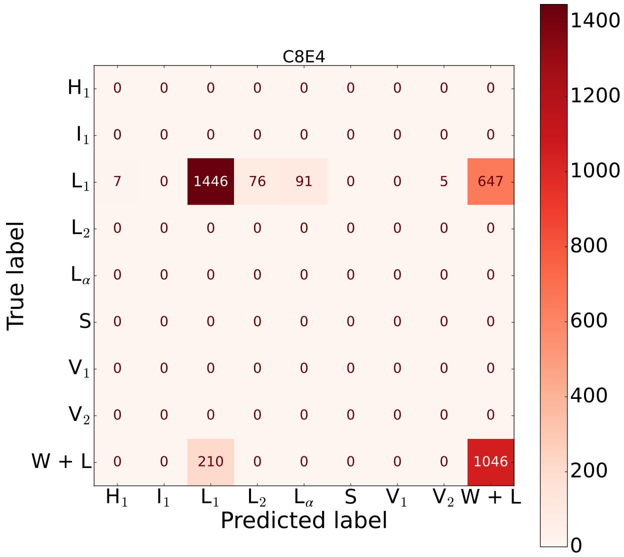

Figure S32: C_8E_4

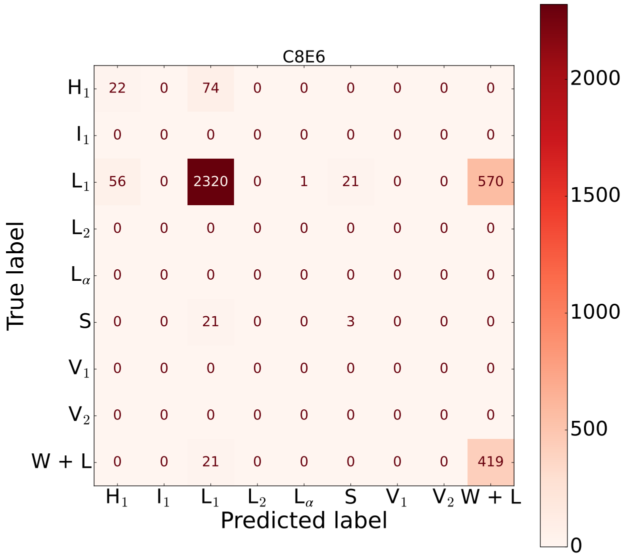

Figure S33: C_8E_6

B Sci-Kit Learn Hyper-parameters for ML Classifiers

B.1 Hyperparameter Search Grids

Nearest Neighbors : `{'leaf_size': [1, 11, 21, 31, 41, 51, 61, 71, 81, 91], 'n_neighbors': [1, 3, 5, 7, 9], 'p': (1, 2), 'weights': ('uniform', 'distance')}`

Linear SVM : `{'C': array([ 1., 112., 223., 334., 445., 556., 667., 778., 889., 1000.])}`

RBF SVM : `{'C': array([ 1., 112., 223., 334., 445., 556., 667., 778., 889., 1000.]), 'gamma': array([1.00023029e+00, 1.29181404e+01, 1.66839930e+02, 2.15476543e+03, 2.78291537e+04, 3.59418146e+05, 4.64194510e+06, 5.99514926e+07, 7.74283492e+08, 1.00000000e+10])}`

Decision Tree : `{'class_weight': (None, 'balanced'), 'criterion': ('gini', 'entropy', 'log_loss'), 'max_depth': [5, 15, 25, 35, 45, 55, 65, 75, 85, 95], 'min_samples_leaf': [3, 6, 9, 12, 15, 18, 21], 'min_samples_split': [2, 4, 6, 8, 10, 12, 14, 16, 18, 20]}`

Random Forest : `{'class_weight': (None, 'balanced'), 'criterion': ('gini', 'entropy', 'log_loss'), 'max_depth': [5, 15, 25, 35, 45, 55, 65, 75, 85, 95], 'min_samples_leaf': [3, 6, 9, 12, 15, 18, 21], 'min_samples_split': [2, 4, 6, 8, 10, 12, 14, 16, 18, 20], 'n_estimators': [2, 4, 6, 8, 10, 12, 14, 16, 18]}`

Neural Net : `{'activation': ('relu', 'tanh'), 'hidden_layer_sizes': [(25, 50, 25), (50, 50, 50), (75, 75), (100,), (25, 25)], 'alpha': [0.0001, 0.01, 0.1, 0.5, 1.0], 'solver': ['sdg', 'adam'], 'learning_rate': ['adaptive', 'constant']}`

AdaBoost : `{'n_estimators': [10, 30, 50, 70, 100]}`

Naive Bayes : `{'var_smoothing': array([1.e+00, 1.e-01, 1.e-02, 1.e-03, 1.e-04, 1.e-05, 1.e-06, 1.e-07, 1.e-08, 1.e-09])}`

B.2 Optimal Hyperparameters Determined by Grid Search (Gap Filling Challenge)

AdaBoost : {"n_estimators": 100}

Decision Tree : {"class_weight": null, "criterion": "entropy", "max_depth": 65, "min_samples_leaf": 3, "min_samples_split": 6}

Linear SVM : {"C": 1000.0}

Naive Bayes : {"var_smoothing": 0.001}

Nearest Neighbors : {"leaf_size": 51, "n_neighbors": 9, "p": 1, "weights": "distance"}

Neural Net : {"activation": "tanh", "alpha": 0.0001, "hidden_layer_sizes": [50, 50, 50], "learning_rate": "constant", "solver": "adam"}

Random Forest : {"class_weight": null, "criterion": "log_loss", "max_depth": 25, "min_samples_leaf": 3, "min_samples_split": 2, "n_estimators": 18}

RBF SVM : {"C": 1000.0, "gamma": 12.918140390030565}

B.3 Optimal Hyperparameters Determined by Grid Search (*de novo* Challenge)

AdaBoost C10E6 : {"n_estimators": 100}

AdaBoost C12E2 : {"n_estimators": 70}

AdaBoost C12E3 : {"n_estimators": 100}

AdaBoost C12E4Me : {"n_estimators": 100}

AdaBoost C12E4 : {"n_estimators": 100}

AdaBoost C12E5 : {"n_estimators": 50}

AdaBoost C12E6Me : {"n_estimators": 100}

AdaBoost C12E6 : {"n_estimators": 30}

AdaBoost C12E8Me : {"n_estimators": 100}

AdaBoost C12E8 : {"n_estimators": 70}

AdaBoost C12G(E4Me)2 : {"n_estimators": 70}

AdaBoost C14G(E4Me)2 : {"n_estimators": 100}

AdaBoost C16E12 : {"n_estimators": 50}

AdaBoost C16E4 : {"n_estimators": 30}

AdaBoost C16E8 : {"n_estimators": 100}

AdaBoost (C6)2GE8Me : {"n_estimators": 70}

AdaBoost (C7)2GE10Me : {"n_estimators": 70}

AdaBoost (C7)2GE6Me : {"n_estimators": 70}

AdaBoost (C7)2GE8Me : {"n_estimators": 100}

AdaBoost (C8)2GE8Me : {"n_estimators": 100}

AdaBoost C8E4 : {"n_estimators": 100}

AdaBoost C8E6 : {"n_estimators": 100}

Decision Tree C10E5 : {"class_weight": null, "criterion": "log_loss", "max_depth": 45, "min_samples_leaf": 21, "min_samples_split": 12}

Decision Tree C10E6 : {"class_weight": null, "criterion": "entropy", "max_depth": 35, "min_samples_leaf": 6, "min_samples_split": 12}

Decision Tree C12E2 : {"class_weight": null, "criterion": "gini", "max_depth": 95, "min_samples_leaf": 3, "min_samples_split": 4}

Decision Tree C12E3 : {"class_weight": null, "criterion": "gini", "max_depth": 5, "min_samples_leaf": 18, "min_samples_split": 20}

Decision Tree C12E4Me : {"class_weight": null, "criterion": "entropy", "max_depth": 5, "min_samples_leaf": 3, "min_samples_split": 4}

Decision Tree C12E4 : {"class_weight": "balanced", "criterion": "log_loss", "max_depth": 15, "min_samples_leaf": 3, "min_samples_split": 6}

Decision Tree C12E5 : {"class_weight": null, "criterion": "gini", "max_depth": 5, "min_samples_leaf": 3, "min_samples_split": 4}

Decision Tree C12E6Me : {"class_weight": null, "criterion": "gini", "max_depth": 75, "min_samples_leaf": 9, "min_samples_split": 4}

Decision Tree C12E6 : {"class_weight": null, "criterion": "entropy", "max_depth": 5, "min_samples_leaf": 3, "min_samples_split": 2}

Decision Tree C12E8Me : {"class_weight": null, "criterion": "entropy", "max_depth": 25, "min_samples_leaf": 12, "min_samples_split": 8}

Decision Tree C12E8 : {"class_weight": null, "criterion": "gini", "max_depth": 15, "min_samples_leaf": 12, "min_samples_split": 16}

Decision Tree C12G(E4Me)2 : {"class_weight": null, "criterion": "gini", "max_depth": 85, "min_samples_leaf": 6, "min_samples_split": 12}

Decision Tree C14G(E4Me)2 : {"class_weight": null, "criterion": "gini", "max_depth": 5, "min_samples_leaf": 9, "min_samples_split": 2}

Decision Tree C16E12 : {"class_weight": null, "criterion": "gini", "max_depth": 5, "min_samples_leaf": 3, "min_samples_split": 4}

Decision Tree C16E4 : {"class_weight": null, "criterion": "gini", "max_depth": 5, "min_samples_leaf": 3, "min_samples_split": 14}

Decision Tree C16E8 : {"class_weight": null, "criterion": "entropy", "max_depth": 5, "min_samples_leaf": 3, "min_samples_split": 18}

Decision Tree (C6)2GE8Me : {"class_weight": null, "criterion": "entropy", "max_depth": 35, "min_samples_leaf": 6, "min_samples_split": 2}

Decision Tree (C7)2GE10Me : {"class_weight": null, "criterion": "entropy", "max_depth": 55, "min_samples_leaf": 6, "min_samples_split": 8}

Decision Tree (C7)2GE6Me : {"class_weight": null, "criterion": "gini", "max_depth": 15, "min_samples_leaf": 3, "min_samples_split": 8}

Decision Tree (C7)2GE8Me : {"class_weight": null, "criterion": "log_loss", "max_depth": 65, "min_samples_leaf": 6, "min_samples_split": 14}

Decision Tree (C8)2GE8Me : {"class_weight": null, "criterion": "log_loss", "max_depth": 65, "min_samples_leaf": 21, "min_samples_split": 12}

Decision Tree C8E4 : {"class_weight": null, "criterion": "gini", "max_depth": 5, "min_samples_leaf": 3, "min_samples_split": 8}

Decision Tree C8E6 : {"class_weight": null, "criterion": "gini", "max_depth": 5, "min_samples_leaf": 3, "min_samples_split": 2}

Linear SVM C10E5 : {"C": 1000.0}

Linear SVM C10E6 : {"C": 1000.0}

Linear SVM C12E2 : {"C": 1000.0}

Linear SVM C12E3 : {"C": 1000.0}

Linear SVM C12E4Me : {"C": 1000.0}

Linear SVM C12E4 : {"C": 1000.0}

Linear SVM C12E5 : {"C": 1000.0}

Linear SVM C12E6Me : {"C": 1000.0}

Linear SVM C12E6 : {"C": 1000.0}

Linear SVM C12E8Me : {"C": 889.0}

Linear SVM C12E8 : {"C": 1000.0}

Linear SVM C12G(E4Me)2 : {"C": 778.0}

Linear SVM C14G(E4Me)2 : {"C": 1000.0}

Linear SVM C16E12 : {"C": 1000.0}

Linear SVM C16E4 : {"C": 1000.0}

Linear SVM C16E8 : {"C": 1000.0}

Linear SVM (C6)2GE8Me : {"C": 1.0}

Linear SVM (C7)2GE10Me : {"C": 1.0}

Linear SVM (C7)2GE6Me : {"C": 889.0}

Linear SVM (C7)2GE8Me : {"C": 889.0}

Linear SVM (C8)2GE8Me : {"C": 889.0}

Linear SVM C8E4 : {"C": 889.0}

Linear SVM C8E6 : {"C": 1000.0}

Naive Bayes C10E5 : {"var_smoothing": 0.01}

Naive Bayes C10E6 : {"var_smoothing": 0.0001}

Naive Bayes C12E2 : {"var_smoothing": 0.01}

Naive Bayes C12E3 : {"var_smoothing": 0.001}

Naive Bayes C12E4Me : {"var_smoothing": 0.001}

Naive Bayes C12E4 : {"var_smoothing": 0.001}

Naive Bayes C12E5 : {"var_smoothing": 0.001}

Naive Bayes C12E6Me : {"var_smoothing": 0.001}

Naive Bayes C12E6 : {"var_smoothing": 0.001}

Naive Bayes C12E8Me : {"var_smoothing": 0.0001}

Naive Bayes C12E8 : {"var_smoothing": 0.001}

Naive Bayes C12G(E4Me)2 : {"var_smoothing": 0.001}

Naive Bayes C14G(E4Me)2 : {"var_smoothing": 0.001}

Naive Bayes C16E12 : {"var_smoothing": 0.001}

Naive Bayes C16E4 : {"var_smoothing": 1e-05}

Naive Bayes C16E8 : {"var_smoothing": 0.0001}

Naive Bayes (C6)2GE8Me : {"var_smoothing": 0.001}

Naive Bayes (C7)2GE10Me : {"var_smoothing": 0.001}

Naive Bayes (C7)2GE6Me : {"var_smoothing": 0.0001}

Naive Bayes (C7)2GE8Me : {"var_smoothing": 1.0}

Naive Bayes (C8)2GE8Me : {"var_smoothing": 1.0}

Naive Bayes C8E4 : {"var_smoothing": 0.0001}

Naive Bayes C8E6 : {"var_smoothing": 0.0001}

Nearest Neighbors C10E5 : {"leaf_size": 21, "n_neighbors": 3, "p": 1, "weights": "uniform"}

Nearest Neighbors C10E6 : {"leaf_size": 21, "n_neighbors": 3, "p": 1, "weights": "uniform"}

Nearest Neighbors C12E2 : {"leaf_size": 21, "n_neighbors": 3, "p": 1, "weights": "uniform"}

Nearest Neighbors C12E3 : {"leaf_size": 11, "n_neighbors": 3, "p": 1, "weights": "uniform"}

Nearest Neighbors C12E4Me : {"leaf_size": 71, "n_neighbors": 3, "p": 1,

"weights": "uniform"}

Nearest Neighbors C12E4 : {"leaf_size": 11, "n_neighbors": 3, "p": 1, "weights": "uniform"}

Nearest Neighbors C12E5 : {"leaf_size": 71, "n_neighbors": 3, "p": 1, "weights": "uniform"}

Nearest Neighbors C12E6Me : {"leaf_size": 71, "n_neighbors": 3, "p": 1, "weights": "uniform"}

Nearest Neighbors C12E6 : {"leaf_size": 71, "n_neighbors": 3, "p": 1, "weights": "uniform"}

Nearest Neighbors C12E8Me : {"leaf_size": 21, "n_neighbors": 3, "p": 1, "weights": "uniform"}

Nearest Neighbors C12E8 : {"leaf_size": 11, "n_neighbors": 3, "p": 1, "weights": "uniform"}

Nearest Neighbors C12G(E4Me)2 : {"leaf_size": 11, "n_neighbors": 7, "p": 1, "weights": "uniform"}

Nearest Neighbors C14G(E4Me)2 : {"leaf_size": 11, "n_neighbors": 3, "p": 1, "weights": "uniform"}

Nearest Neighbors C16E12 : {"leaf_size": 21, "n_neighbors": 9, "p": 1, "weights": "uniform"}

Nearest Neighbors C16E4 : {"leaf_size": 21, "n_neighbors": 9, "p": 1, "weights": "uniform"}

Nearest Neighbors C16E8 : {"leaf_size": 41, "n_neighbors": 9, "p": 1, "weights": "uniform"}

Nearest Neighbors (C6)2GE8Me : {"leaf_size": 21, "n_neighbors": 3, "p": 1, "weights": "uniform"}

Nearest Neighbors (C7)2GE10Me : {"leaf_size": 71, "n_neighbors": 3, "p": 1, "weights": "uniform"}

Nearest Neighbors (C7)2GE6Me : {"leaf_size": 71, "n_neighbors": 3, "p": 1, "weights": "uniform"}

Nearest Neighbors (C7)2GE8Me : {"leaf_size": 21, "n_neighbors": 3, "p": 1, "weights": "uniform"}

Nearest Neighbors (C8)2GE8Me : {"leaf_size": 21, "n_neighbors": 3, "p": 1, "weights": "uniform"}

Nearest Neighbors C8E4 : {"leaf_size": 1, "n_neighbors": 9, "p": 1, "weights": "uniform"}

Nearest Neighbors C8E6 : {"leaf_size": 41, "n_neighbors": 9, "p": 1, "weights": "uniform"}

Neural Net C10E5 : {"activation": "tanh", "alpha": 0.5, "hidden_layer_sizes": [50, 50, 50], "learning_rate": "constant", "solver": "adam"}

Neural Net C10E6 : {"activation": "relu", "alpha": 0.5, "hidden_layer_sizes": [50, 50, 50], "learning_rate": "adaptive", "solver": "adam"}

Neural Net C12E2 : {"activation": "tanh", "alpha": 1.0, "hidden_layer_sizes": [50, 50, 50], "learning_rate": "constant", "solver": "adam"}

Neural Net C12E3 : {"activation": "tanh", "alpha": 1.0, "hidden_layer_sizes": [50, 50, 50], "learning_rate": "constant", "solver": "adam"}

Neural Net C12E4Me : {"activation": "tanh", "alpha": 1.0, "hidden_layer_sizes": [50, 50, 50], "learning_rate": "constant", "solver": "adam"}

Neural Net C12E4 : {"activation": "tanh", "alpha": 0.5, "hidden_layer_sizes": [25, 50, 25], "learning_rate": "constant", "solver": "adam"}

Neural Net C12E5 : {"activation": "relu", "alpha": 1.0, "hidden_layer_sizes": [50, 50, 50], "learning_rate": "constant", "solver": "adam"}

Neural Net C12E6Me : {"activation": "tanh", "alpha": 1.0, "hidden_layer_sizes": [25, 50, 25], "learning_rate": "constant", "solver": "adam"}

Neural Net C12E6 : {"activation": "tanh", "alpha": 0.5, "hidden_layer_sizes": [75, 75], "learning_rate": "adaptive", "solver": "adam"}

Neural Net C12E8Me : {"activation": "relu", "alpha": 0.5, "hidden_layer_sizes": [75, 75], "learning_rate": "constant", "solver": "adam"}

Neural Net C12E8 : {"activation": "relu", "alpha": 0.5, "hidden_layer_sizes": [50, 50, 50], "learning_rate": "adaptive", "solver": "adam"}

Neural Net C12G(E4Me)2 : {"activation": "relu", "alpha": 0.5, "hidden_layer_sizes": [75, 75], "learning_rate": "constant", "solver": "adam"}

Neural Net C14G(E4Me)2 : {"activation": "relu", "alpha": 0.1, "hidden_layer_sizes": [100], "learning_rate": "adaptive", "solver": "adam"}

Neural Net C16E12 : {"activation": "relu", "alpha": 1.0, "hidden_layer_sizes": [25, 50, 25], "learning_rate": "adaptive", "solver": "adam"}

Neural Net C16E4 : {"activation": "relu", "alpha": 0.1, "hidden_layer_sizes": [25, 25], "learning_rate": "constant", "solver": "adam"}

Neural Net C16E8 : {"activation": "tanh", "alpha": 0.5, "hidden_layer_sizes": [75, 75], "learning_rate": "constant", "solver": "adam"}

Neural Net (C6)2GE8Me : {"activation": "relu", "alpha": 0.5, "hidden_layer_sizes": [75, 75], "learning_rate": "constant", "solver": "adam"}

Neural Net (C7)2GE10Me : {"activation": "relu", "alpha": 0.5, "hidden_layer_sizes": [25, 25], "learning_rate": "adaptive", "solver": "adam"}

Neural Net (C7)2GE6Me : {"activation": "relu", "alpha": 0.1, "hidden_layer_sizes": [100], "learning_rate": "adaptive", "solver": "adam"}

Neural Net (C7)2GE8Me : {"activation": "tanh", "alpha": 1.0, "hidden_layer_sizes": [25, 50, 25], "learning_rate": "constant", "solver": "adam"}

Neural Net (C8)2GE8Me : {"activation": "relu", "alpha": 0.5, "hidden_layer_sizes": [75, 75], "learning_rate": "adaptive", "solver": "adam"}

Neural Net C8E4 : {"activation": "tanh", "alpha": 0.1, "hidden_layer_sizes": [100], "learning_rate": "constant", "solver": "adam"}

Neural Net C8E6 : {"activation": "tanh", "alpha": 0.5, "hidden_layer_sizes": [25, 25], "learning_rate": "adaptive", "solver": "adam"}

Random Forest C10E5 : {"class_weight": null, "criterion": "entropy", "max_depth": 15, "min_samples_leaf": 12, "min_samples_split": 2, "n_estimators": 10}

Random Forest C10E6 : {"class_weight": null, "criterion": "log_loss", "max_depth": 25, "min_samples_leaf": 18, "min_samples_split": 20, "n_estimators": 12}

Random Forest C12E2 : {"class_weight": null, "criterion": "log_loss", "max_depth": 55, "min_samples_leaf": 6, "min_samples_split": 6, "n_estimators": 4}

Random Forest C12E3 : {"class_weight": null, "criterion": "log_loss", "max_depth": 25, "min_samples_leaf": 18, "min_samples_split": 18, "n_estimators": 6}

Random Forest C12E4Me : {"class_weight": null, "criterion": "log_loss", "max_depth": 25, "min_samples_leaf": 3, "min_samples_split": 18, "n_estimators": 12}

Random Forest C12E4 : {"class_weight": null, "criterion": "log_loss", "max_depth": 15, "min_samples_leaf": 3, "min_samples_split": 2, "n_estimators": 10}

Random Forest C12E5 : {"class_weight": null, "criterion": "gini", "max_depth": 65, "min_samples_leaf": 21, "min_samples_split": 14, "n_estimators": 8}

Random Forest C12E6Me : {"class_weight": null, "criterion": "gini", "max_depth": 35, "min_samples_leaf": 9, "min_samples_split": 6, "n_estimators": 6}

Random Forest C12E6 : {"class_weight": "balanced", "criterion": "log_loss", "max_depth": 5, "min_samples_leaf": 3, "min_samples_split": 6, "n_estimators": 8}

Random Forest C12E8Me : {"class_weight": null, "criterion": "log_loss", "max_depth": 25, "min_samples_leaf": 15, "min_samples_split": 16, "n_estimators": 16}

Random Forest C12E8 : {"class_weight": null, "criterion": "log_loss", "max_depth": 65, "min_samples_leaf": 12, "min_samples_split": 4, "n_estimators": 12}

Random Forest C12G(E4Me)2 : {"class_weight": "balanced", "criterion": "log_loss", "max_depth": 65, "min_samples_leaf": 21, "min_samples_split": 14, "n_estimators": 12}

Random Forest C14G(E4Me)2 : {"class_weight": "balanced", "criterion": "gini", "max_depth": 95, "min_samples_leaf": 21, "min_samples_split": 14, "n_estimators": 12}

Random Forest C16E12 : {"class_weight": "balanced", "criterion": "gini", "max_depth": 25, "min_samples_leaf": 12, "min_samples_split": 12, "n_estimators": 12}

Random Forest C16E4 : {"class_weight": null, "criterion": "log_loss", "max_depth": 45, "min_samples_leaf": 15, "min_samples_split": 10, "n_estimators": 12}

Random Forest C16E8 : {"class_weight": "balanced", "criterion": "gini", "max_depth": 25, "min_samples_leaf": 18, "min_samples_split": 10, "n_estimators": 16}

Random Forest (C6)2GE8Me : {"class_weight": null, "criterion": "log_loss", "max_depth": 55, "min_samples_leaf": 6, "min_samples_split": 8, "n_estimators": 8}

Random Forest (C7)2GE10Me : {"class_weight": null, "criterion": "log_loss", "max_depth": 35, "min_samples_leaf": 15, "min_samples_split": 20, "n_estimators": 10}

Random Forest (C7)2GE6Me : {"class_weight": null, "criterion": "entropy", "max_depth": 85, "min_samples_leaf": 12, "min_samples_split": 20, "n_estimators": 16}

Random Forest (C7)2GE8Me : {"class_weight": null, "criterion": "gini",

"max_depth": 65, "min_samples_leaf": 6, "min_samples_split": 4, "n_estimators": 18}

Random Forest (C8)2GE8Me : {"class_weight": null, "criterion": "entropy", "max_depth": 25, "min_samples_leaf": 12, "min_samples_split": 12, "n_estimators": 14}

Random Forest C8E4 : {"class_weight": null, "criterion": "entropy", "max_depth": 85, "min_samples_leaf": 21, "min_samples_split": 10, "n_estimators": 12}

Random Forest C8E6 : {"class_weight": null, "criterion": "gini", "max_depth": 55, "min_samples_leaf": 21, "min_samples_split": 18, "n_estimators": 18}

RBF SVM C10E5 : {"C": 1.0, "gamma": 1.0002302850208247}

RBF SVM C10E6 : {"C": 1.0, "gamma": 1.0002302850208247}

RBF SVM C12E2 : {"C": 1.0, "gamma": 1.0002302850208247}

RBF SVM C12E3 : {"C": 1.0, "gamma": 1.0002302850208247}

RBF SVM C12E4Me : {"C": 1.0, "gamma": 1.0002302850208247}

RBF SVM C12E4 : {"C": 1.0, "gamma": 1.0002302850208247}

RBF SVM C12E5 : {"C": 1.0, "gamma": 1.0002302850208247}

RBF SVM C12E6Me : {"C": 1.0, "gamma": 1.0002302850208247}

RBF SVM C12E6 : {"C": 1.0, "gamma": 1.0002302850208247}

RBF SVM C12E8Me : {"C": 1.0, "gamma": 1.0002302850208247}

RBF SVM C12E8 : {"C": 1.0, "gamma": 1.0002302850208247}

RBF SVM C12G(E4Me)2 : {"C": 1.0, "gamma": 1.0002302850208247}

RBF SVM C14G(E4Me)2 : {"C": 1.0, "gamma": 1.0002302850208247}

RBF SVM C16E12 : {"C": 1.0, "gamma": 1.0002302850208247}

RBF SVM C16E4 : {"C": 1.0, "gamma": 1.0002302850208247}

RBF SVM C16E8 : {"C": 1.0, "gamma": 1.0002302850208247}

RBF SVM (C6)2GE8Me : {"C": 1.0, "gamma": 1.0002302850208247}

RBF SVM (C7)2GE10Me : {"C": 1.0, "gamma": 1.0002302850208247}

RBF SVM (C7)2GE6Me : {"C": 1.0, "gamma": 1.0002302850208247}

RBF SVM (C7)2GE8Me : {"C": 1.0, "gamma": 1.0002302850208247}

RBF SVM (C8)2GE8Me : `{"C": 1.0, "gamma": 1.0002302850208247}`

RBF SVM C8E4 : `{"C": 1.0, "gamma": 1.0002302850208247}`

RBF SVM C8E6 : `{"C": 1.0, "gamma": 1.0002302850208247}`

C Gap Filling Predictions

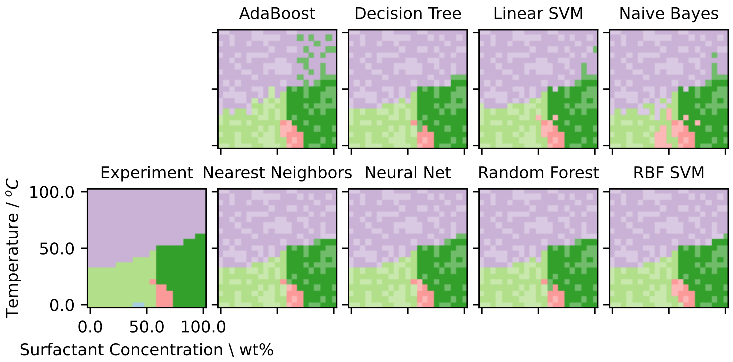

Figure S34: $(C_6)_2GE_8Me$

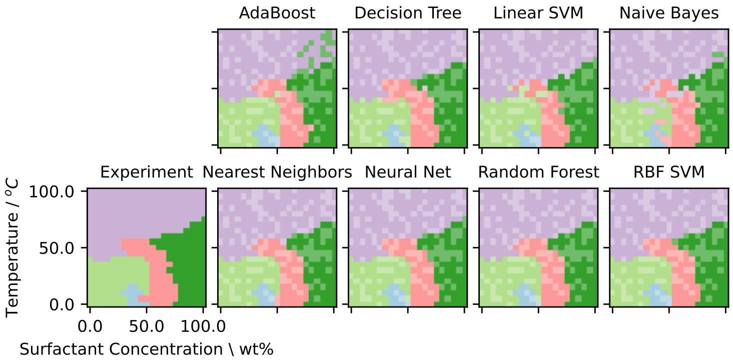

Figure S35: $(C_7)_2GE_{10}Me$

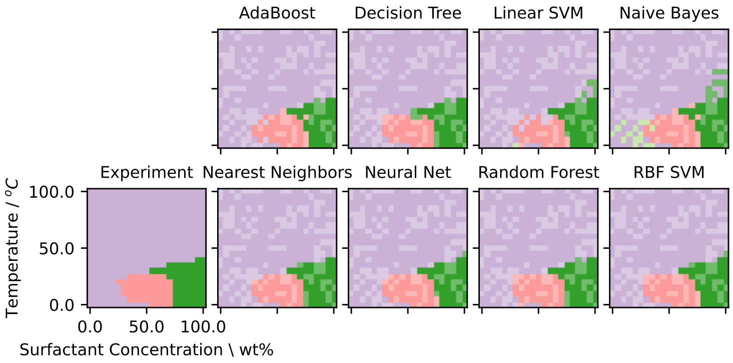

Figure S36: $(C_7)_2GE_6Me$

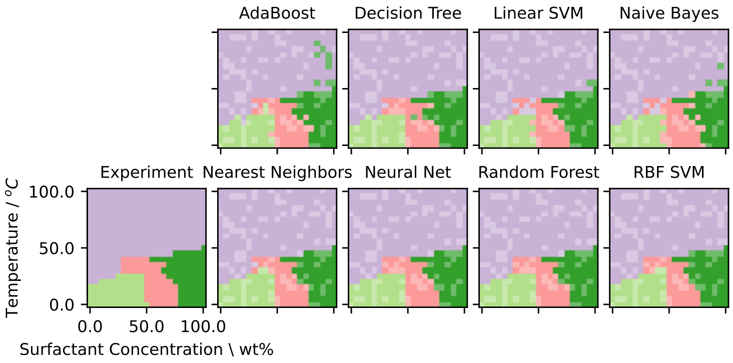

Figure S37: $(C_7)_2GE_8Me$

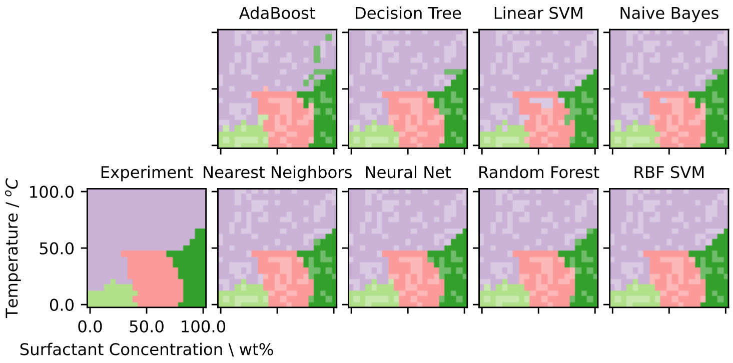

Figure S38: $(C_8)_2GE_8Me$

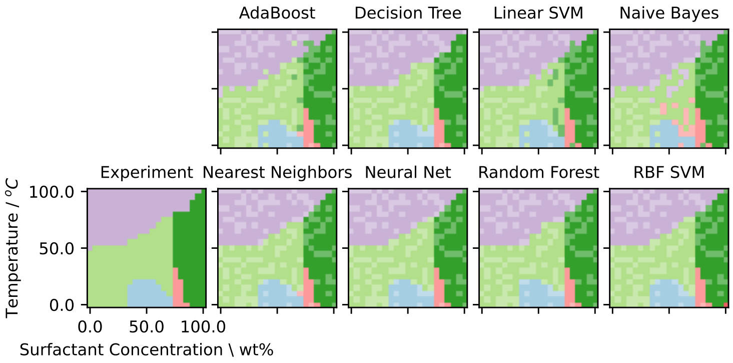

Figure S39: $C_{10}E_5$

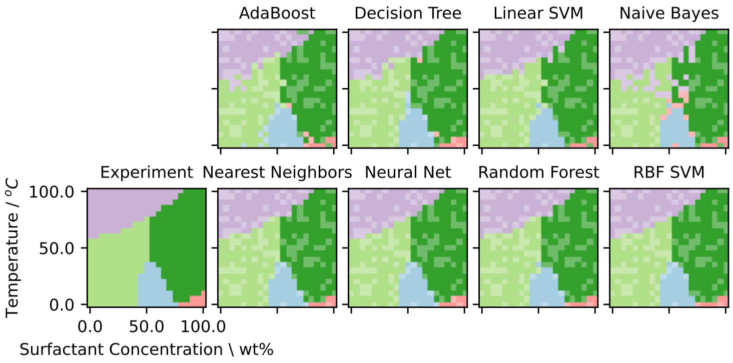

Figure S40: $C_{10}E_6$

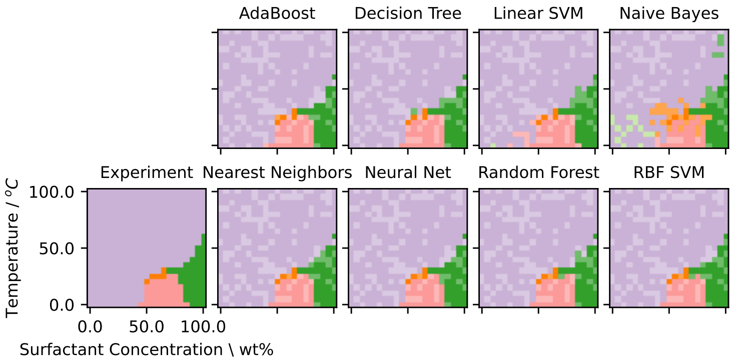

Figure S41: $C_{12}E_2$

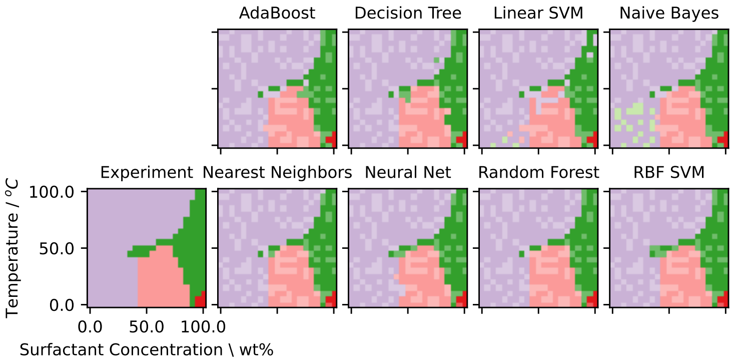

Figure S42: $C_{12}E_3$

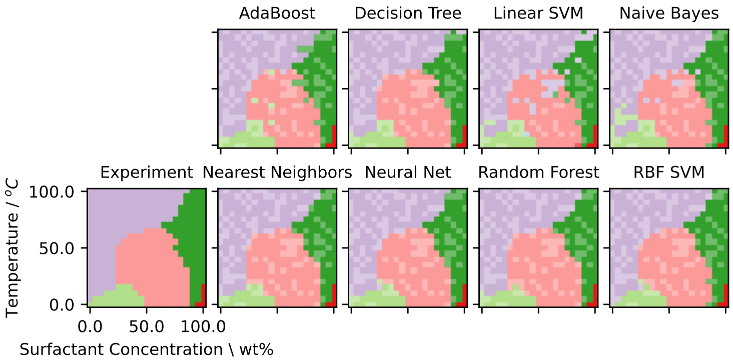

Figure S43: $C_{12}E_4$

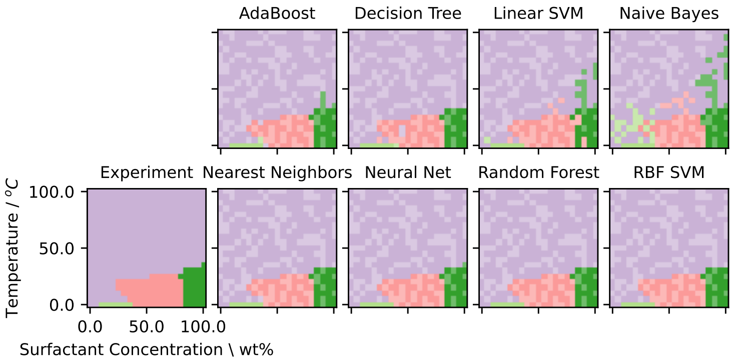

Figure S44: $C_{12}E_4Me$

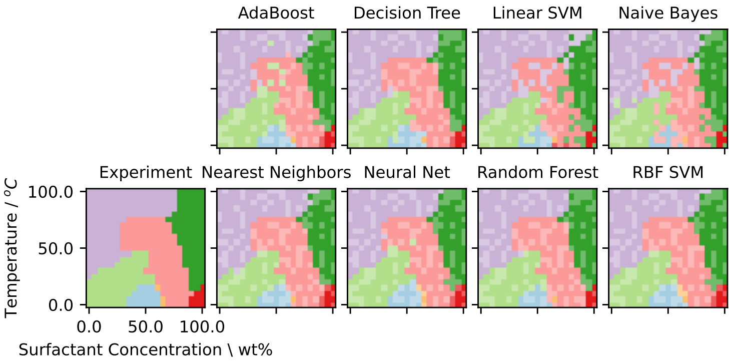

Figure S45: $C_{12}E_5$

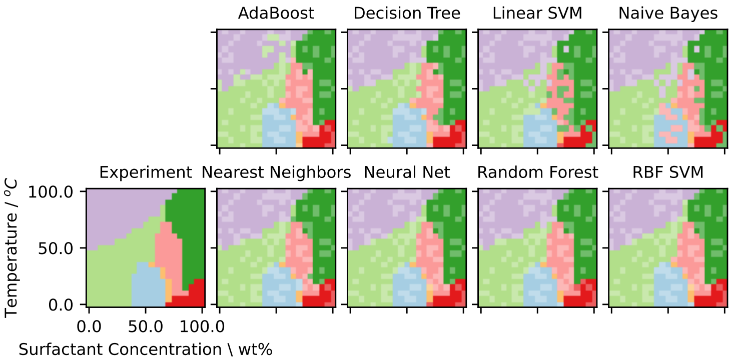

Figure S46: $C_{12}E_6$

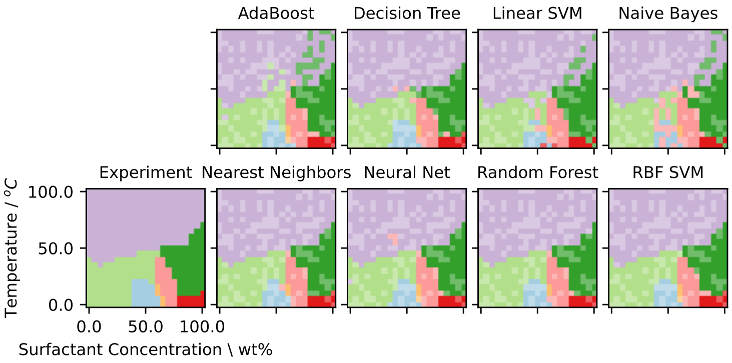

Figure S47: $C_{12}E_6Me$

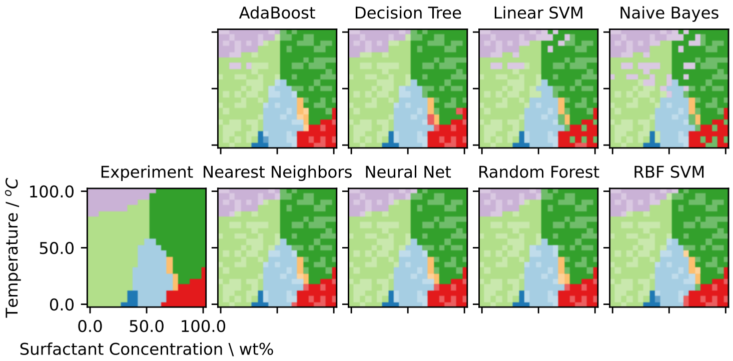

Figure S48: $C_{12}E_8$

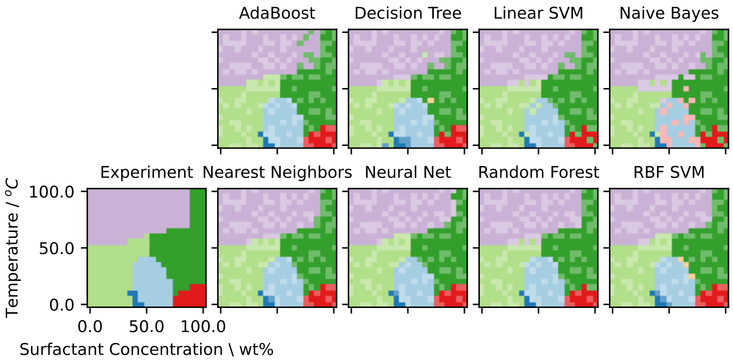

Figure S49: $C_{12}E_8Me$

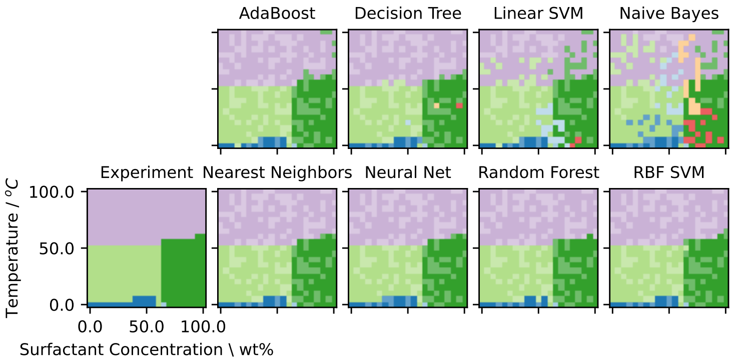

Figure S50: $C_{12}G(E_4Me)_2$

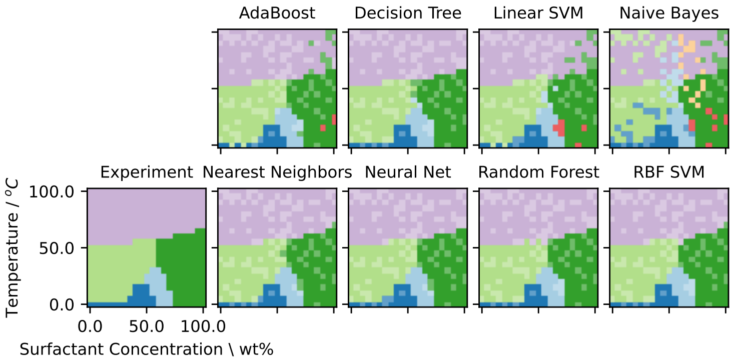

Figure S51: $C_{14}G(E_4Me)_2$

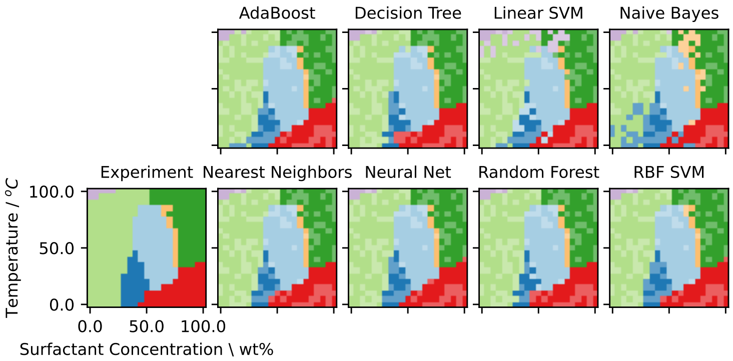

Figure S52: $C_{16}E_{12}$

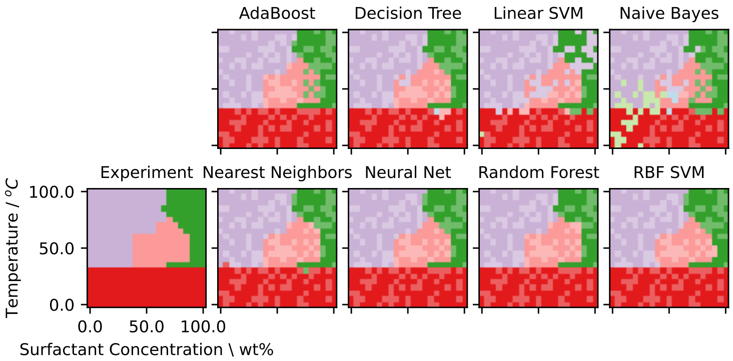

Figure S53: $C_{16}E_4$

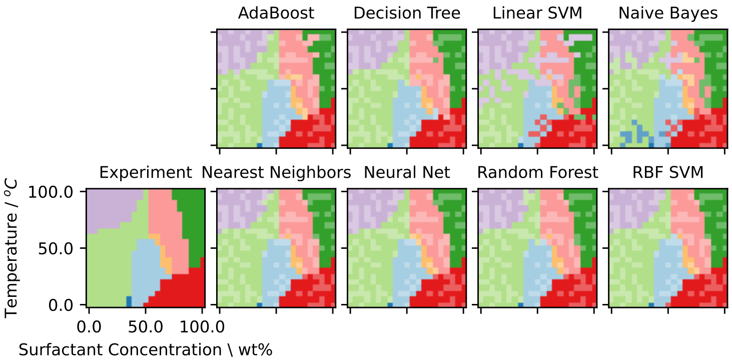

Figure S54: $C_{16}E_8$

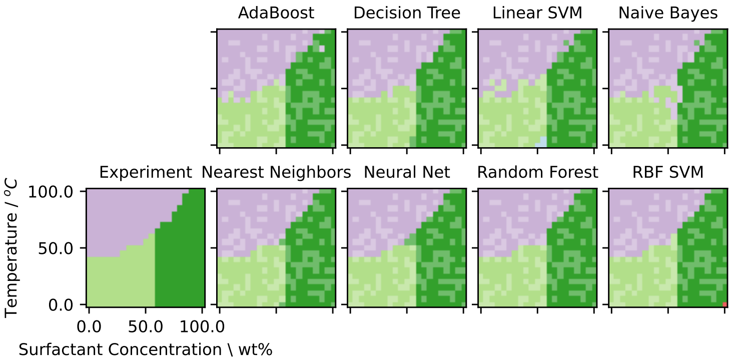

Figure S55: C_8E_4

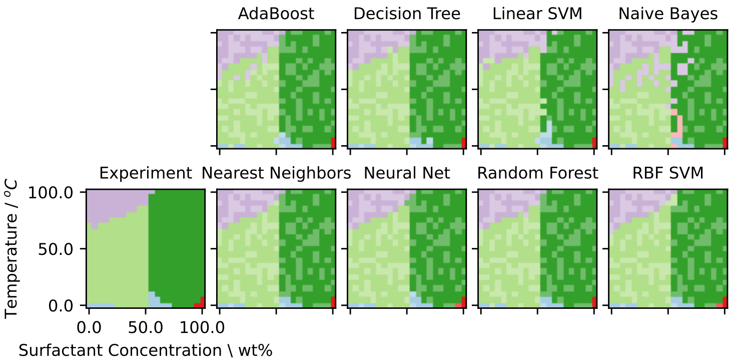

Figure S56: C_8E_6

D *De novo* phase diagram prediction

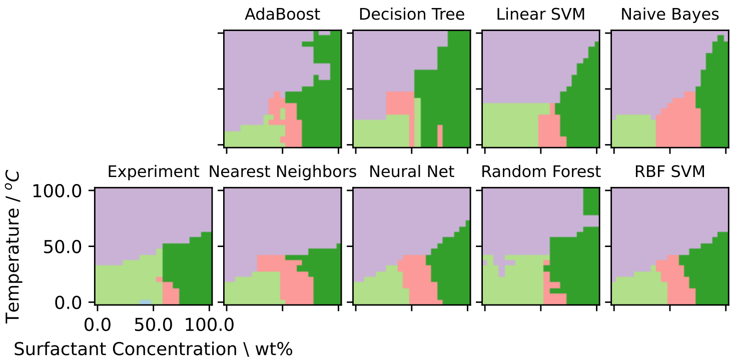

Figure S57: $(C_6)_2GE_8Me$

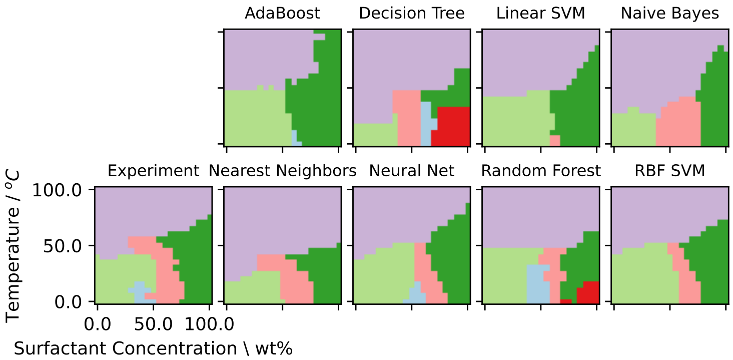

Figure S58: $(C_7)_2GE_{10}Me$

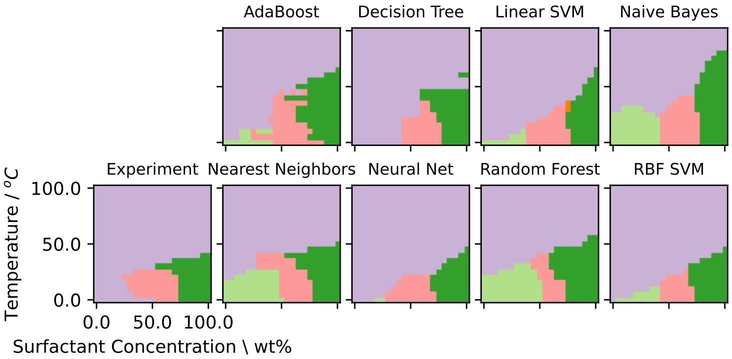

Figure S59: $(C_7)_2GE_6Me$

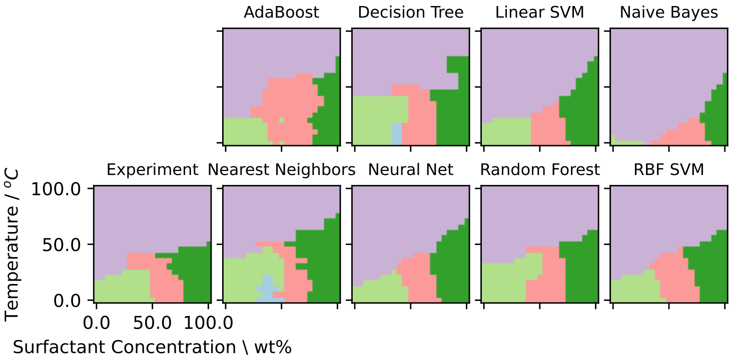

Figure S60: $(C_7)_2GE_8Me$

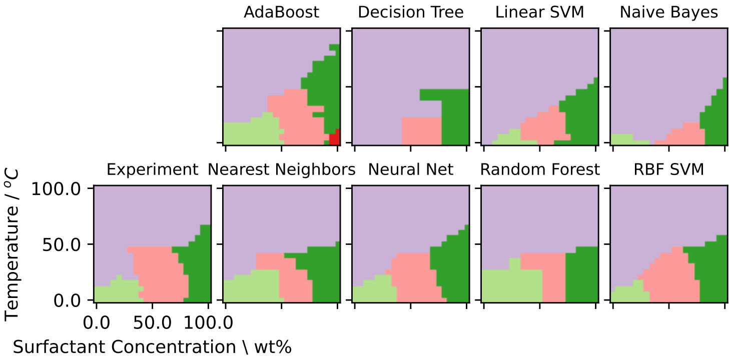

Figure S61: $(C_8)_2GE_8Me$

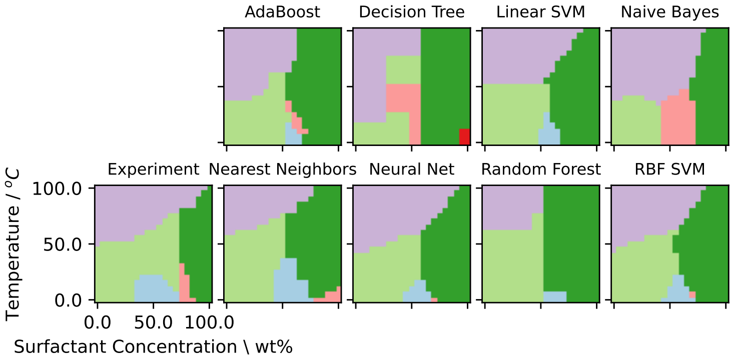

Figure S62: $C_{10}E_5$

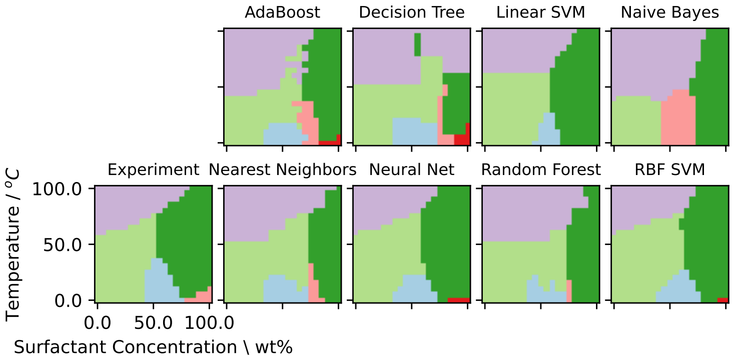

Figure S63: $C_{10}E_6$

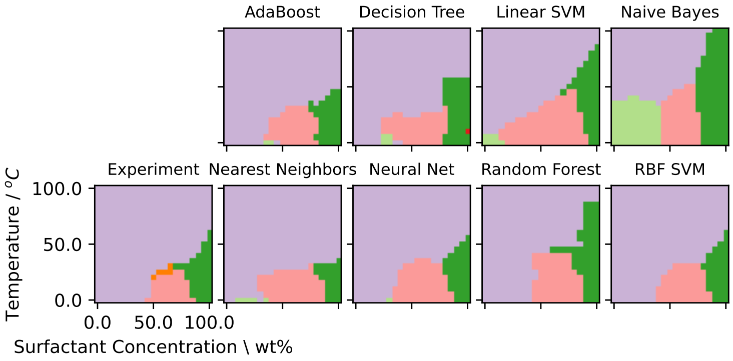

Figure S64: $C_{12}E_2$

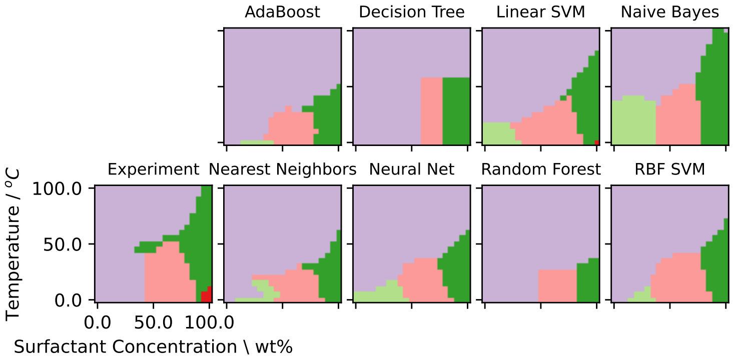

Figure S65: $C_{12}E_3$

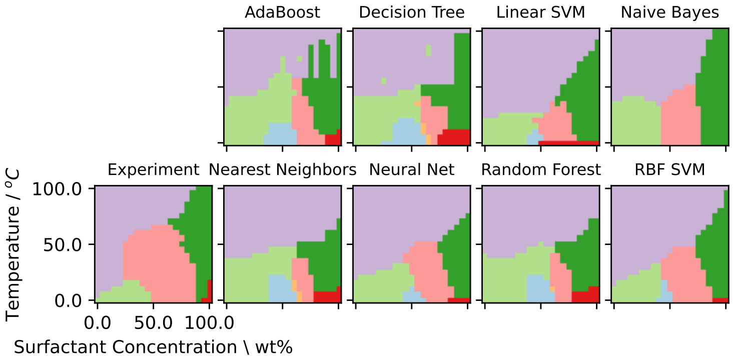

Figure S66: $C_{12}E_4$

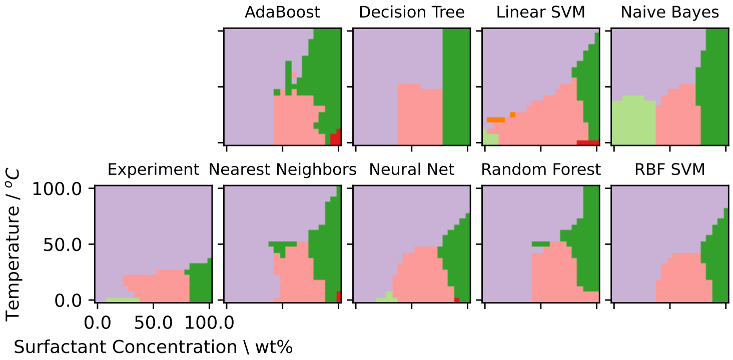

Figure S67: $C_{12}E_4Me$

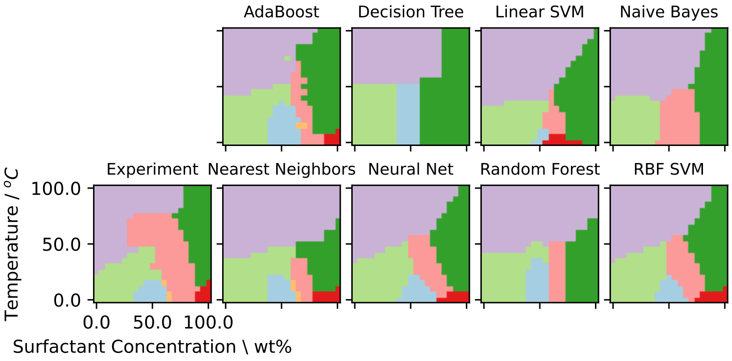

Figure S68: $C_{12}E_5$

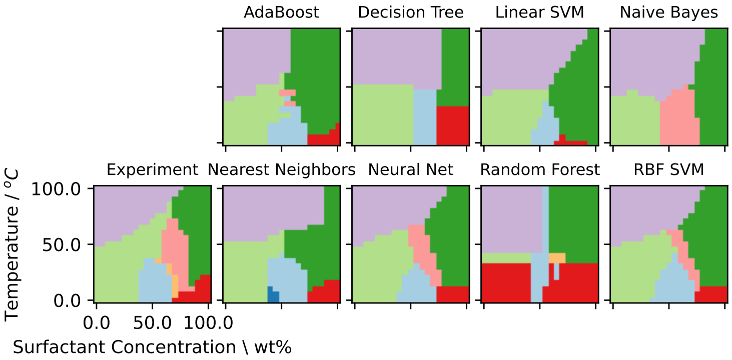

Figure S69: $C_{12}E_6$

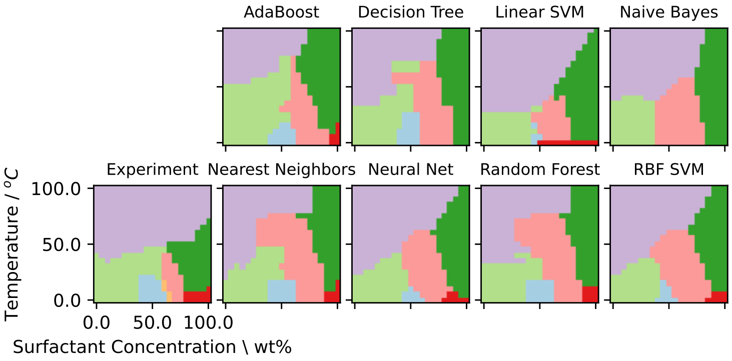

Figure S70: $C_{12}E_6Me$

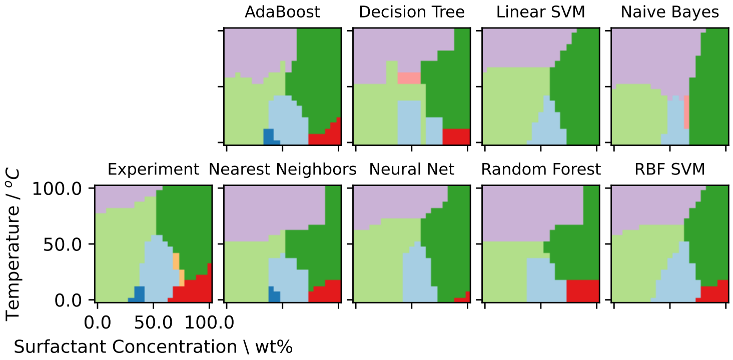

Figure S71: $C_{12}E_8$

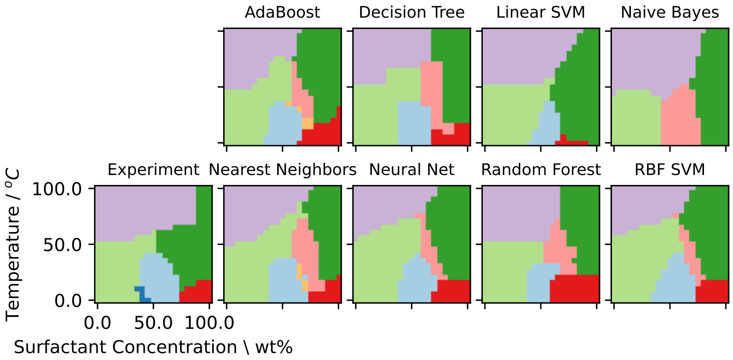

Figure S72: $C_{12}E_8Me$

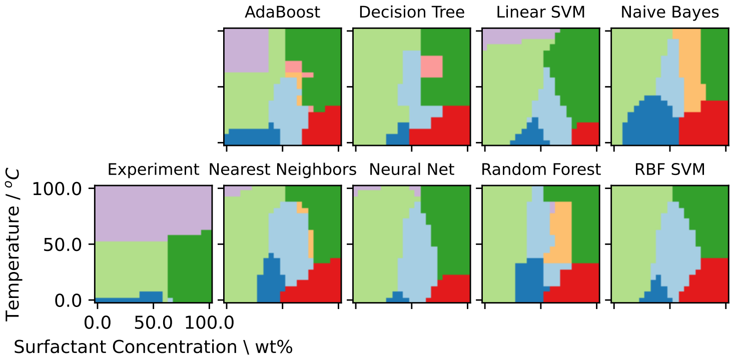

Figure S73: $C_{12}G(E_4Me)_2$

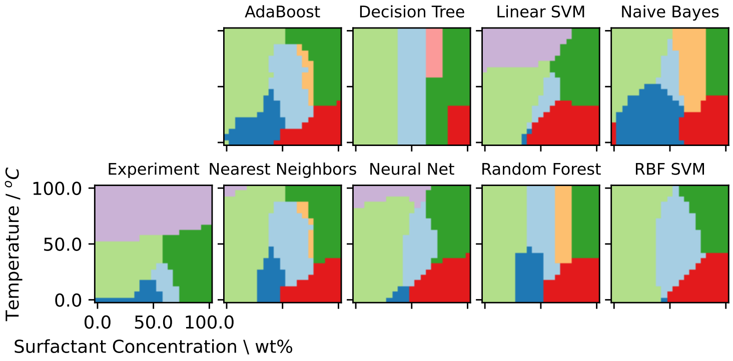

Figure S74: $C_{14}G(E_4Me)_2$

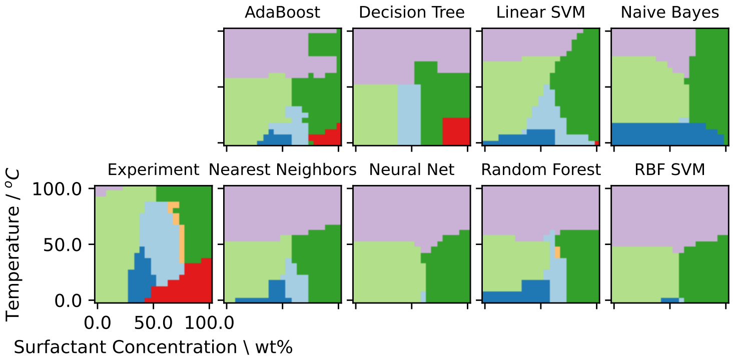

Figure S75: $C_{16}E_{12}$

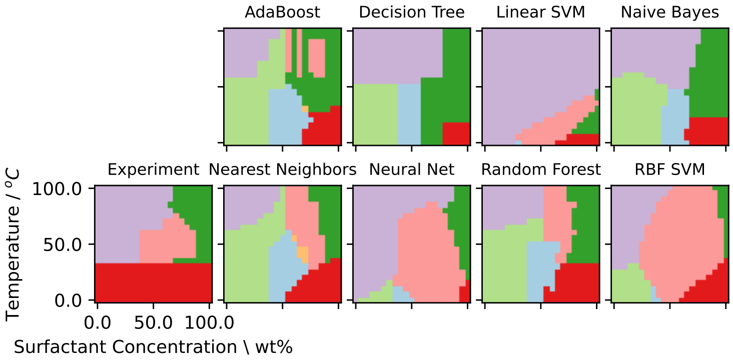

Figure S76: $C_{16}E_4$

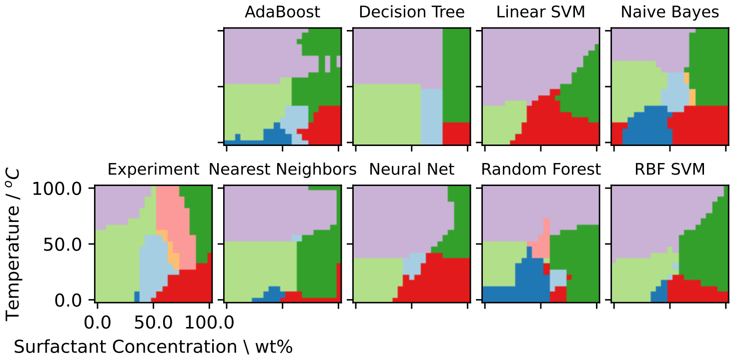

Figure S77: $C_{16}E_8$

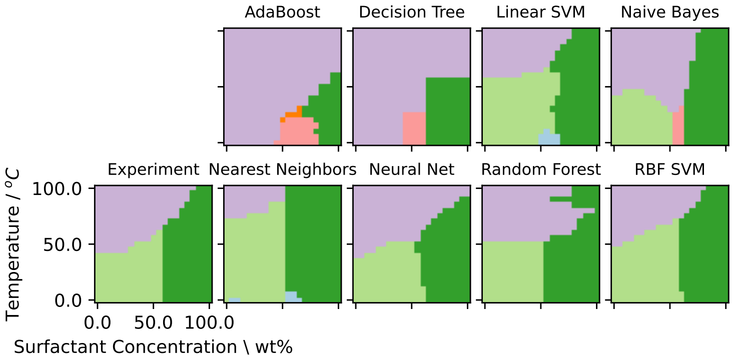

Figure S78: C_8E_4

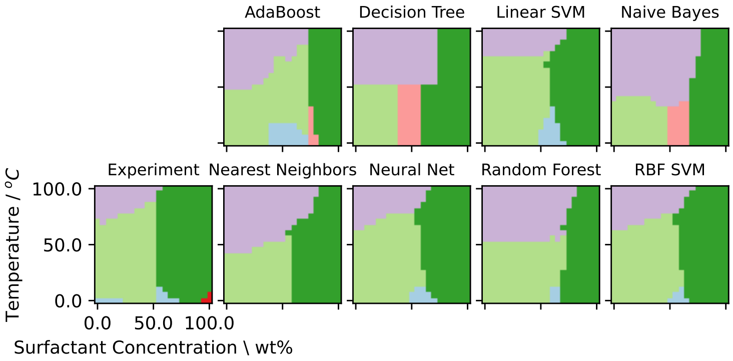

Figure S79: C_8E_6

E Confusion plots

E.1 Confusion Plots: Methods

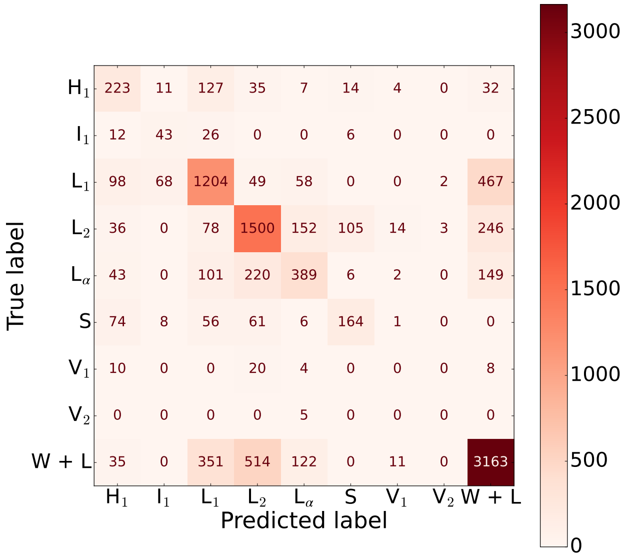

Figure S80: Adaboost Confusion Matrix

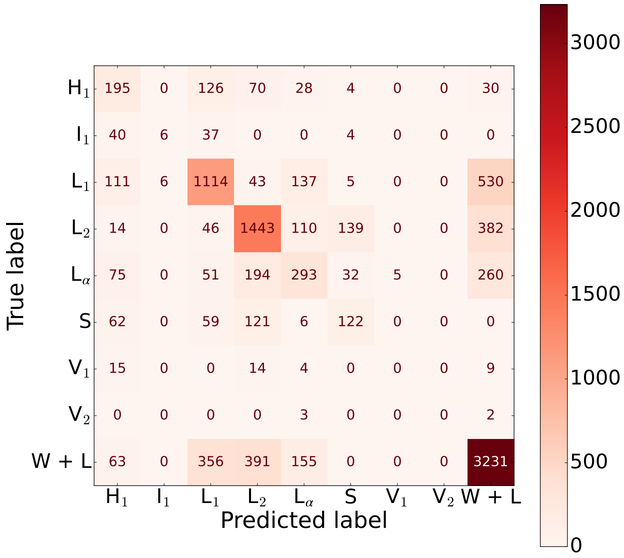

Figure S81: Decision-Tree Confusion Matrix

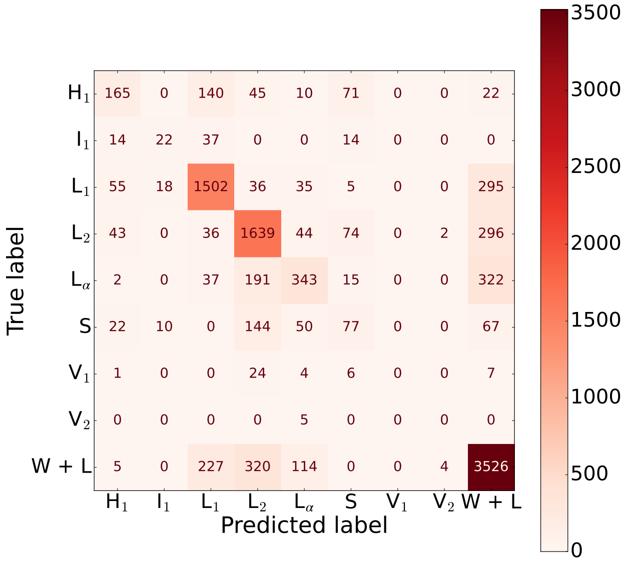

Figure S82: Linear Support Vector Machine Confusion Matrix

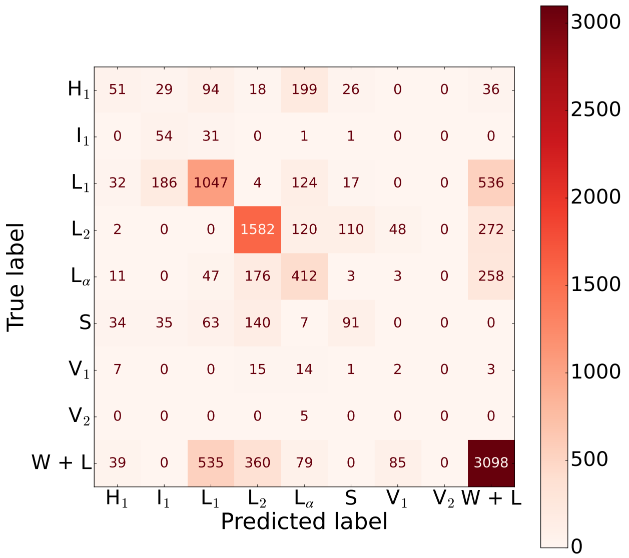

Figure S83: Naive Bayes Confusion Matrix

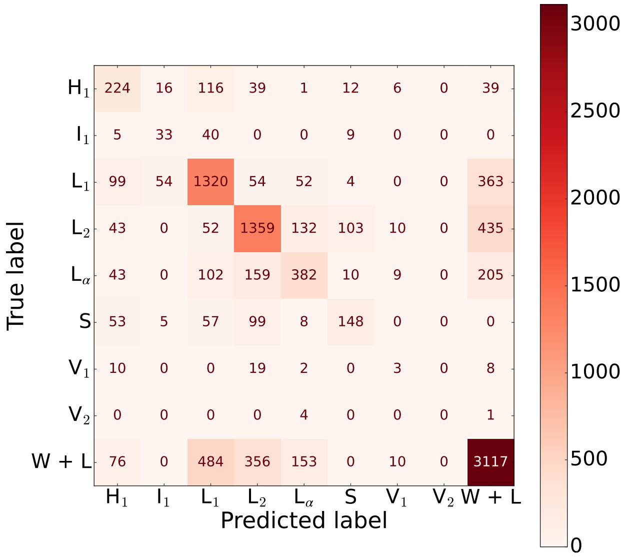

Figure S84: Nearest Neighbours Confusion Matrix

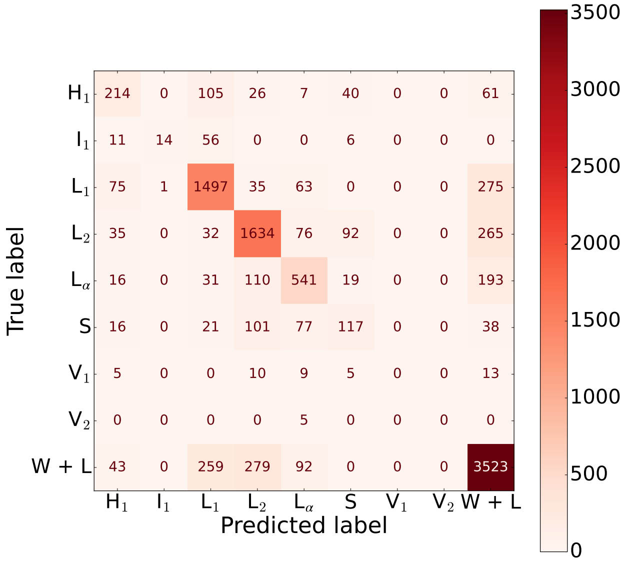

Figure S85: Neural Network Confusion Matrix

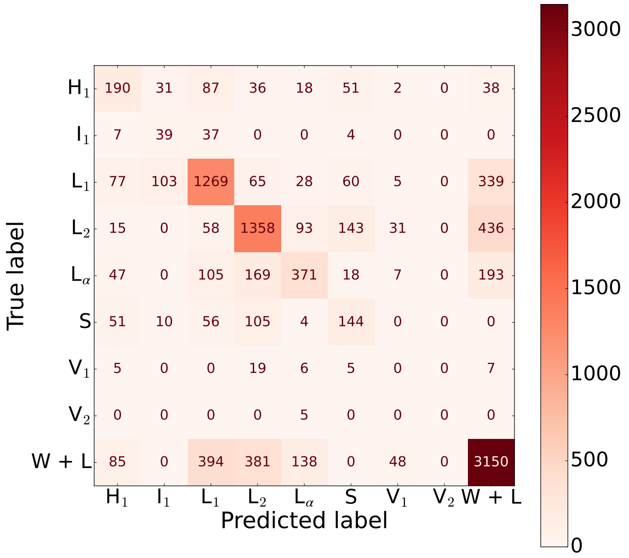

Figure S86: Random Forest Confusion Matrix

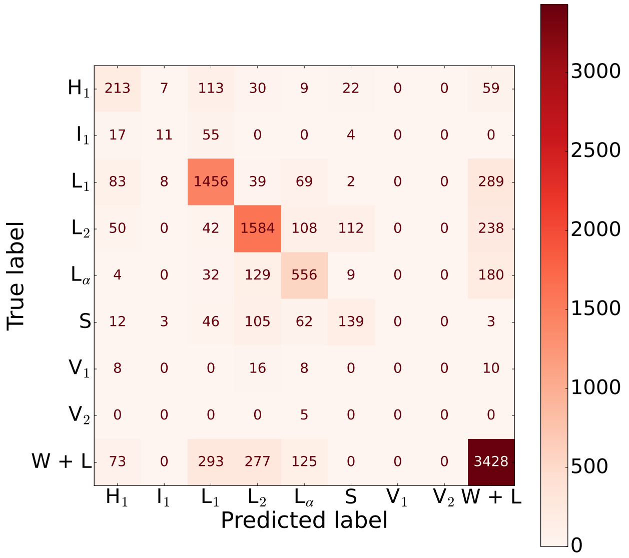

Figure S87: Radial Basis Function Support Vector Machine Confusion Matrix

E.2 Confusion Plots: Surfactants

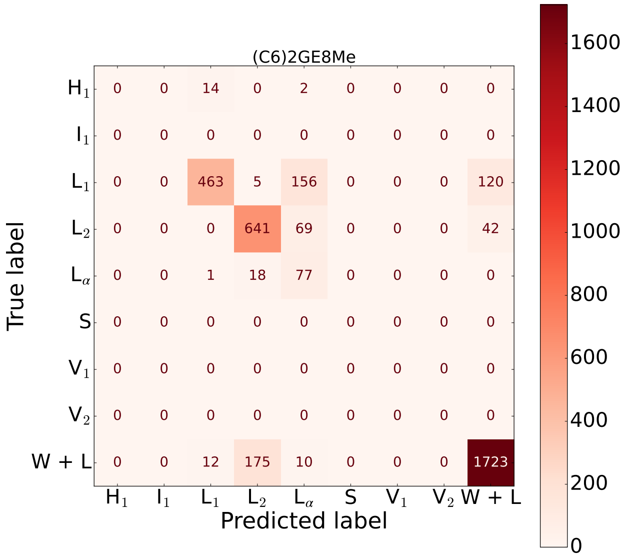

Figure S88: $(C_6)_2GE_8Me$

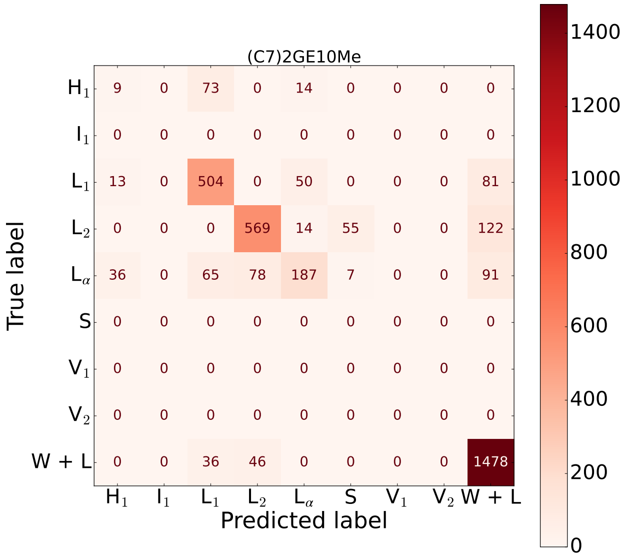

Figure S89: $(C_7)_2GE_{10}Me$

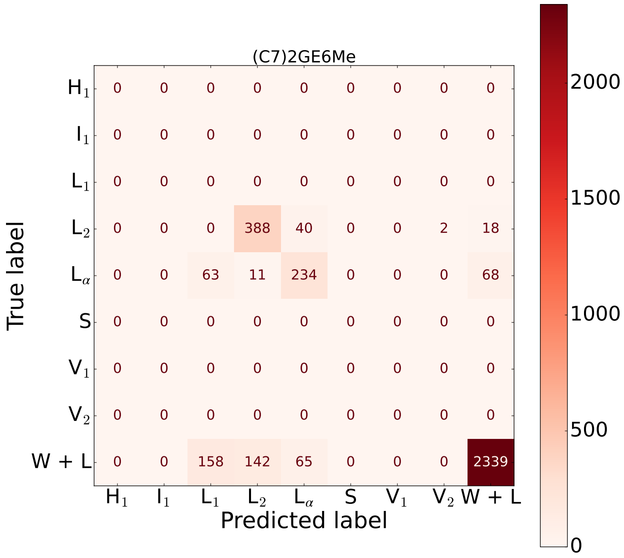

Figure S90: $(C_7)_2GE_6Me$

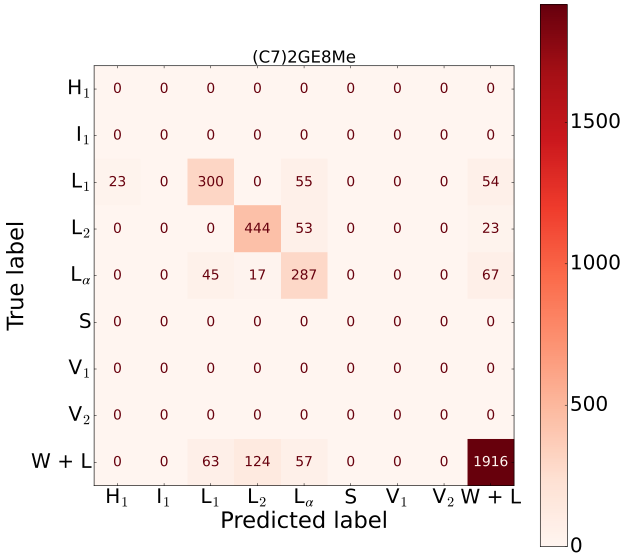

Figure S91: $(C_7)_2GE_8Me$

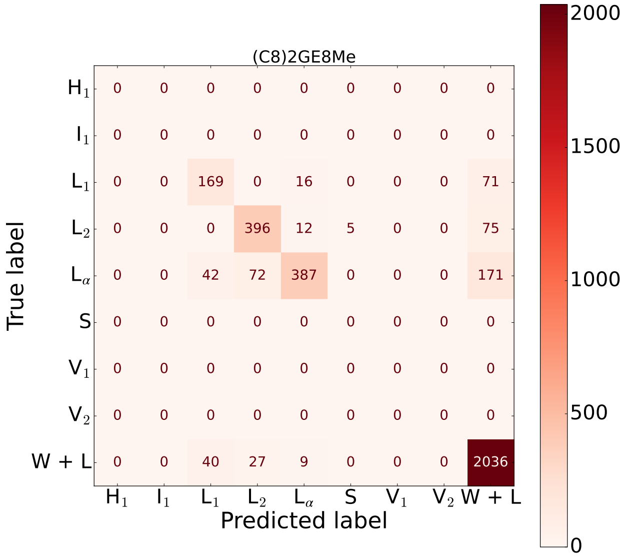

Figure S92: $(C_8)_2GE_8Me$

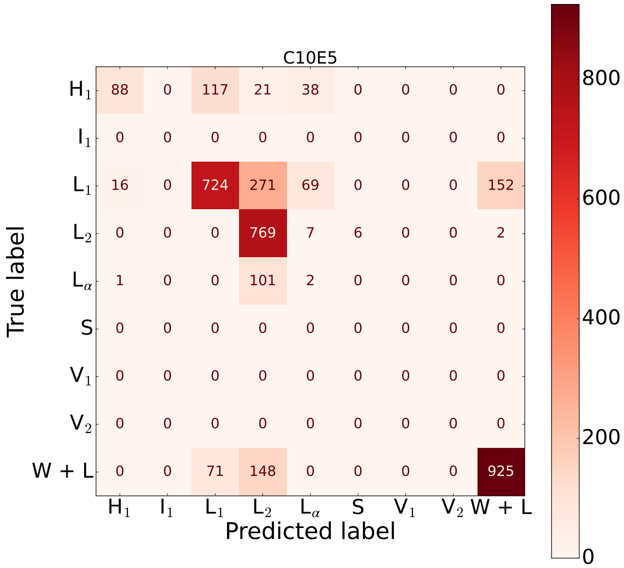

Figure S93: $C_{10}E_5$

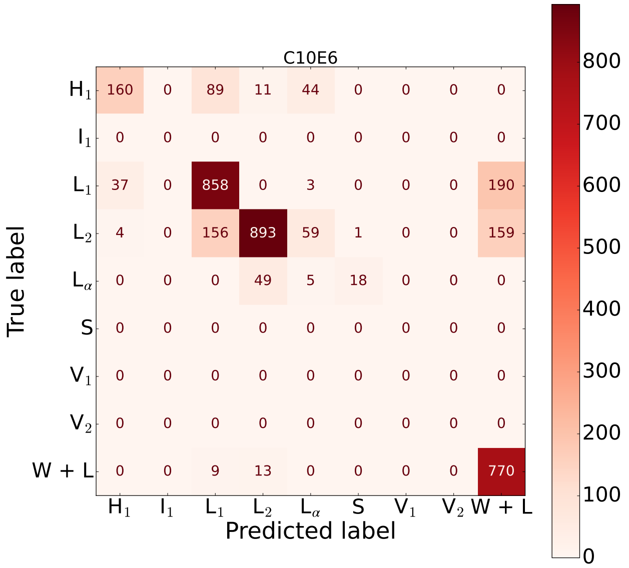

Figure S94: $C_{10}E_6$

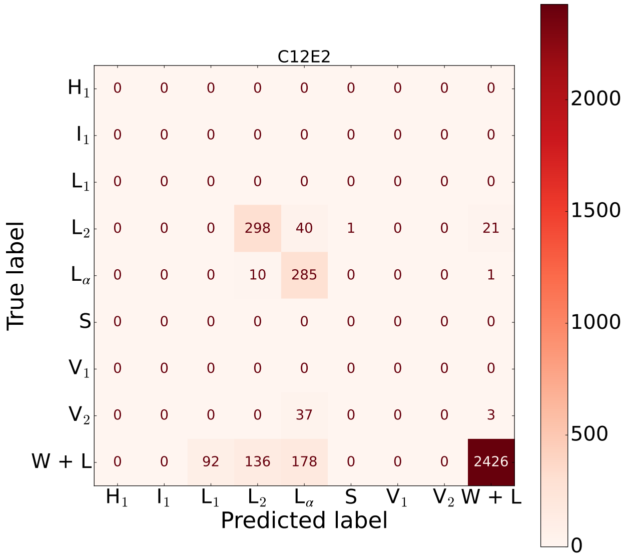

Figure S95: $C_{12}E_2$

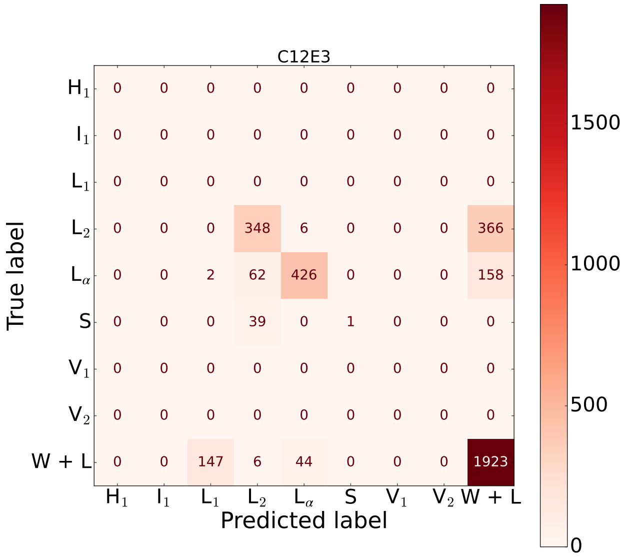

Figure S96: $C_{12}E_3$

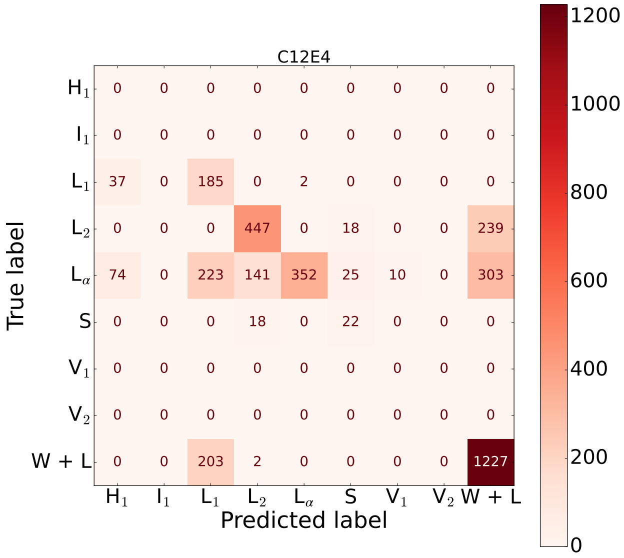

Figure S97: $C_{12}E_4$

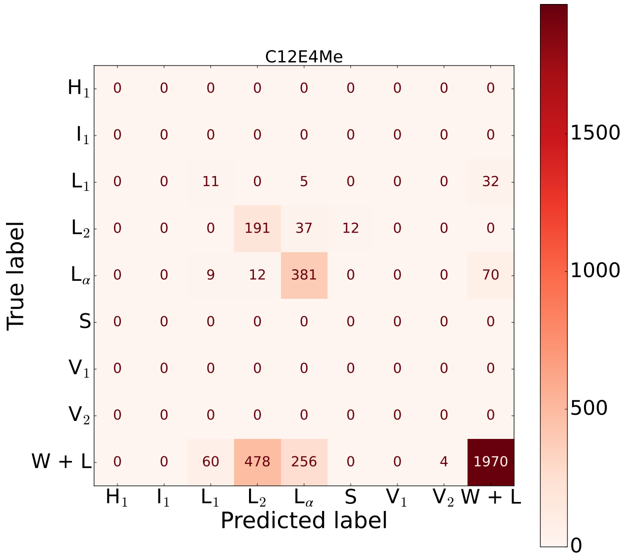

Figure S98: $C_{12}E_4Me$

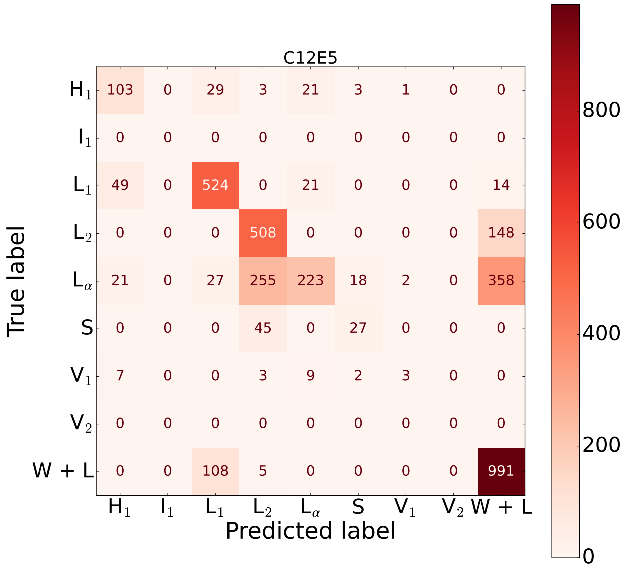

Figure S99: $C_{12}E_5$

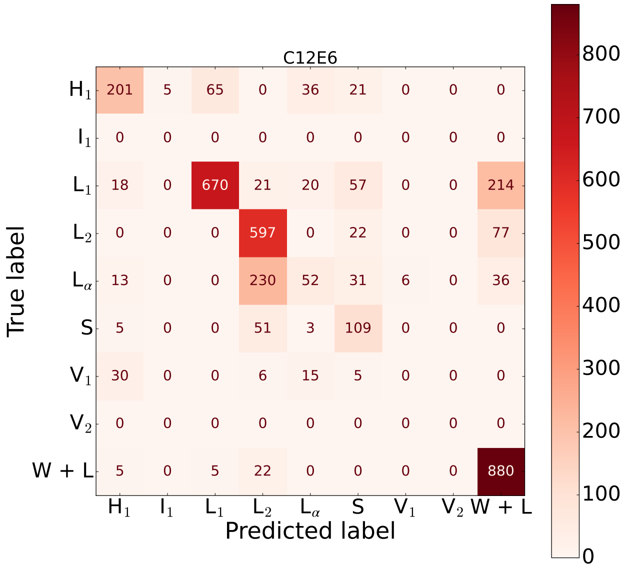

Figure S100: $C_{12}E_6$

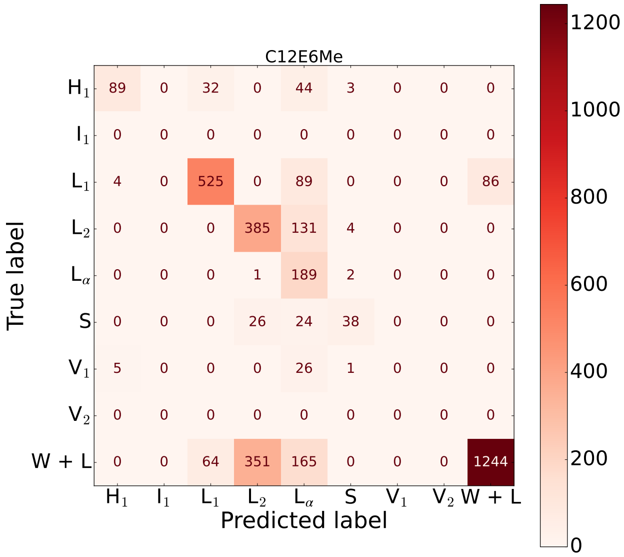

Figure S101: $C_{12}E_6Me$

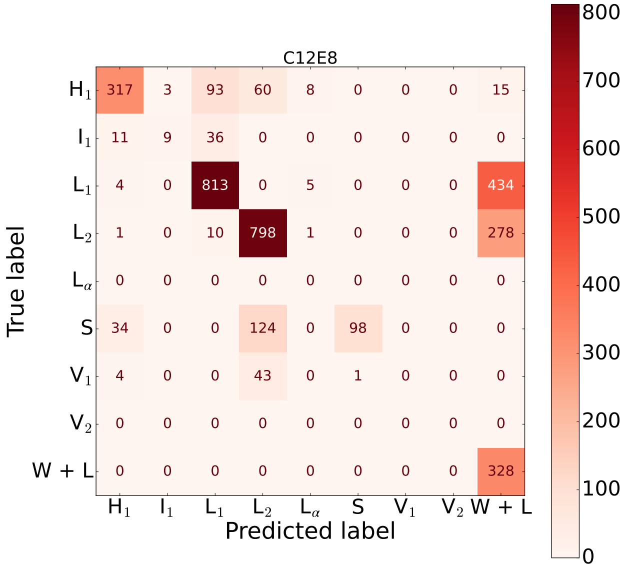

Figure S102: $C_{12}E_8$

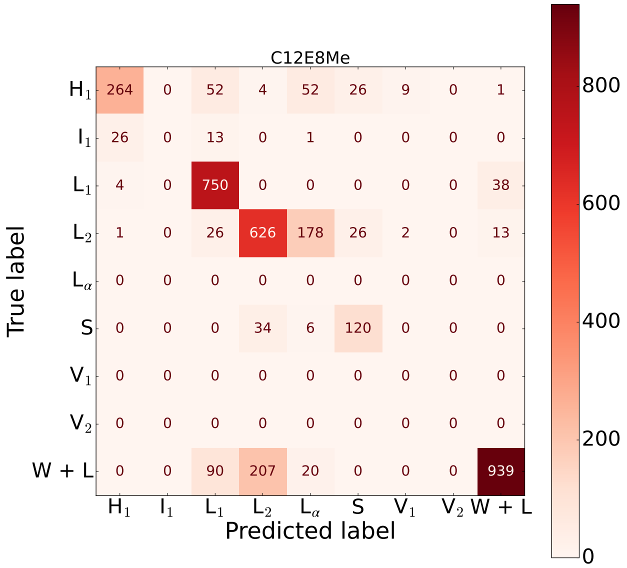

Figure S103: $C_{12}E_8Me$

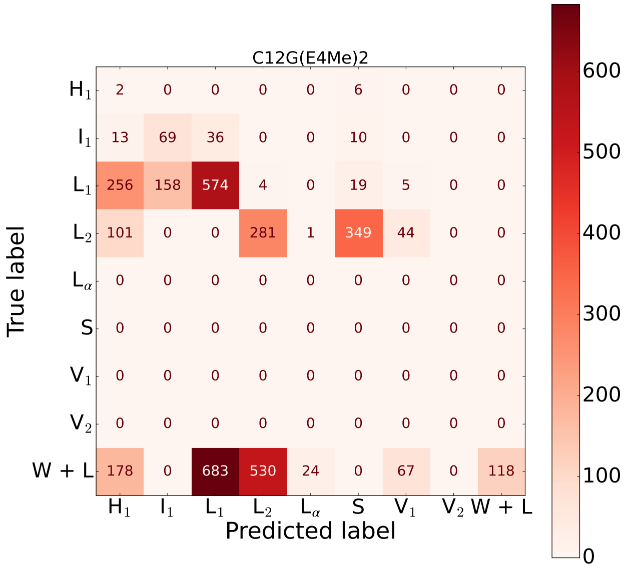

Figure S104: $C_{12}G(E_4Me)_2$

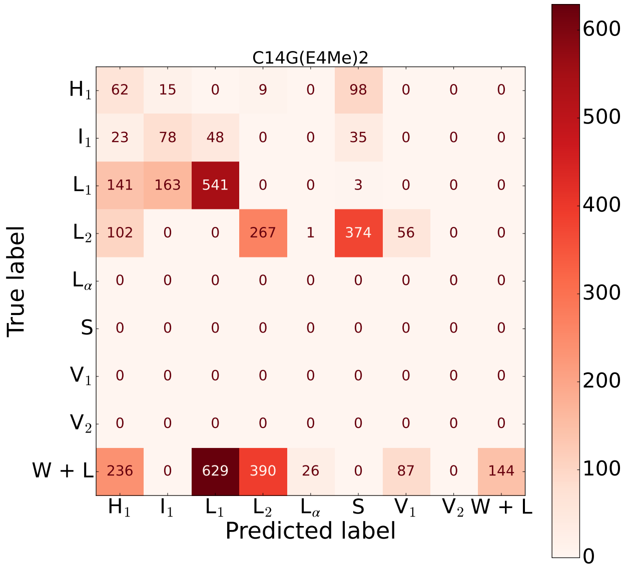

Figure S105: $C_{14}G(E_4Me)_2$

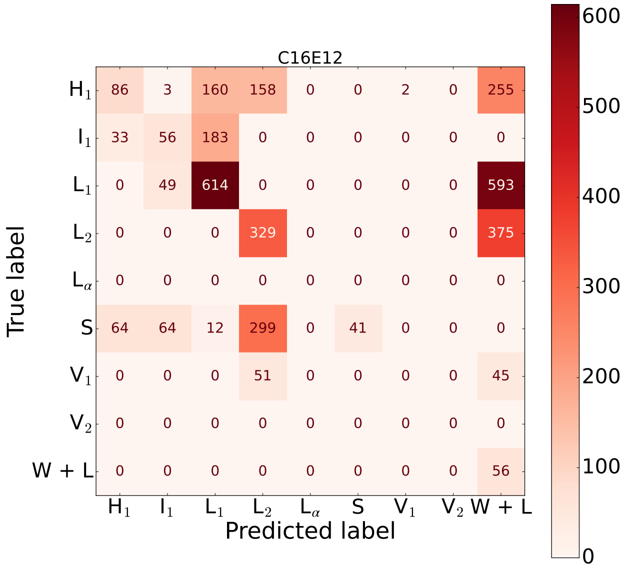

Figure S106: $C_{16}E_{12}$

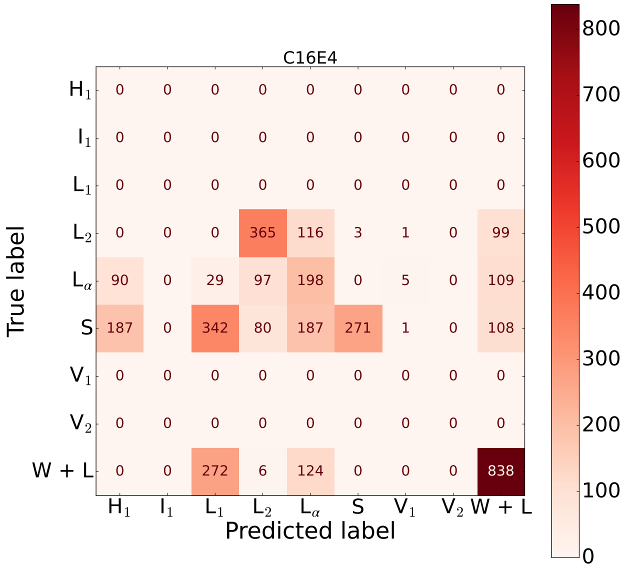

Figure S107: $C_{16}E_4$

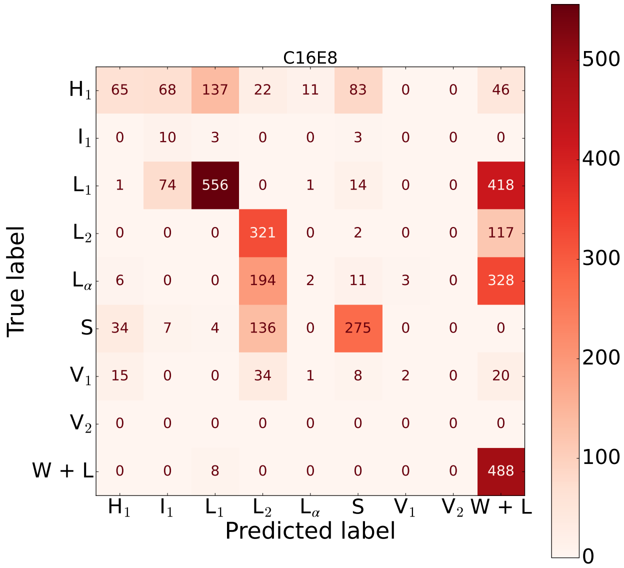

Figure S108: $C_{16}E_8$

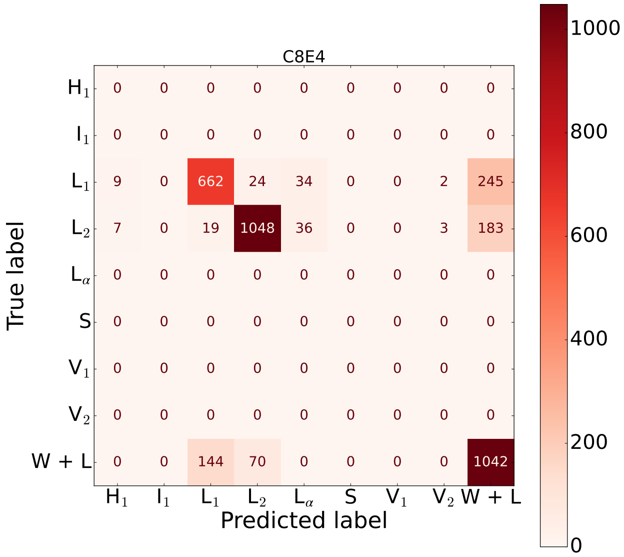

Figure S109: C_8E_4

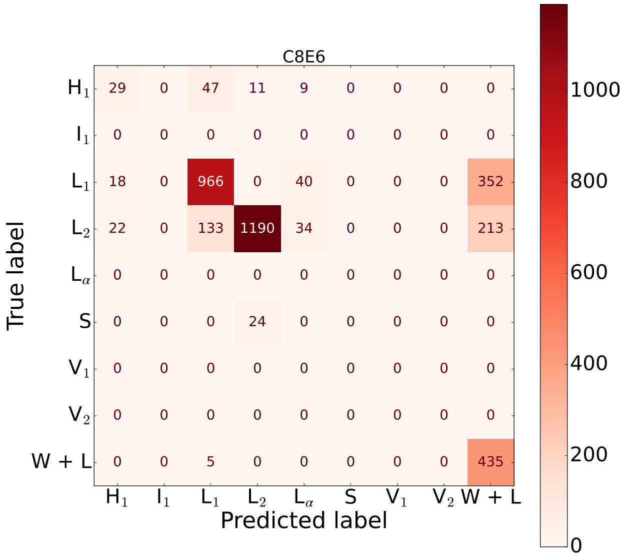

Figure S110: C_8E_6

F Correlation between regression metrics and maximum similarity

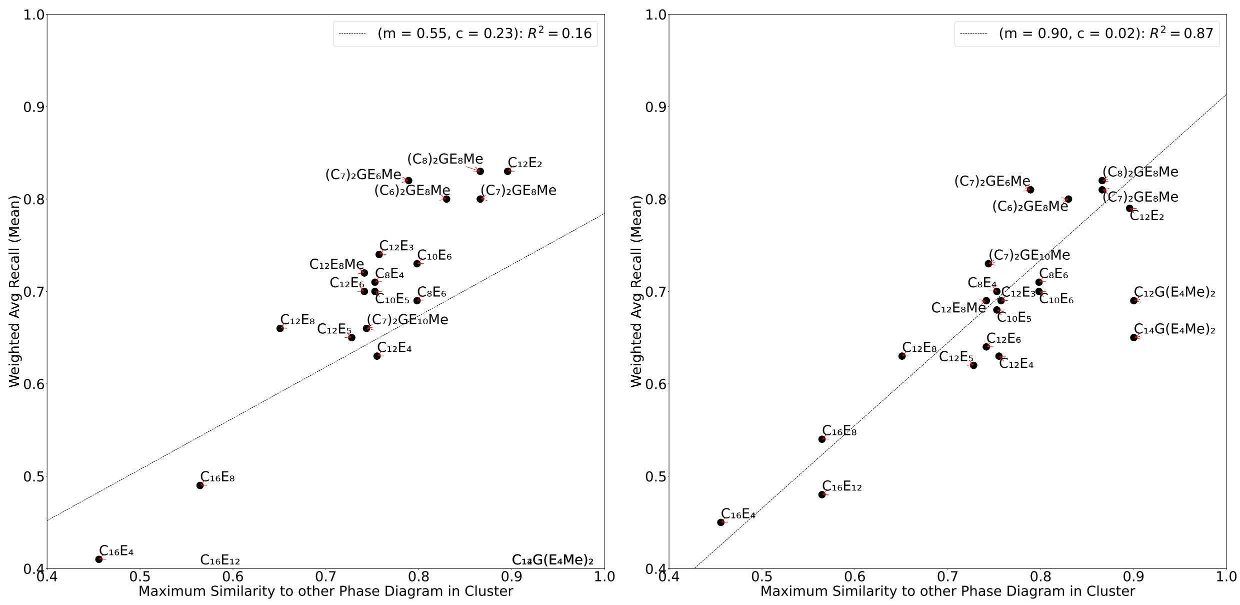

(a) Without Additional 'shape' feature (b) With additional 'shape' feature

Figure S111: Correlation between the maximum non-diagonal Similarity Matrix element and the Quality of prediction. The linear regression line is of form $y = mx + c$.

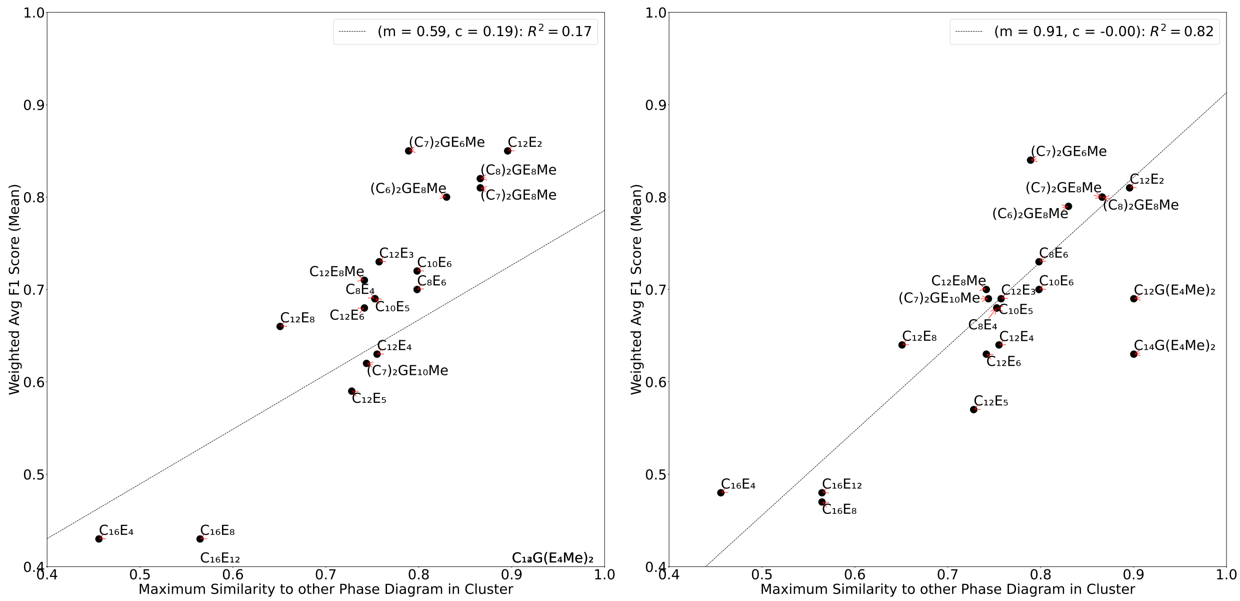

(a) Without Additional 'shape' feature

(b) With additional 'shape' feature

Figure S112: Correlation between the maximum non-diagonal Similarity Matrix element and the Quality of prediction. The linear regression line is of form $y = mx + c$.

G Laboratory Sampling Scenarios

In this section of the supporting information we expand the information presented in the main text corresponding to Section 4.5. In Figure S113 we elaborate pictorially on the main text to illustrate the different sampling scenarios. In Figures S114-S116 and S117-S119 we show the predicted phase diagram against the experimental phase diagram for $(C_8)_2GE_8Me$ and $C_{12}E_5$ respectively.

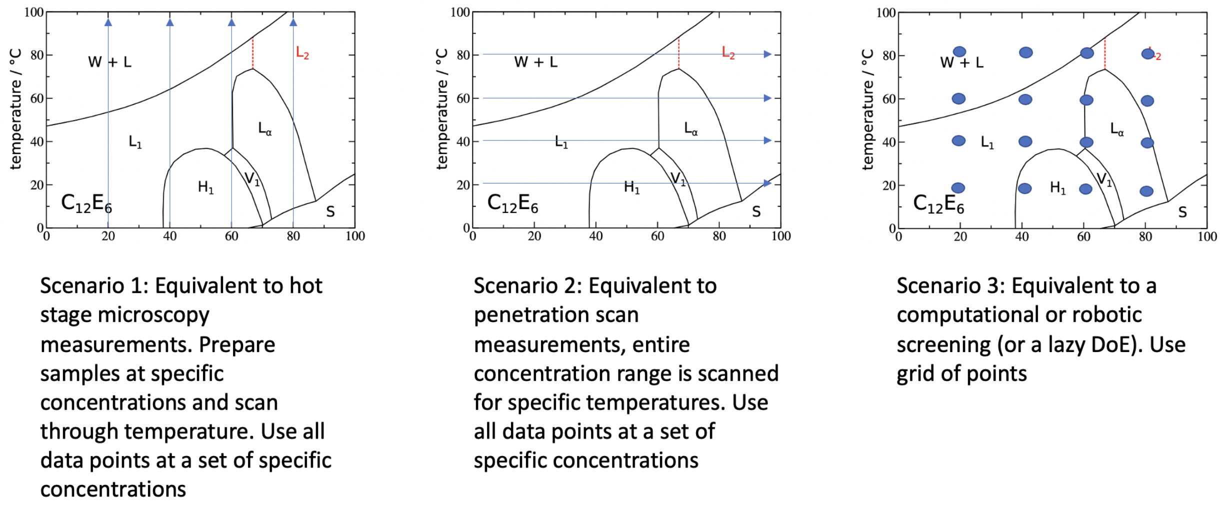

Figure S113: The three sampling approaches employed in Section 4.5 of the main text. $C_{12}E_6$ phase diagram reproduced (adapted) with permission from Mitchell *et al.*[1]. Copyright 1983 Royal Society of Chemistry.

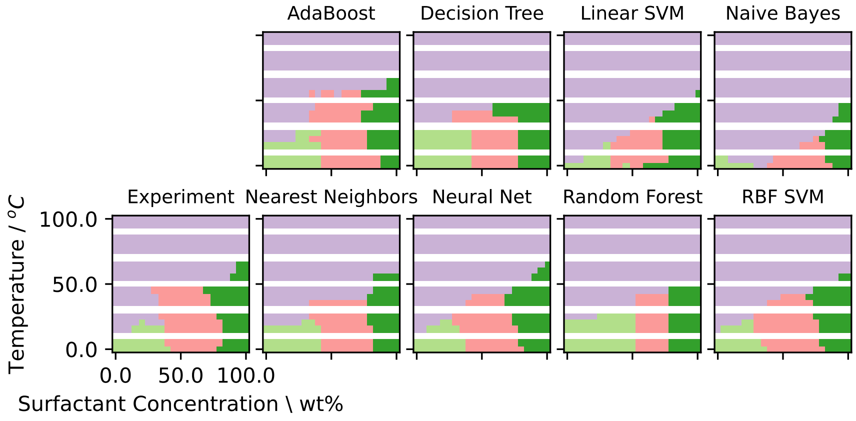

Figure S114: $(C_8)_2GE_8Me$ - Penetration Scanning Scenario (A1)

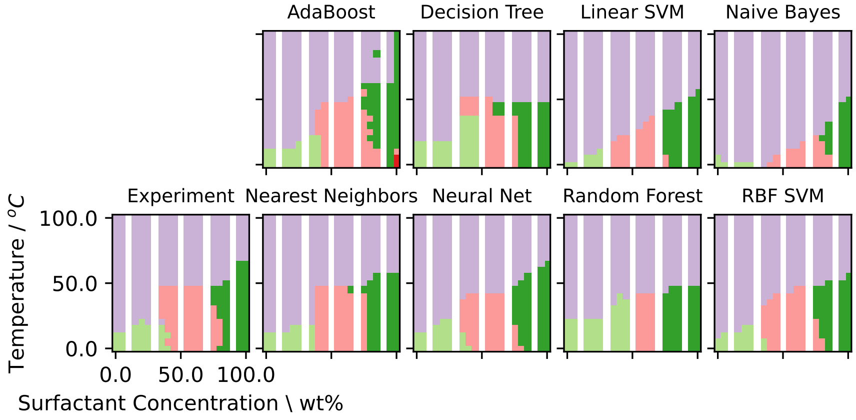

Figure S115: $(C_8)_2GE_8Me$ - Hot Stage Microscopy Scenario (A2)

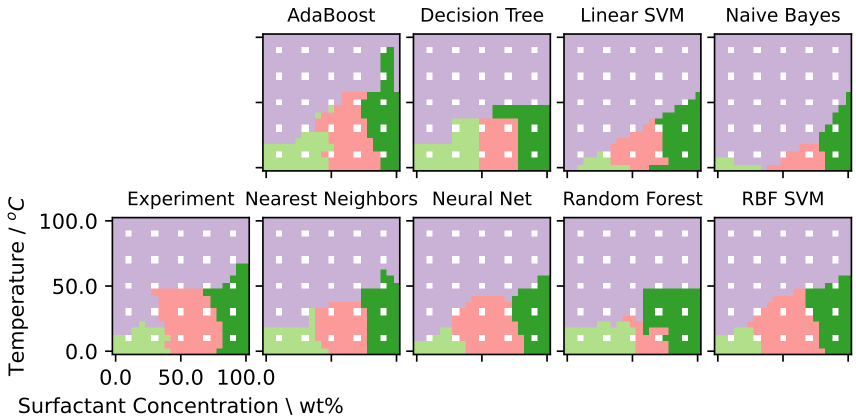

Figure S116: $(C_8)_2GE_8Me$ - Sparse Scenario (A3)

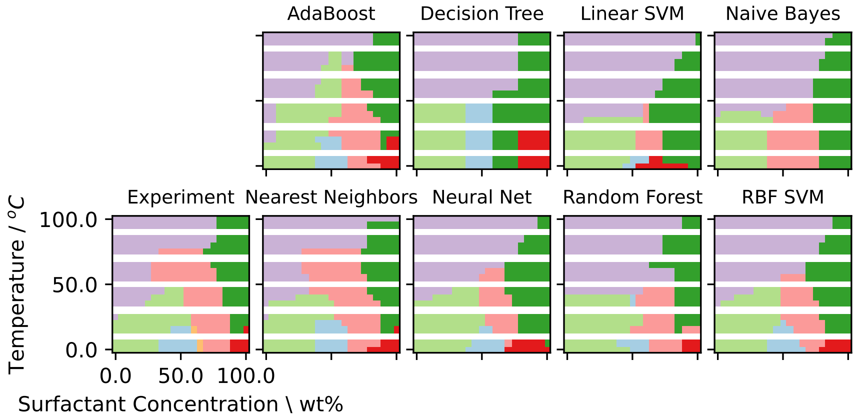

Figure S117: $C_{12}E_5$ - Penetration Scanning Scenario (A1)

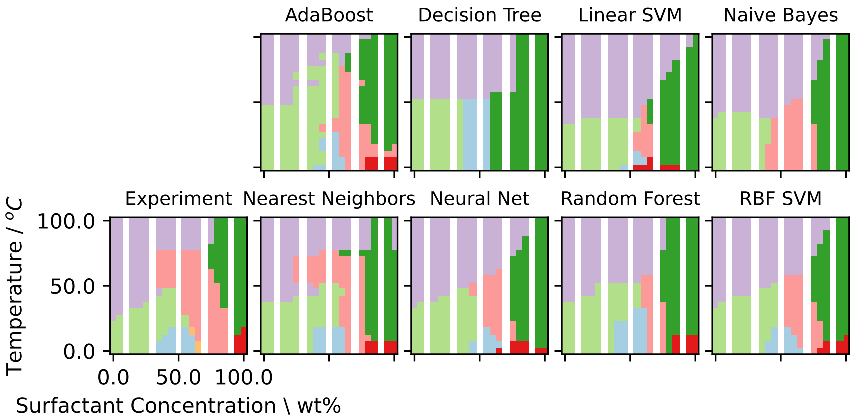

Figure S118: $C_{12}E_5$ - Hot Stage Microscopy Scenario (A2)

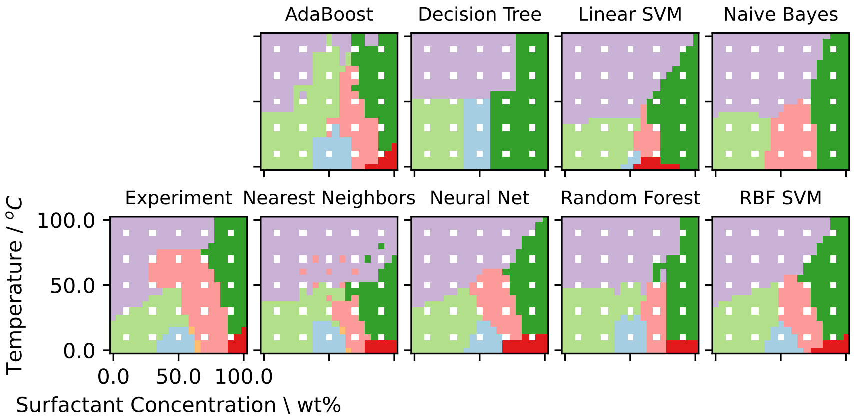

Figure S119: $C_{12}E_5$ - Sparse Scenario (A3)

References

(1) Mitchell, D. J.; Tiddy, G. J. T.; Waring, L.; Bostock, T.; McDonald, M. P. Phase Behaviour of Polyoxyethylene Surfactants With Water. Mesophase Structures and Partial Miscibility (Cloud Points). *J. Chem. Soc., Faraday Trans. 1* **1983**, *79*, 975–1000.
